# Clay‐Mineral‐Coated Separators for Lithium‐Ion Batteries: Exploring the Relationships between Clay Mineral Morphology and Separator Performance

**DOI:** 10.1002/advs.202510779

**Published:** 2025-09-19

**Authors:** Inseo Ko, Seoyoung Ha, Weon Ho Shin, Jongyoung Choi, Sung Cik Mun, Yong‐Seok Choi, Jong Ho Won

**Affiliations:** ^1^ Department of Hydrogen Energy Dankook University 119 Dandae‐ro, Dongnam‐gu Cheonan‐si Chungnam 31116 Republic of Korea; ^2^ Department of Electronic Materials Engineering Kwangwoon University 20 Kwangwoon‐ro, Nowon‐gu Seoul 01897 Republic of Korea; ^3^ Battery Materials Research Center LG Chem 30 Magokjungang 10‐ro, Gangseo‐gu Seoul 07796 Republic of Korea; ^4^ Department of Materials Science and Engineering Dankook University 119 Dandae‐ro, Dongnam‐gu Cheonan‐si Chungnam 31116 Republic of Korea; ^5^ Department of Energy Engineering Dankook University 119 Dandae‐ro, Dongnam‐gu Cheonan‐si Chungnam 31116 Republic of Korea

**Keywords:** battery separators, clay minerals, coating, durability, ionic conductivity, Li‐ion batteries, Li–S batteries

## Abstract

Polyolefin separators comprising polyethylene and polypropylene have long been used in commercial Li‐ion batteries because of their electrochemical stability, robust mechanical properties, uniform pore structure, and cost‐effectiveness. However, conventional separators have limitations in withstanding harsh environmental conditions, such as elevated temperatures, owing to their poor mechanical durability. Moreover, with the development of advanced Li rechargeable batteries, these separators are required to overcome new challenges such as preventing the migration of polysulfide intermediates in Li–S batteries and inhibiting the formation of Li dendrites in Li metal batteries. Natural clay minerals have emerged as a viable solution to these issues owing to their porous structure, high mechanical strength, and abundant polysulfide‐capturing Lewis acid sites. However, the manner in which the structural characteristics of minerals influence the separator performance has not been extensively assessed, thereby impeding the development of separators for future high‐performance Li batteries. This review comprehensively outlines various clay minerals and their effects on the parameters and performance metrics of battery separators. Based on this review, a design guide is proposed for mineral‐based separators that could potentially be integrated into next‐generation Li batteries with high energy density and long‐term stability.

## Graphical abstract

This review examines how coating Li‐ion battery separators with 1D, 2D, and 3D structured natural clay minerals influences their electrochemical and mechanical performance. It classifies 15 types of clay minerals and highlights structure–property correlations to offer practical design strategies for high‐performance separators in next‐generation energy storage systems.

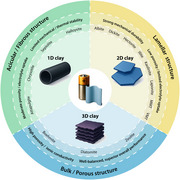

## 1 Introduction

Li‐ion batteries (LIBs) are the predominant commercialized energy‐storage systems for electric vehicles and portable electronics owing to their high energy density and prolonged service life. The success of LIBs has been attributed primarily to the use of electrode materials with highly reversible Li storage properties (such as transition metal oxides and graphitic carbon materials)^[^ ^1^, ^2^ ^]^ and secondarily to the judicious selection of electrolytes and optimizing the design of separators, which physically isolate the electrodes as well as efficiently transport Li ions between them while maintaining stability and preventing deterioration.

Unlike electrode materials, which have been a focal point in the battery field since the commercialization of LIBs in 1991,^[^ ^3^ ^]^ separators have drawn relatively less research attention. Instead of developing separators specifically for batteries, researchers employed readily available membranes that could be mass‐produced using existing technologies (such as electrospinning and^[^ ^4^ ^]^ phase inversion^[^ ^5^ ^]^) to construct early LIBs. The separators manufactured using these methods comprise pristine or composite forms of polyethylene (PE),^[^ ^6^, ^7^ ^]^ polypropylene (PP),^[^ ^8^ ^]^ poly(ethylene oxide) (PEO),^[^ ^9^, ^10^, ^11^ ^]^ polyacrylonitrile (PAN),^[^ ^9^, ^12^, ^13^ ^]^ and poly(vinylidene fluoride) (PVDF) and its copolymers.^[^ ^14^, ^15^, ^16^, ^17^, ^18^ ^]^ The separator is designed to 1) prevent direct contact between the electrodes and the associated short circuit, 2) allow Li‐ion transport, and 3) provide electronic insulation ability to block electron transmission.^[^ ^19^, ^20^ ^]^ However, these properties are insufficient for LIB operation under stringent operating conditions, such as temperatures exceeding 100 °C or evaluated mechanical pressure during battery cycling, as evidenced by recent incidents of fires in electric vehicles and electronic devices that have been reported worldwide.^[^ ^21^ ^]^ Moreover, conventional polymer separators typically fracture or shrink under such external stresses or high‐temperature environments, significantly increasing the risk of serious safety hazards such as short circuits and thermal runaway.^[^ ^22^ ^]^ Because these harsh operating conditions are a key driving force behind the development of advanced separator materials, efforts have increasingly focused on enhancing thermal stability, mechanical robustness, and electrolyte wettability through material design and structural engineering.

To address these safety issues, battery separators have been reinforced with suitable mechanically robust, thermally stable fillers.^[^ ^23^ ^]^ In particular, Li dendrite growth—induced by localized current formation and insufficient mechanical shielding—can be mitigated by uniform flux of Li⁺ through enhanced electrolyte wetting and puncture resistance. For instance, suppression of dendrite growth on Li‐metal anodes was evidenced by the smooth anode surface observed after 100 cycles when silica (SiO_2_) particles were applied to a microporous PE separator.^[^ ^24^ ^]^

Owing to the success of silicon oxides as fillers for battery separators, various other oxide materials such as TiO_2_,^[^ ^25^ ^]^ MgO,^[^ ^26^ ^]^ LiAlO_2_,^[^ ^27^ ^]^ ZrO_2_,^[^ ^28^ ^]^ BaTiO_3_,^[^ ^29^ ^]^ and Al_2_O_3_^[^ ^30^ ^]^ have also been studied. Although these oxide particles effectively enhance the durability of separators, their size must be deliberately controlled to ensure a wide, uniform pore‐size distribution;^[^ ^31^, ^32^ ^]^ otherwise, they may obstruct electrolyte passage via an increase in tortuosity, which cannot be mitigated by enhanced electrolyte wetting, causing the separator to exhibit poor ionic conductivity. Furthermore, since the advent of Li–S and Li metal batteries (LMBs), conventional separators have had to encounter new challenges such as hindering the migration of polysulfide intermediates (denoted the “shuttle” effect) and suppressing the growth of Li dendrites, both of which impede battery performance.^[^ ^33^ ^]^

Natural clay minerals, which possess diverse functional capabilities owing to their unique chemical compositions and structural features, can improve thermal stability, mechanical robustness, and electrolyte wettability.^[^ ^34^ ^]^ These minerals are abundant in nature and exhibit outstanding thermal and chemical stability, along with micro‐ and nanoporous structures that are desirable for achieving high ionic conductivity. In addition,^[^ ^34^ ^]^ the abundant surface‐level Lewis acid–base sites of natural minerals, such as Si–O, Al–O, and Mg–O bonds, serve as polar active sites to capture polysulfides through physical and chemical effects.^[^ ^34^, ^35^ ^]^ To harness these outstanding features of minerals, previous researchers reshaped clay minerals as µm‐thick thin films that are suitable for battery separators, by coating them on conventional ones. The resulting mineral‐coated separators exhibit excellent durability and ionic conductivity, making them promising alternatives to the commonly used polymer separators.^[^ ^36^ ^]^

Clay minerals also impart flame retardancy to polymeric materials via their high char‐forming capacity, as demonstrated by montmorillonite, vermiculite, boehmite, and halloysite.^[^ ^37^, ^38^, ^39^ ^]^ This effect can be further intensified by high‐aspect‐ratio layered structures in combination with char‐layer formation upon exposure to heat, thereby acting as an effective insulating barrier to heat transfer and gas release in highly flammable polymeric substances—for example, vermiculite‐coated polyurethane foam^[^ ^40^ ^]^ and cotton fabrics treated with montmorillonite and vermiculite.^[^ ^41^ ^]^

As exemplified by the two‐dimensional (2D) layered structures of montmorillonite and vermiculite in flame‐retardant applications, clay minerals offer unique, dimensionality‐dependent features that enhance separator performance. One‐dimensional (1D) tubular halloysite nanotubes can deliver Li ions through both the interior and exterior parts of the nanotubes, enhancing the ionic conductivity of the host separator.^[^ ^42^ ^]^ Such an increase in the ionic conductivity is particularly useful under low temperature conditions, as proved by the halloysite‐coated separators that allowed the improved cyclic stability of LIBs at a temperature near −20 °C.^[^ ^43^ ^]^ Moreover, zeolite endows superior mechanical strength to the host separator because of its three‐dimensional (3D) connected porous structure.^[^ ^44^ ^]^

From these perspectives, understanding the structural characteristics of minerals and their relationships with the separator performance is key to developing separators for future high‐performance LIBs. Clay minerals can be classified based on their atomic configuration or microstructure. From an atomistic perspective, clay minerals comprise octahedral layers with metal–oxygen bonds and tetrahedral layers with Si–O bonds, with the unit structure varying among different minerals. Clay minerals can also be categorized based on their morphology into 1D, 2D, and 3D configurations. However, despite the various classifications that have been proposed for clay minerals, the correlations between the type of mineral and the performance of mineral‐coated separators have not been comprehensively established. Therefore, the present review was aimed at plugging this knowledge gap. First, 14 representative clay minerals are classified based on their microstructural attributes into 1D, 2D, and 3D configurations. Subsequently, the characteristic features of each mineral, the performance of the resulting separators, and relevant previous studies are discussed. Additionally, to clarify the structure–performance relationships of the clay minerals, the separator parameters are summarized with respect to the type of coated mineral. Finally, perspectives are provided on maximizing the advantages of natural clay minerals to develop efficient high‐energy‐density LIBs with long‐term stability.

## 2 Types of Minerals for LIB Separators

### 2.1 Acicular/Fibrous (1D) Minerals

**Figure 1:**
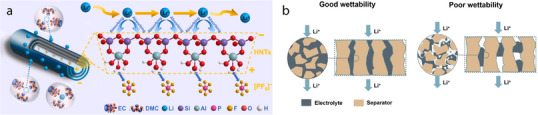
a) Halloysite nanotubes’ oppositely charged inner (+) and outer (–) walls create dual Li⁺ pathways through the lumen and along the exterior, boosting ionic conductivity. Reproduced with permission.^[^ ^62^ ^]^ Copyright 2025, Elsevier. b) Under good separator wettability, the electrolyte is fully absorbed, forming continuous Li⁺ channels; under poor wettability, electrolyte pockets fragment these pathways and impede ion transport. Reproduced with permission.^[^ ^63^ ^]^ Copyright 2025, Springer.

1D minerals comprise fibrous or tubular structures with a diameter of 5–50 nm and a length of 0.5–10 µm. These morphologies have often been reported for minerals from the kaolin and smectite groups, which are families of phyllosilicate minerals with layered structures. The kaolin group includes minerals such as halloysite and chrysotile, whereas the smectite group comprises attapulgite and sepiolite. The mechanism governing the formation of the 1D morphology varies depending on the type and structure of each mineral. For instance, kaolin minerals originally exhibit a planar configuration with a crystal unit of two layered sheets, that is, metal oxide octahedral sheets and Si–O tetrahedral sheets (referred to as a “1:1” layered structure). However, owing to the different charge states of the octahedral and tetrahedral sheets, the 1:1 layered structure curls into a tubular 1D configuration.^[^ ^42^ ^]^ Halloysite and chrysotile, which form such tubular morphologies, are representative kaolin minerals. In contrast, smectite minerals are characterized by a “2:1” layered structure in which a single metal oxide octahedral layer is sandwiched between two Si–O tetrahedral sheets. Some smectite minerals, including attapulgite and sepiolite, form small chain‐like structural units that laterally connect and aggregate into fibrous bundles, resulting in a 1D morphology.^[^ ^58^ ^]^ Owing to the differences in their atomic structures, kaolin and smectite minerals store and deliver Li ions differently. Specifically, kaolin minerals with tubular geometries, such as halloysite and chrysotile, are formed by the curling of their 1:1 layered structures due to electrostatic imbalance between the octahedral and tetrahedral sheets.^[^ ^42^ ^]^ These tubular structures possess chemically distinct inner and outer surfaces, resulting in a unique charge distribution that facilitates rapid Li‐ion transport through both the lumen and the exterior surface of the nanotubes.^[^ ^59^ ^]^ In contrast, smectite minerals with fibrous morphologies, such as attapulgite and sepiolite, form thin and densely packed fiber bundles with large pore volumes and high specific surface areas, resulting from their relatively small structural chain‐layer units.^[^ ^60^, ^61^ ^]^ While kaolin‐type minerals promote Li‐ion transport primarily through surface charge differences within their tubular structures (**Figure** **1**a), 1D smectite minerals enhance ionic conductivity by absorbing more liquid electrolyte into their porous structures, thereby facilitating ion migration through more abundant and continuous diffusion pathways (Figure 1b).

**Figure 2:**
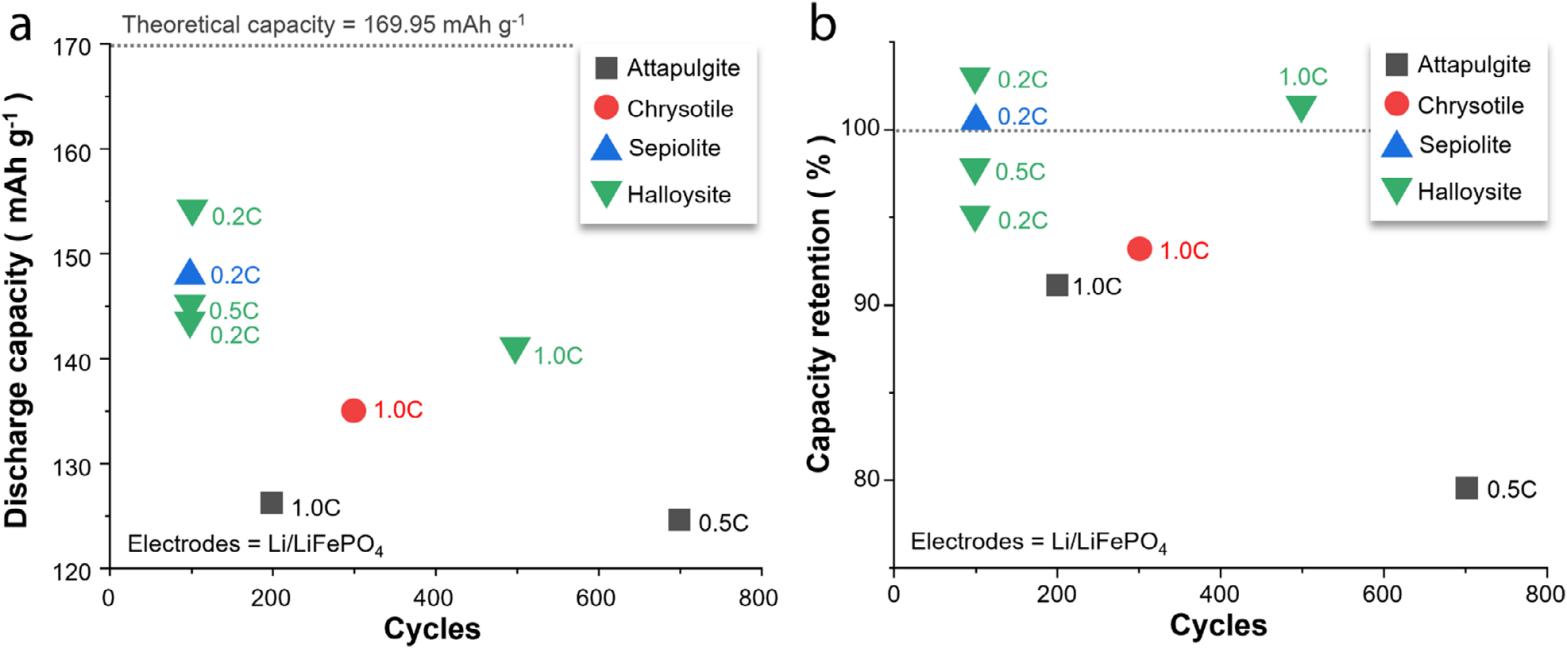
a) Discharge capacity–cycle number and b) capacity retention–cycle number plots for Li/LiFePO_4_ batteries with separators composed of acicular/fibrous minerals, with the C‐rate for each battery test shown next to the symbols.^[^ ^45^, ^46^, ^47^, ^49^, ^52^, ^53^, ^54^, ^55^ ^]^

In addition to augmenting Li‐ion conductivity, 1D minerals impart superior mechanical properties and high thermal stability to separators, causing them to outperform conventional PP‐ and PE‐based separators. To leverage these features, researchers have coated 1D minerals onto various LIB separators (**Table** **1**). Although different coating techniques and host separators have been used, the overall electrochemical performance has been enhanced by applying 1D minerals to batteries, including LIBs, Li–S batteries, and symmetric cells. Among the various battery systems, LMBs with Li metal anodes and LiFePO_4_ cathodes have been frequently reported for testing the performance of 1D‐mineral‐coated separators. Therefore, the Li/LiFePO_4_ battery was selected as a representative system in this review to compare the electrochemical performance of the 1D‐mineral‐based separators (**Figure** **2**).

**Table 1:** Performance of composite separators fabricated using acicular/fibrous minerals.

Mineral	Host separator	Method	Electrode materials	Liquid electrolyte	Capacity retention (number of cycles)	C‐rate	Reference
Attapulgite	Celgard@2400	Deposition	Li, LiFePO_4_	LiPF_6_, 1:1 EC/DMC	100.3% (200)	1.0C	[45]
	SA	Phase inversion	Li, LiFePO_4_	LiPF_6_, 1:1 EC/DEC	82.20% (700)	0.5C	[46]
Chrysotile	‐	Vacuum filtration	Li, LiFePO_4_	LiPF_6_, 1:1:1 EC/EMC/DEC	100.0% (300)	1.0C	[47]
Sepiolite	PE	Coating	Li, LiCoO_2_	LiPF_6_, 1:1:1 EC/DMC/EMC	84.50% (100)	0.5C	[48]
	PU	Electrospinning	Li, LiFePO_4_	DMF	100% (100)	0.2C	[49]
	PP	Electrospinning	Li, LiNi_0.5_Mn_1.5_O_4_	LiPF_6_, 1:1:1 EC/EMC/DEC	88.90% (200)	1.0C	[50]
Halloysite	PP	Coating	Li, LiCoO_2_	LiPF_6_, 1:1:1 EC/DMC/DEC	73.13% (300)	1.0C	[51]
	A4 paper	Coating	Li, LiFePO_4_	LiPF_6_, 1:1:1 EC/DMC/DEC	97.60% (100)	0.5C	[52]
	BC	Vacuum filtration	Li, LiFePO_4_	LiPF_6_, 1:1:1 EC/DMC/DEC	95% (100)	0.2C	[53]
	PVA	Coating	Li, LiFePO_4_	LiPF_6_, 1:1 EC/DEC	100% (100)	0.2C	[54]
	PE	Coating	Li, LiFePO_4_	LiPF_6_, 3:7 EC/DEC	103.70% (500)	1.0C	[55]
	PVA	Electrospinning	Li, Anthraquinone	LiTFSI (1 M), 1:1 DOL/DME	91.40% (300)	0.2C	[56]
	Celgard@3501	Coating	Li, Sulfur multiwalled carbon nanotube	LiTFSI (1 M) + LiNO_3_ (0.1 M), 1:1 DOL/DME	50.00% (100)	0.05C	[57]
	SPEEK	Coating	Graphite felt, graphite felt	DMF	50.00% (500)	80 mA cm^−2^	[43]

Li/LiFePO_4_ batteries with halloysite‐ or sepiolite‐coated separators have exhibited higher discharge capacities than those of counterparts with chrysotile‐ or attapulgite‐coated separators during the last battery cycle (Figure 2a). A similar trend has been observed for capacity retention (Figure 2b). Notably, certain batteries fabricated using halloysite or sepiolite have demonstrated capacity retention values exceeding 100%, indicating that the charge–discharge capacity was recovered as the cycling proceeded. This suggests that coating halloysite or sepiolite onto the separator can significantly extend the battery cycle life while preserving a moderate energy density. To elucidate the potential origins of the performance of 1D‐mineral‐coated Li‐ion separators, the atomic configurations/microstructures of the 1D minerals and the battery‐testing conditions are explored in the sections below.

#### 2.1.1 Attapulgite (Palygorskite)

**Figure 3:**
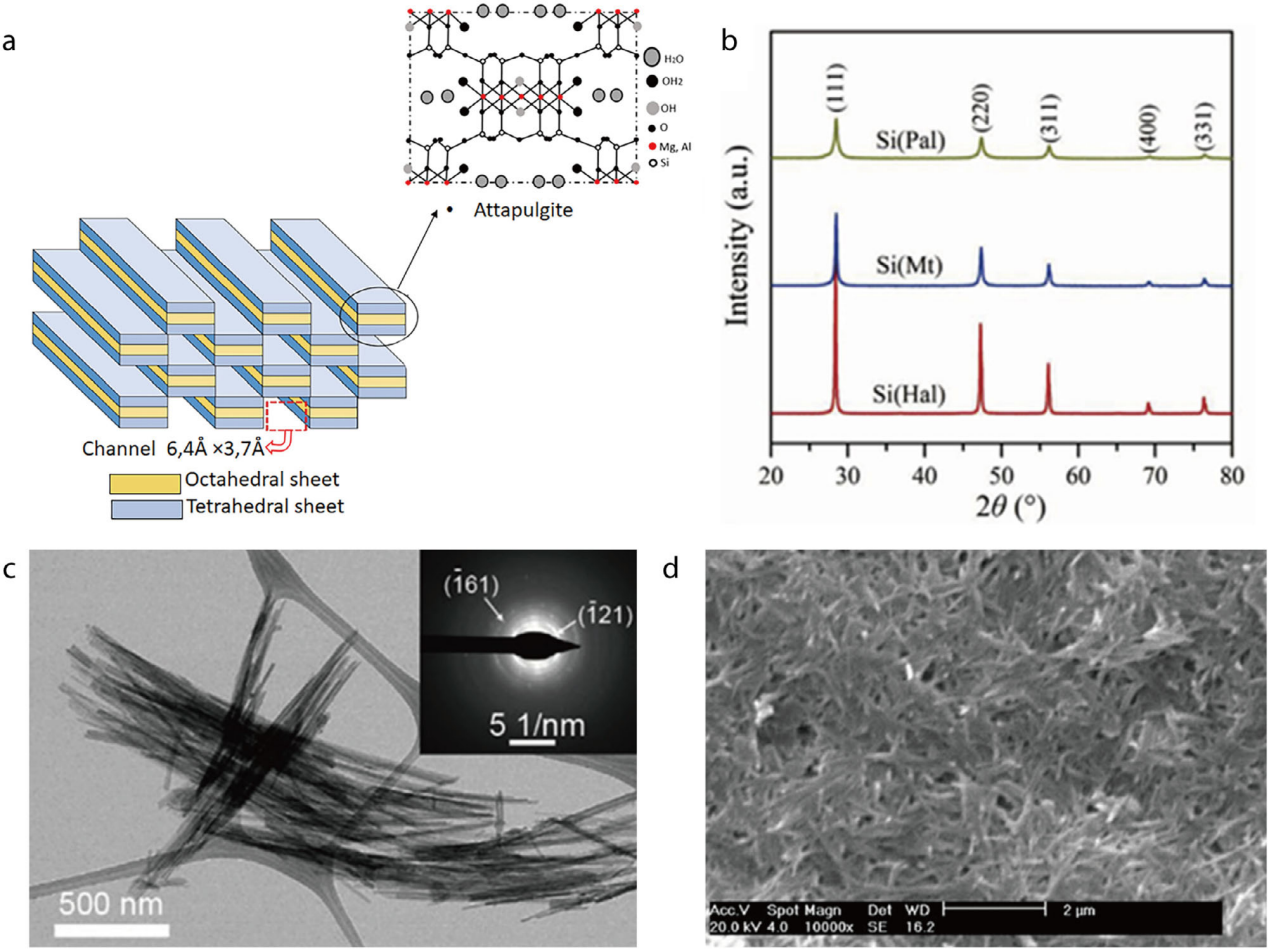
a) Crystal structure of ATP. Reproduced with permission.^[^ ^65^ ^]^ Copyright 2023, Wiley‐VCH. b) XRD pattern of ATP. Reproduced with permission.^[^ ^66^ ^]^ Copyright 2018, Elsevier. c) TEM image of ATP fibers. Reproduced with permission.^[^ ^46^ ^]^ Copyright 2019, Elsevier. d) SEM micrograph of ATP fibers. Reproduced with permission.^[^ ^67^ ^]^ Copyright 2005, Elsevier.

Attapulgite (ATP; also called palygorskite) is a natural hydrated magnesium aluminum silicate clay mineral with the chemical formula (Al_2_Mg_2_)Si_8_O_20_(OH_2_)_4_•4H_2_O).^[^ ^45^, ^64^ ^]^ It exhibits a layered chain structure featuring a continuous Si–O tetrahedral layer and a discontinuous Mg(Al)–OH/O octahedral layer (**Figure** **3**a),^[^ ^65^ ^]^ resulting in a unique nanorod‐like microstructure with interconnected micro/nanopores. In its x‐ray diffraction (XRD) pattern, ATP exhibits characteristic peaks at 2*θ* values of 8.34°, 27.49°, 35.0°, and 42.6°, which correspond to the (110), (200), (040), and (400) crystal planes, respectively (Figure 3b).^[^ ^66^ ^]^ According to transmission electron microscopy (TEM) imaging, ATP exists as ≈1‐µm‐long nanofibers with a diameter of ≈50 nm (Figure 3c).^[^ ^46^ ^]^ Additionally, scanning electron microscopy (SEM) imaging has revealed the fibrous morphology of ATP (Figure 3d).^[^ ^67^ ^]^ Notably, ATP can absorb a large amount of liquid owing to its porous fiber bundles; thus, it is extensively used as an agricultural carrier and environmental absorbent. Furthermore, ATP can readily form interconnected percolating networks even at low concentrations, providing excellent mechanical properties.^[^ ^68^ ^]^

**Figure 4:**
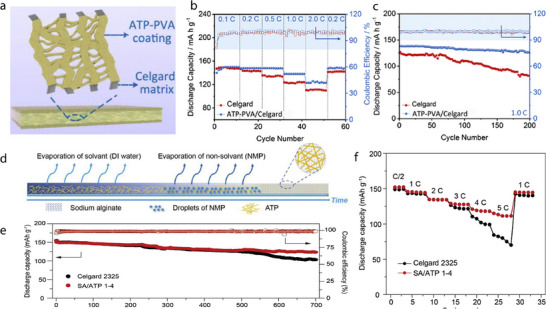
a) Schematic of ATP–PVA/Celgard separator with a sandwiched/infused structure. b, c) Electrochemical performance of Li/LiFePO_4_ cell with ATP–PVA/Celgard separator: b) rate performance and c) cycling stability at 1.0C (160 mA g^−1^). Reproduced with permission.^[^ ^45^ ^]^ Copyright 2020, Elsevier. d) Schematic illustrating mechanism underlying phase inversion. e) Long‐term cycling performance of LiFePO_4_/Li cells with Celgard2325 or SA/ATP 1‐4 separators at 0.5C. f) Rate performance of LiFePO_4_/Li cells with Celgard or SA/ATP 1‐4 separators at various C‐rates. Reproduced with permission.^[^ ^46^ ^]^ Copyright 2019, Elsevier.

Yang et al. prepared composite separators by depositing ATP and polyvinyl alcohol (PVA) coatings on both the inner and outer surfaces of a microporous PP membrane (Celgard 2400) (**Figure** **4**a).^[^ ^45^ ^]^ The separator was produced using a simple dip‐coating process in which the wet Celgard separator was immersed in an ATP–PVA suspension for 20 s and withdrawn at a speed of 20 mm s^−1^; the process was repeated twice to obtain a uniform ATP–PVA coating. The ATP–PVA/Celgard separator was slightly more porous than the Celgard separator owing to the abundance of micro/nanosized channels interconnected to the ATP nanorods, thereby improving the Li^+^ conductivity of the Celgard separator. Moreover, the ATP–PVA/Celgard separator exhibited a higher affinity for electrolytes, higher thermal resistance, and greater mechanical flexibility than those of the Celgard separator. The effects of the ATP–PVA/Celgard separator on LIB performance were studied using a Li/LiFePO_4_ cell. At 2.0C, the reversible capacity of the cell with the ATP–PVA/Celgard separator was 123 mAh g^−1^, and when the rate was switched to 0.2C, the separator maintained a high reversible capacity of 148.5 mAh g^−1^ (Figure 4b). Additionally, the ATP–PVA/Celgard separator significantly improved the cycling stability of the cell. At 1.0C, the capacity of the cell with the Celgard separator decreased sharply after 70 cycles; in contrast, the capacity retention rate of the cell with the ATP–PVA/Celgard separator was as high as 91.7% (Figure 4c).

Song et al. prepared a composite separator membrane using ATP nanofibers and sodium alginate (SA) as the additive and substrate, respectively, by phase inversion.^[^ ^46^ ^]^ Specifically, the polymer component was dissolved in a mixture of water and N‐methyl‐2‐pyrrolidone (NMP), followed by sequential evaporation of water and NMP, which yielded a porous ATP‐based membrane (Figure 4d). Besides phase inversion, other typical separator fabrication methods include electrospinning and mechanical stretching, which are widely used for preparing fibrous or polyolefin‐based membranes, respectively. The ATP‐coated SA separator (SA/ATP) showed improved thermal stability, outperforming the Al_2_O_3_‐coated PE separator and Li‐lanthanum‐titanate‐coated PE separator, which severely curled and shrank at elevated temperatures. In this context, while high temperature has been emphasized as a primary cause of separator failure, other harsh operating conditions such as external mechanical stress and high humidity can also significantly impact separator performance by accelerating pore collapse, delamination, or loss of dimensional stability. In general, conventional PE/PP separators tend to shrink or melt at temperatures above 120–140 °C, which is why many coated separators are tested for thermal resistance at or above this threshold. Furthermore, the SA/ATP membrane exhibited excellent electrolyte wettability owing to its numerous sub‐5‐nm‐sized pores. The LiFePO_4_/Li cell with the SA/ATP separator exhibited a higher specific capacity than that of the cell with the Celgard separator (152 and 148.9 mAh g^−1^, respectively) (Figure 4e). Moreover, it showed a considerably higher capacity retention rate after 500 cycles at 0.5C than that of the cell with Celgard 2325 (82.2% and 68.7%, respectively) (Figure 4f). The superior electrochemical performance of the SA/ATP separator, combined with its mechanical strength, flame retardancy, and thermal stability, imparted several advantageous properties to the battery.

#### 2.1.2 Chrysotile

**Figure 5:**
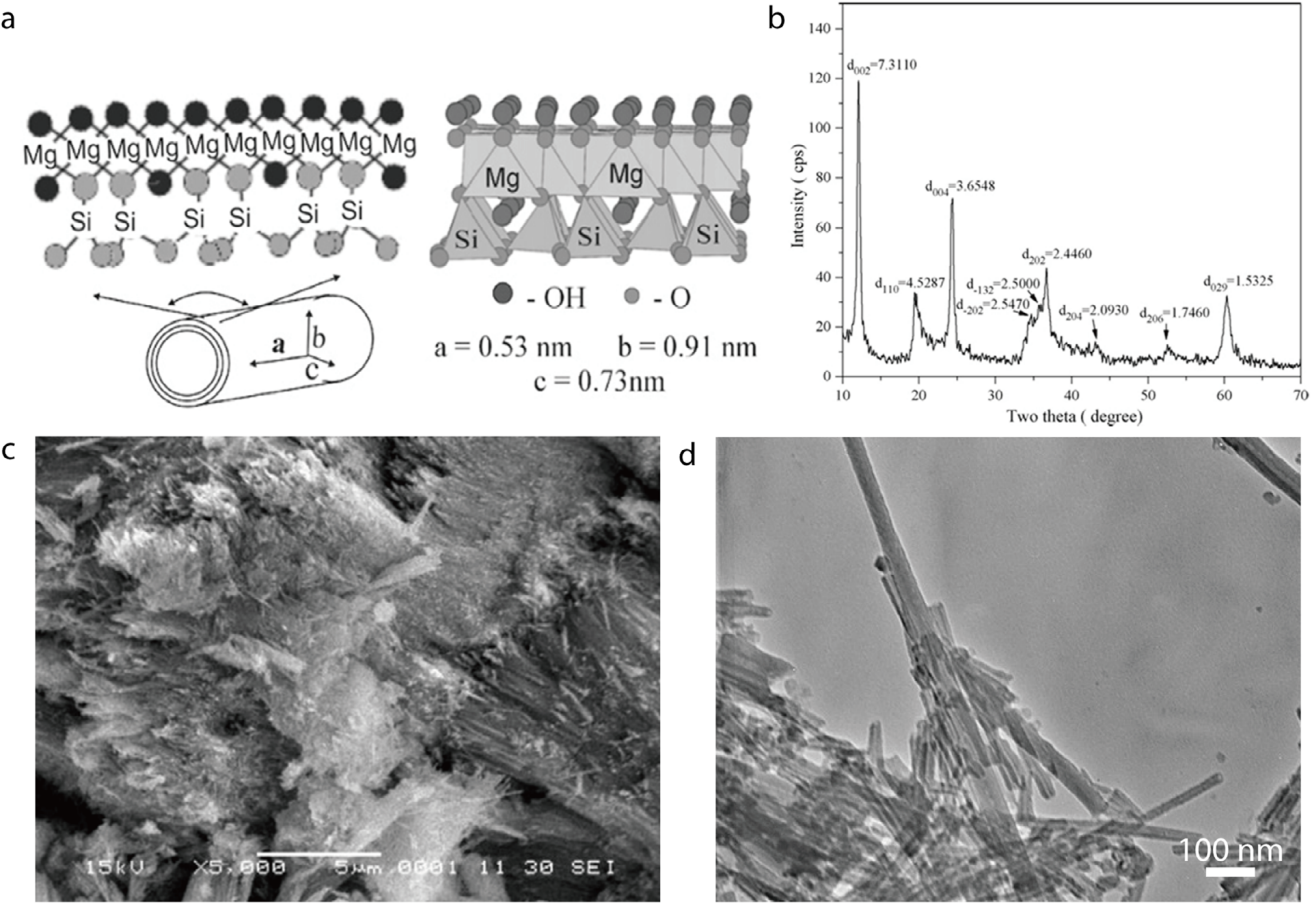
Characteristics of chrysolite: a) Crystalline structure. Reproduced with permission.^[^ ^69^ ^]^ Copyright 2011, Elsevier. b) Powder XRD pattern. c) SEM image Reproduced with permission.^[^ ^70^ ^]^ Copyright 2010, Elsevier. and d) TEM image Reproduced with permission.^[^ ^71^ ^]^ Copyright 2012, Wiley‐VCH.

Chrysotile (Mg_3_Si_2_O_5_(OH)_4_) is a fibrous hydrated magnesium silicate mineral comprising inner octahedral sheets of brucite (Mg(OH)_2_) covalently bonded to outer tetrahedral sheets of tridymite (SiO_4_) (**Figure** **5**a).^[^ ^69^ ^]^ In its XRD pattern, chrysotile exhibits five major reflections from the (002), (110), (004), (202), and (029) crystal planes, which correspond to d‐spacing values of 7.3110, 4.5287, 3.6548, 2.4460, and 1.5325 Å, respectively (Figure 5b).^[^ ^70^ ^]^ The strongest reflection peak emerges from the (002) plane, which is perpendicular to the octahedral and tetrahedral sheets, indicating the “1:1” layered structure of chrysotile. However, on a microscopic scale, chrysotile comprises bundles of parallel fibers, with each fiber elongated parallel to one crystallographic direction (Figure 5c).^[^ ^70^ ^]^ The 1:1 layered structure of chrysotile becomes coiled, forming elongated nanotubes with an average diameter of 42.03 nm and an average length of 1.58 µm, as revealed by TEM (Figure 5d).^[^ ^71^ ^]^ Owing to its durability, heat resistance, tensile strength, and electrical resistance, chrysotile is widely used in areas such as thermal insulation, catalysis, filtration, and the design of friction materials for brakes.

Chrysotile fibers (CFs) have been used as separators without a host matrix for LIBs owing to their high ionic conductivity, thermal stability, and excellent electrolyte wettability. For example, Cai et al. prepared CF separators by simple vacuum filtration.^[^ ^47^ ^]^ Cross‐sectional SEM imaging revealed that the CF separator membrane was thicker than the PP separator membrane (≈68 and 28 µm, respectively). Additionally, SEM imaging of the surface of the CF separator revealed a random arrangement of ≈100‐nm‐diameter nanofibers. Owing to its high porosity, favorable affinity for the electrolyte, and unique sandwich‐like structure, the CF separator outperformed its PP counterpart in terms of wettability. The electrochemical performance was evaluated using LiFePO_4_/separator/Li cells assembled with CF or commercial PP as the separator. At a high current rate of 5C, the initial discharge capacity of the cell with the CF separator was notably higher than that of the cell with the PP separator (111 and 88 mAh g^−1^, respectively). Furthermore, the CF separator was more thermally resistant than the conventional PP separator. Specifically, upon exposure to a high temperature of 120 °C for 2 min, the LiFePO_4_/separator/Li cell assembled with the PP separator showed a substantial decrease in specific capacity up to 1 mAh g^−1^. In contrast, even after heating at 120 °C for 20 min, the cell with the CF separator showed an almost constant specific capacity, indicating that the heat treatment did not affect its electrochemical performance. This study demonstrated the superior thermal resistance of the CF separator and its contribution to the stable operation of the cell at high temperatures.

**Figure 6:**
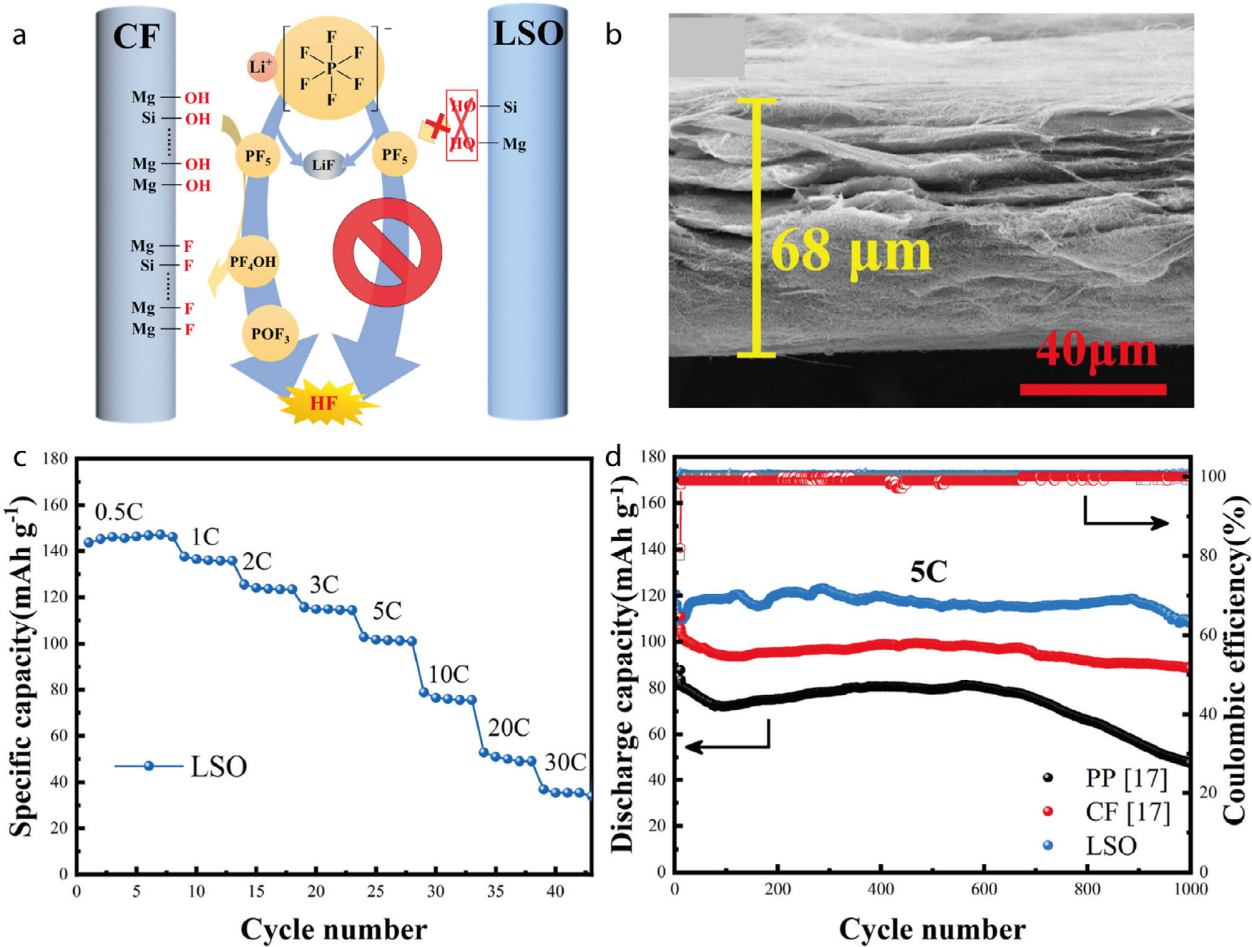
a) The promoting effect of the hydroxyls on the decomposition of electrolyte. b) Cross‐sectional SEM image of LSO separator. c) Rate performance of LiFePO_4_/separator/Li cells with LSO separator from 0.5C to 30C. d) Charge‐discharge profiles at 1C of the LiFePO_4_/separator/Li cells assembled with LSO. Reproduced with permission.^[^ ^72^ ^]^ Copyright 2022, Elsevier.

In a related study, Kang et al. systematically investigated the root cause of reduced initial coulombic efficiency in LIBs with CF‐based separators and proposed an effective surface modification strategy to address this challenge. The abundant hydroxyl groups on the CF surface, specifically Si–OH and Mg–OH, react with PF_5_ generated from the decomposition of LiPF_6_, forming SiF/MgF and PF_4_OH, which are further converted to hydrofluoric acid (HF) and POF_3_, accelerating electrolyte solvent degradation (**Figure** **6**a).^[^ ^72^ ^]^ These side reactions lead to performance deterioration and a significant decrease in initial coulombic efficiency, highlighting the importance of limiting surface hydroxyl groups. To achieve this, the authors employed a two‐step acid leaching process followed by Li_2_SO_4_ treatment to selectively scavenge hydroxyl groups from the CF surface, resulting in the modified CF (LSO). XRD analysis confirmed that the primary crystal structure of CF remained intact after modification, while a SEM image revealed that LSO retained the same three‐dimensional network architecture but exhibited a rougher surface and the formation of surface particles, resulting in a slight decrease in porosity (Figure 6b). However, this structural change contributed to improved electrolyte wettability and infiltration, enhancing interfacial stability. Electrochemical performance tests showed that LiFePO_4_/Li cells assembled with LSO achieved excellent rate capabilities of 144, 138, 126, 116, and 103 mAh g^−1^ at 0.5, 1, 2, 3, and 5C, respectively (Figure 6c). Even under high‐rate cycling at 5C, the initial discharge capacity of 120.6 mAh g^−1^ was well maintained, with a capacity of 109.4 mAh g^−1^ retained after 1000 cycles, corresponding to a capacity retention of 90.7% (Figure 6d). This clearly demonstrates the superior performance of the modified CF separator compared to pristine CF and conventional PP separators.

#### 2.1.3 Sepiolite

**Figure 7:**
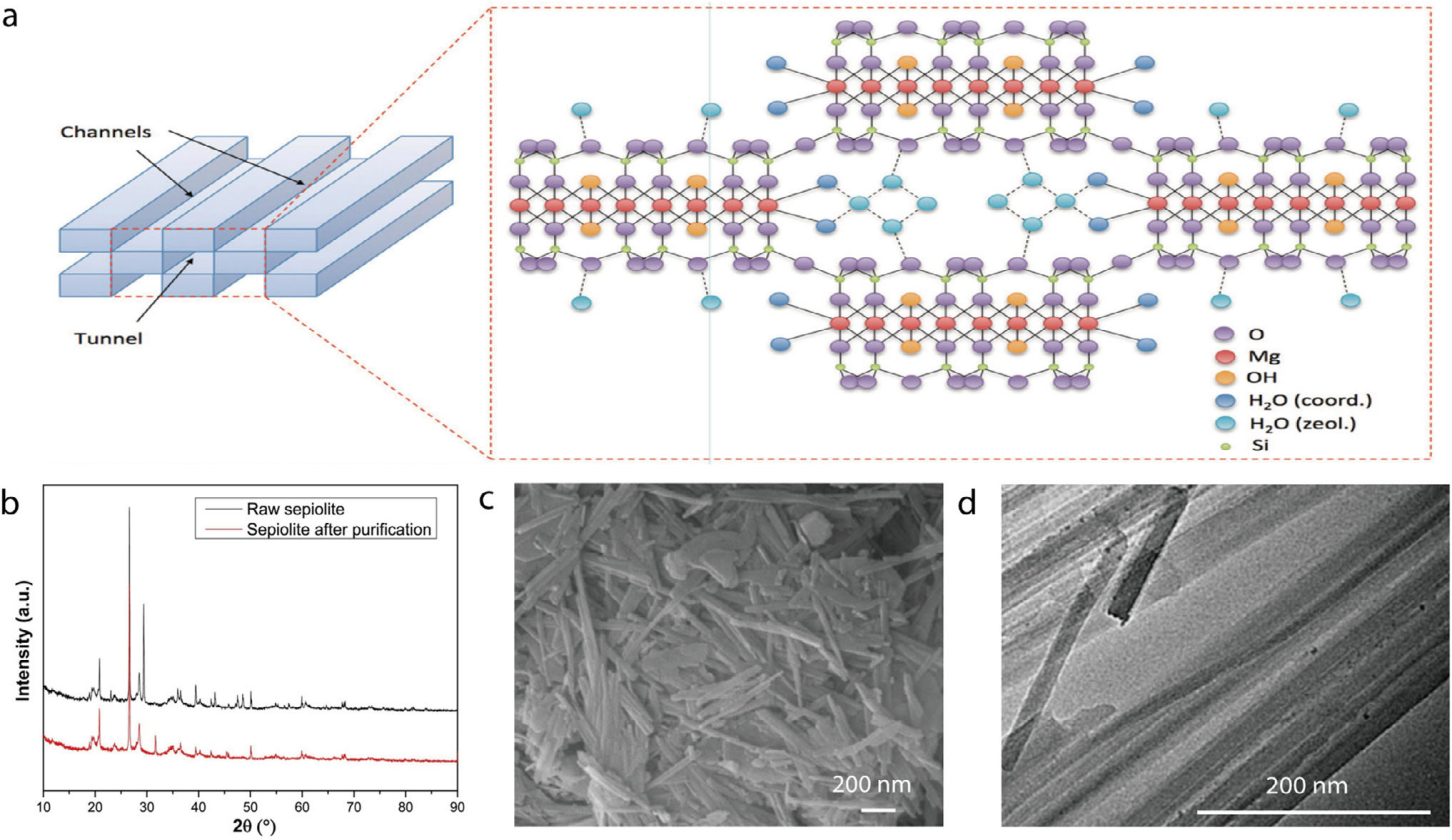
a) Crystal structure of sepiolite. Reproduced with permission.^[^ ^73^ ^]^ Copyright 2018, Wiley‐VCH. b) XRD patterns of sepiolite before and after purification. c) SEM image of sepiolite after purification. d) TEM image of sepiolite after purification. Reproduced with permission.^[^ ^49^ ^]^ Copyright 2019, Elsevier.

Sepiolite is commonly obtained from European countries such as Turkey and Spain or from China. It is a natural hydrated magnesium silicate with the theoretical unit‐cell formula of (Si_12_Mg_8_O_30_)(OH)_4_(OH_2_)_4_•8H_2_O. The unit cell comprises two sheets of tetrahedral and single octahedral oxides, which form a 2:1 layered structure. Evidently, sepiolite exists mostly in the form of fibrous or fiber bundles containing fine tubular channels with a cross‐section size of 0.37 × 1.06 nm, which can accommodate water and other molecules (**Figure** **7**a).^[^ ^73^ ^]^ Sepiolite crystals have a natural pore structure between the fiber bundles, which bestows the crystals with a strong ability to absorb liquid electrolytes.^[^ ^48^ ^]^ Typically, natural sepiolite clay requires purification before it can be used to fabricate battery separators because it contains various impurities such as quartz, calcite, and talc. The changes in the phase fractions of the sepiolite clays during purification have been examined by comparing their XRD patterns. For instance, XRD patterns have shown a significant decrease in the reflection intensities of quartz (2*θ* = 19°, 24.2°, and 44.9°) and calcite (2*θ* = 26.6° and 35.5°) after purification (Figure 7b).^[^ ^49^ ^]^ In other words, the purification not only removes impurities but also alters the microscopic structures, with raw sepiolite powders exhibiting a closely packed nanofibrous configuration and purified sepiolite containing agglomerated bundles of fibrous structures with sizes ranging from hundreds of nanometers to several micrometers (Figure 7c).^[^ ^49^ ^]^ TEM imaging has clearly revealed fibrous structures with an average diameter of ≈30 nm for purified sepiolite (Figure 7d).^[^ ^49^ ^]^

**Figure 8:**
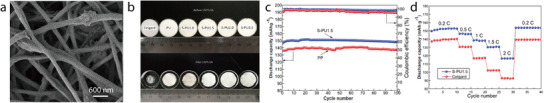
a) SEM image of S‐PU2.0 separator (sepiolite/PU mass ratio = 2.0:1). b) Photographs of Celgard and composite separators before and after heating in an oven at 150 °C for 1 h. c) Cycling performance at a charge/discharge rate of 0.2C. d) Rate capability. Reproduced with permission.^[^ ^49^ ^]^ Copyright 2019, Elsevier.

Deng et al. fabricated separators composed of polyurethane (PU) and purified sepiolite nanofibers via electrospinning (**Figure** **8**a).^[^ ^49^ ^]^ The precursor solution for electrospinning was prepared by dispersing purified sepiolite into the PU solution at different sepiolite/PU mass ratios (1:1, 1.5:1, 2.0:1, and 2.5:1). As the amount of sepiolite in the precursor solution increased, the diameter of the sepiolite fibers decreased owing to the decrease in the solution viscosity. The increase in the sepiolite content also improved the mechanical properties, as indicated by the superior elastic modulus of the composite separators with high sepiolite content. Additionally, the sepiolite‐based separators maintained their shape after being heated in an oven at 150 °C for 1 h, signifying their good thermal stability, which was ascribed to the role of the sepiolite nanofibers as a thermostable framework; in contrast, the Celgard separator exhibited 78% shrinkage (Figure 8b). To assess the effect of sepiolite on separator performance, cycle and rate tests were conducted using cells with LiFePO_4_ as the cathode and Li metal as the anode. Among the synthesized sepiolite‐based separators, the specimen with a sepiolite/PU mass ratio of 1.5:1 (S‐PU1.5) exhibited a higher discharge capacity than that of the Celgard separator, while exhibiting a remarkably high coulombic efficiency of ≈100% in the first 100 cycles (Figure 8c). In terms of rate performance, the cell with the S‐PU1.5 separator achieved a higher capacity at 2.0C than that of the cell with the Celgard separator (120 and 92 mAh g^−1^, respectively). Additionally, when the current rate was reduced back to 0.2C, the cell with the S‐PU1.5 separator recovered most of its capacity (Figure 8d). Furthermore, upon being cycled at 60 °C, the cell with the S‐PU1.5 separator exhibited a high specific discharge capacity of 142 and 125 mAh g^−1^ at the 1st and 50th cycles, respectively, leading to a capacity retention of 88%; in contrast, the cell with the Celgard separator showed capacity fluctuations.

Guo et al. prepared a sepiolite/PP (S‐PP) composite separator via electrospinning for high‐voltage LiNi_0.5_Mn_1.5_O_4_ (LNMO) batteries.^[^ ^50^ ^]^ This study highlighted another important aspect of sepiolite‐based separators, that is, their enhanced structural tolerance at high voltages, which was quantified by the electrochemical oxidation limit. Specifically, the sepiolite particles in the PP separator increased the electrochemical oxidation limit, enabling use at high voltages without damage. This behavior originated from several beneficial features of sepiolite, such as the stabilization effect, strong affinity for the electrolyte, and ability to absorb impurity molecules in the electrolyte. Furthermore, the S‐PP separator exhibited less thermal shrinkage and high ionic conductivity. Additionally, the LNMO/Li battery separator produced by coating sepiolite on the side facing the Li anode exhibited better long‐term cyclability than that of the specimen prepared by coating both sides of the separator. This improvement was attributed to the formation of a stable solid electrolyte interphase (SEI) layer on the Li anode by sepiolite, which played a crucial role in enhancing the cycle life by consuming the HF produced during electrolyte decomposition. In contrast, when the sepiolite layer faced the LNMO cathode, it effectively protected the LNMO cathode from HF corrosion but also inevitably led to the formation of Li dendrites on the Li anode, resulting in a capacity performance similar to that of the cell with the PP separator.

Zhang et al. intentionally removed the tetrahedral sheets of sepiolite to obtain a 1D structure with only Si and O. This was achieved by microwave‐assisted acid activation, which resulted in uniformly coated silicon nanorods (SNRs). The acid activation treatment, which aided in removing impurities and magnesium from the sepiolite skeleton and opening the internal channels of the crystal, induced the sepiolite to change from a fiber structure to a rod‐like configuration. Moreover, the rich 3D pore structure of the SNR coating enhanced the dimensional stability of the film at high temperatures. To verify the superior performance of the SNR‐coated separators, the SNRs were coated on both sides of a PE substrate using an organic–inorganic composite binder (PVA–phosphate inorganic binder; PVA–PIB).^[^ ^48^ ^]^ The PVA organic binder anchored the SNR coating to the surface of the PE substrate to prevent shedding, whereas the PIB maintained the integrity of the SNR coating at temperatures above 200 °C. The SNR coating with the PVA–PIB organic–inorganic composite binder protected the membrane from thermal shrinkage even at a high temperature of 200 °C. Additionally, the issue of low peel strength, which is a common problem with coatings based on inorganic binders, was mitigated. The SNR coating exhibited excellent electrolyte uptake (212.5%) owing to its high porosity (36.9%) and strong absorbability, which promoted the diffusion of the electrolyte through capillary forces. Consequently, the Li‐ion conductivity increased to 1.38 mS cm^−1^. Furthermore, the LiCoO_2_/Li battery integrated with the SNR‐based separator exhibited improved electrochemical performance with high capacity retention (84.5%), stable cycle retention, and high rate performance.

#### 2.1.4 Halloysite

**Figure 9:**
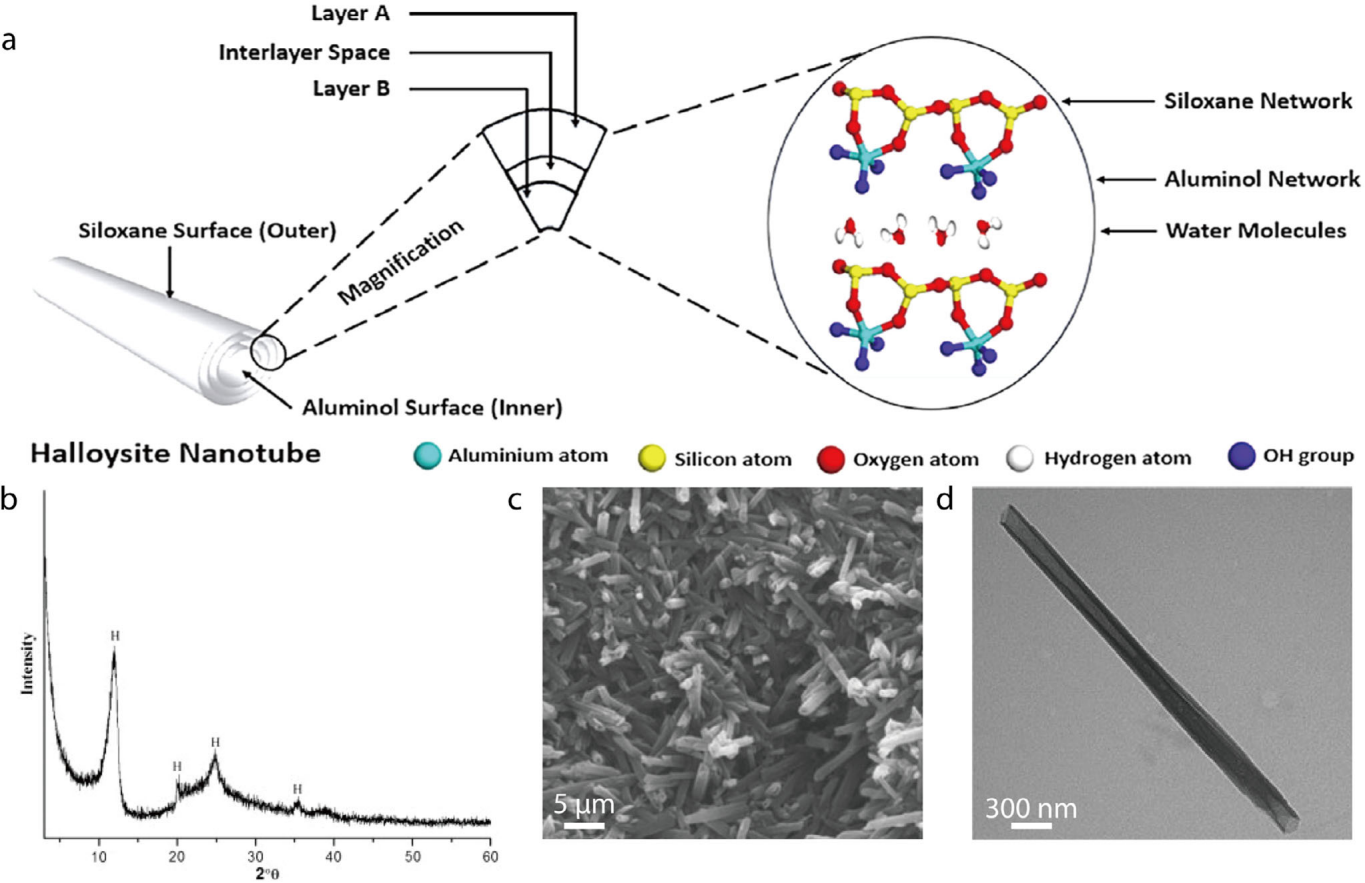
a) Structure of HNTs. Reproduced with permission.^[^ ^75^ ^]^ Copyright 2024, Wiley‐VCH. b) X‐ray diffraction pattern of halloysite Nanoclay. Reproduced with permission^[^ ^77^ ^]^ Copyright 2010, Elsevier. c) SEM micrograph of raw HNT powders. d) TEM micrograph of raw HNT powders. Reproduced with permission.^[^ ^78^ ^]^ Copyright 2016, Wiley‐VCH.

Halloysite nanotubes (HNTs; Al_2_Si_2_O_5_(OH)_4_•2H_2_O)) are hydrated layered aluminosilicates that belong to the kaolin group and exhibit a 1:1 layered structure comprising Al–O octahedral sheets and Si–O tetrahedral sheets.^[^ ^52^, ^74^ ^]^ The 1:1 layered structures of the HNTs curl up to achieve a tubular configuration, similar to that of chrysotile, which allows them to contain additional water molecules between the alumina and silicate layers (**Figure** **9**a).^[^ ^75^ ^]^ This halloysite wrapping arises from the oppositely charged surfaces of the 1:1 layered structures, that is, the internal and external surfaces comprising gibbsite (Al‐OH) groups and siloxane (Si–O–Si) groups, respectively.^[^ ^52^ ^]^ Consequently, the internal surface is positively charged, and the outer surface is negatively charged, leading to halloysite wrapping and HNT formation.^[^ ^76^ ^]^ In their XRD patterns, HNTs exhibit three characteristic reflection peaks at 2*θ* values of 12.1°, 20.1°, and 24.7°, which correspond to the (001), (110), and (002) crystal planes, respectively (Figure 9b).^[^ ^77^ ^]^ The well‐defined tubular structure of native HNTs has been confirmed by both SEM and TEM (Figure 9c,d).^[^ ^78^ ^]^ HNTs typically have a length of 200–2000 nm, an inner diameter of 10–40 nm, and an outer diameter of 40–70 nm.^[^ ^43^, ^79^ ^]^ HNTs have attracted widespread attention owing to their advantages such as a high aspect ratio, presence of hydroxyl groups on the surface, good thermal stability, biocompatibility, environmental friendliness, and low‐cost synthesis.^[^ ^76^, ^79^ ^]^

Halloysite is a highly popular mineral for coating battery separators; consequently, numerous studies have been conducted in this regard. For instance, Xie et al. prepared HNTs@PP separators by coating HNTs onto both sides of a PP separator.^[^ ^51^ ^]^ The coating layer created a 3D space that expanded the pores on the membrane surface, thereby increasing the capacity for electrolyte adsorption. Consequently, the HNTs@PP separators exhibited superior wettability, electrolyte uptake (224.6%), and ionic conductivity (0.66 mS cm^−1^). In terms of thermal stability, the bare PP separator completely melted at 170 °C, whereas the HNTs@PP separator contracted only by 7.9% at 180 °C. Notably, the bare and composite separators did not differ in terms of tensile strength. Additionally, the HNTs in the battery separator significantly improved the cycling stability of an LIB because the Al_2_O_3_ in the HNTs reacted with LiPF_6_ in the electrolyte to form the fast, ion‐conducting Li salt LiPO_2_F_2_. Consequently, the composite separator with the HNT coating did not show any changes in thickness upon cycling, whereas the PP separator accumulated a layer of dead Li near the Li side, hindering the movement of Li ions and adversely affecting the cycling performance. Therefore, after 300 cycles at 1C, the cell with the HNT‐coated composite separator exhibited a capacity retention rate that was 44.42% higher than that of the cell with the bare PP separator. Additionally, the cell with the composite separator exhibited high specific discharge capacities at rates of 0.5C, 1C, 2C, and 3C.

**Figure 10:**
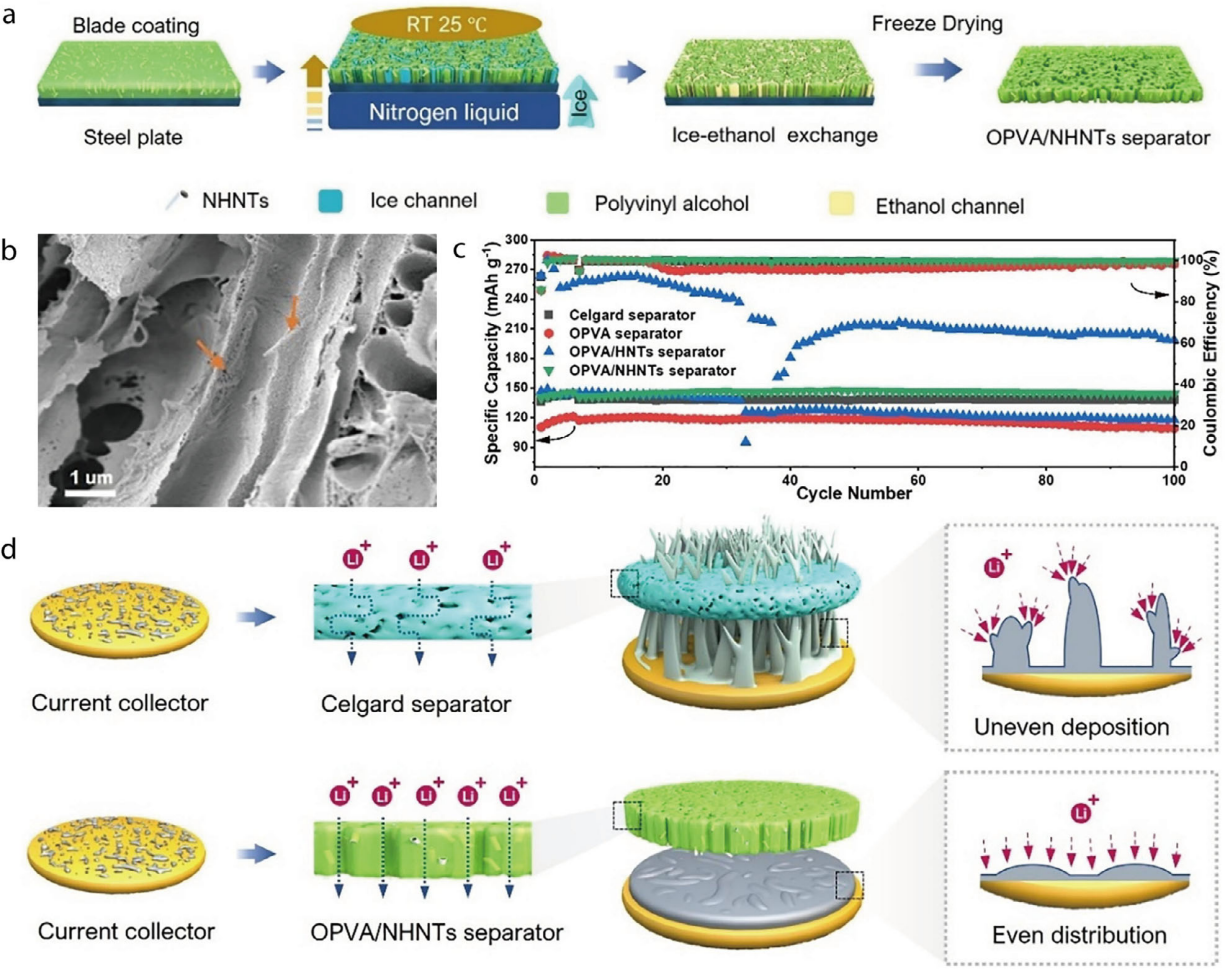
a) Schematic illustrating preparation of PVA/NHNT separator. b) SEM image of cross‐section morphology of the OPVA/NHNTs separator. c) Cycling performance of cells containing a separator fabricated using Celgard, pristine oriented PVA (OPVA), OPVA/HNTs, or OPVA/NHNT separators at C/5 with cutoff voltages of 2.8–4.2 V for 100 cycles. d) Schematic illustration of the growth of Li dendrites and long‐term cycling performance. Schematic image of Li dendrites for batteries with commercial Celgard separator and OPVA/NHNTs separator. Reproduced with permission.^[^ ^54^ ^]^ Copyright 2023, Elsevier.

In addition to commercial microporous PP membranes, other types of polymer membranes have been used as substrates for HNT coating. For example, Wang et al. synthesized nano‐architectured HNTs (NHNTs) and dispersed them in an oriented PVA (OPVA) system to prepare an OPVA/NHNT solution, which was then cast onto 200‐µm‐thick steel plates by doctor blade coating—a simple, viable, and cost‐effective method that is conducive to mass production (**Figure** **10**a).^[^ ^54^ ^]^ The membrane incorporated with the NHNTs exhibited a higher ionic conductivity than that of the Celgard and OPVA/HNT membranes. Additionally, the OPVA/NHNT membrane exhibited high porosity and good electrolyte compatibility. A cross‐sectional SEM image further confirmed that numerous NHNTs were well dispersed on the inner surface of the tubular pores (Figure 10b). This uniform distribution provides a large internal surface area, which facilitates strong electrolyte affinity and promotes efficient ion transport within the separator. Moreover, unlike the commercial Celgard separator, which showed pronounced thermal shrinkage at temperatures above 140 °C, the OPVA/NHNT separator retained its original shape even at 180 °C, thereby demonstrating excellent thermal stability. Furthermore, the separators were integrated into a LiFePO_4_|Li full battery to investigate electrochemical performance (Figure 10c). The OPVA separator exhibited lower capacity retention and coulombic efficiency than those of the Celgard separator, presumably owing to the presence of numerous hydroxyl groups that limited the transport of the electrolyte and Li ions. The coulombic efficiency of the battery with the OPVA/HNT separator decreased continuously and then gradually increased to a stable value of 65%. In contrast, the OPVA/NHNT separator exhibited a capacity of ≈145 mAh g^−1^, capacity retention of ≈100%, and coulombic efficiency of 100% after 100 cycles. This performance enhancement was due to the fact that the NHNTs had an enlarged pore space in addition to having a composition, chemical properties, and physical features similar to those of the HNTs. Furthermore, this structural advantage helps address safety concerns by suppressing dendritic Li growth (Figure 10d). The low stiffness of Celgard and OPVA separators cannot effectively block dendritic Li, which may penetrate the polymer matrix and cause short circuits. Their poor ionic conductivity also leads to uneven ion distribution, promoting lithium whisker formation. In contrast, the OPVA/NHNT separator's higher stiffness and ionic conductivity ensure uniform ion distribution and effectively suppress whiskers.

Similarly, Kim et al. fabricated an HNT‐coated PE separator for LMBs by soaking a PE separator in a solution containing HNTs and polydopamine (PDA) and then agitating the mixture at 200 rpm in an orbital shaker.^[^ ^55^ ^]^ PDA imparted hydrophilicity to the entire backbone of PE to improve electrolyte affinity. The electrolyte uptake of the PDA/HNT/PE separator (207.9%) was approximately three times that of the PE separator. Upon exposure to 130 °C for 30 min, the PDA/HNT/PE separator retained its initial dimensions and original structure, whereas the PE specimen only retained 42% of its initial dimensions. Additionally, the PDA/HNT/PE separator achieved a high Li^+^ transition value of 0.603, suggesting the inhibited formation of Li dendrites. To evaluate the electrochemical performance, the PDA/HNT/PE separator was assembled into a Li/LiFePO_4_ full cell. The Li/PE/LiFePO_4_ cell showed a capacity decrease after the 200th cycle, whereas the cell with the PDA/HNT/PE separator maintained a constant capacity even after the 500th cycle.

The substrates for inorganic coatings are not limited to conventional melt‐processed polymer membranes. For example, inspired by the excellent thermal stability and electrolyte wettability of cellulose, Huang et al. prepared composite nanofiber membranes for LIBs using bacterial cellulose (BC) and HNTs via vacuum filtration.^[^ ^53^ ^]^ BC, a renewable nanofiber material biosynthesized by *Gluconacetobacter xylinus*, exhibits high purity, crystallinity, porosity, and water absorbency. The composite separator with a BC/HNT ratio of 150:1 (BC/HNTs‐150) exhibited an excellent tensile strength of 84.4 MPa. This was attributed to the effective transfer of the external loads applied onto the BC nanofibers to the HNTs owing to the strong hydrogen bonding between the HNTs and BC nanofibers. Moreover, the BC‐HNTs‐150 separator exhibited a two‐times‐higher porosity than that of the PP‐PE‐PP separator (83.0% and 41.9%, respectively) and an almost three‐times‐higher electrolyte uptake than that of the PP‐PE‐PP separator (369% and 113%, respectively). Additionally, its ionic conductivity was notably higher than that of the BC and PP‐PE‐PP separators (5.13, 2.88, and 2.05 mS cm^−1^, respectively). Both the BC and BC/HNT membranes maintained their original dimensions even after heating at 180 °C for 30 min, unlike the commercial PP‐PE‐PP trilayer membrane, which dramatically shrank under the same conditions, indicating the excellent heat resistance of the composite membranes. When applied to a Li/LiFePO_4_ battery, the system with the BC/HNT separator demonstrated improved cycling properties and C‐rate capability compared with those of the cell with the BC separator.

Another intriguing cellulosic substrate tested for HNT coatings is commercial paper, as reported by Huang et al., who fabricated separators for LIBs by coating HNTs onto both sides of A4 paper and waste newspapers.^[^ ^52^ ^]^ This approach was evidently devised to produce highly stable separators using inexpensive, environmentally friendly materials and a practical coating process. Pure A4 paper, which primarily comprises cellulose and some CaCO_3_ particles, proved to be impractical as a separator because the specific capacity decreased by 100% after only six cycles, making HNT coating essential. Waste newspaper, a typical low‐cost cellulosic material, offers significant value for resource recycling and can be used directly as a substrate without any treatment. The hollow nanotube structure and abundant hydroxyl groups of the HNTs efficiently increased the wettability and electrolyte uptake, consequently improving the electrochemical performance of the composite separators. Moreover, owing to the high thermal stability and flame retardancy of the HNTs, the composite separators maintained their normal structure even after exposure to temperatures up to 180 °C; in contrast, the PP separator started to shrink at 120 °C and melted at 180 °C, completely losing its functionality. Remarkably, the HNTs@A4 paper and HNTs@newspaper specimens hardly changed even at temperatures above 200 °C. In Li/LiFePO_4_ cells, systems with the composite separators showed better cycling and rate performances than those of the cell assembled with a PP separator. Prior to cycling, the HNTs@A4 paper sample exhibited a higher ohmic resistance and charge‐transfer resistance (*R*_ct_) as it was thicker than the PP specimen. However, the *R*_ct_ value of the PP separator increased significantly after 100 cycles owing to the reaction of the HNTs with the electrolyte to form LiPO_2_F_2_—a Li salt that aids in reducing *R*_ct_. Additional experiments on the thermal stability of LIBs revealed that the HNT‐coated separators could prevent the explosion of LIBs under extreme loading conditions. This was confirmed from experiments in which graphite|HNTs@A4 paper|LiFePO_4_ pouch cells were fully charged to 3.8 V and then impaled with a 3‐mm‐diameter steel nail for 15 min, following which the voltage and surface temperature were measured. This impaling test is particularly significant as it simulates real‐world conditions where a battery might be pierced, potentially leading to a dangerous short circuit. Although the voltage decreased abruptly from 3.3 to 0 V, the temperature remained stable between 15 and 20 °C. Furthermore, the surface was clean without any signs of incineration, thereby providing an effective solution to prevent the thermal runaway of LIBs.

HNT coatings have also been employed in battery systems other than LIBs, such as Li–S batteries, whose commercialization has been hampered owing to the low electrical conductivity of sulfur, polysulfide shuttling between electrodes, and low capacity retention. Essentially, HNTs with Si–O tetrahedral sheets have abundant Lewis acid sites (Si–OH) and can thus capture polysulfides migrating through the separators during Li–S battery cycling, thereby preventing the direct reaction between polysulfides and the Li metal anode. Leveraging this concept, Kwon et al. coated one side of a commercial PP separator (Celgard 3501) with HNTs using a spin coater.^[^ ^57^ ^]^ The electrolyte contact angle of the HNT‐modified (H‐M) separators was considerably lower than that of the PP separators, resulting in greater hydrophilicity and wetting stability. To verify the ability of the HNTs to inhibit Li polysulfide shuttling, a standard CR2032 coin cell was fabricated using sulfur‐multi‐walled carbon nanotubes (S‐MWCNTs) as the active cathode material and metallic Li as the anode. The cell with the H‐M separator retained its capacity for up to 100 cycles because the migration of Li polysulfides was terminated during the discharge process. When the H‐M separator was discharged again at 100 mA h^−1^, a reversible capacity of 838 mAh g^−1^ was achieved, demonstrating good stability over a wide range of current densities.

Li organic batteries (LOBs) suffer from low coulombic efficiency and rapid capacity degradation owing to the high solubility of organic active materials. To address these issues, Gong et al. electrospun a PVA/HNT suspension (PVA/HNTs = 1:10 w/w) onto Al foil and crosslinked it with a dilute glutaraldehyde dispersion to prepare a crosslinked film (denoted EPH‐10).^[^ ^56^ ^]^ Glutaraldehyde functioned as a crosslinking agent that reacted with PVA to reduce the degree of swelling of the electrolyte and improve the mechanical strength of the PVA/HNT system. The EPH‐10 separator exhibited excellent electrolyte uptake and superporosity owing to its high volume‐to‐surface‐area ratio and 3D pore connectivity. Additionally, EPH‐10 presented various features that augmented the separator performance. For instance, no obvious dimensional changes were observed after 1 h of treatment at 200 °C. Additionally, the EPH‐10 membrane hindered the movement of organic redox intermediates owing to its interlaced network structure and strong negative charge repulsion effect. The electrochemical performance of EPH‐10 was evaluated using a battery (Li||AQ) with Li metal as the anode and anthraquinone (AQ) as the cathode. The results showed that the Li||AQ battery assembled with EPH‐10 exhibited a higher rate performance than that of the cells with Celgard or EP separators in the current rate range of 0.1C–0.5C. Furthermore, the capacity after 300 cycles (203.8 mAh g^−1^) was higher than that of other separators, and the battery showed good capacity retention. Additionally, after 100 cycles at 80 °C, a high initial discharge capacity was achieved along with a high capacity retention rate of 80.4%. This study demonstrated the potential of EPH‐10 as a composite separator for high‐temperature LOB applications.

As another prospective large‐scale energy system, the vanadium flow battery (VFB) offers advantages such as fast response times and long lifespans. Perfluorosulfonic membranes such as Nafion are typically used as the ion exchange membrane (IEM) in VFBs. However, these membranes are expensive and suffer from rapid capacity decay owing to the presence of ion clusters. To address these issues, Yu et al. fabricated an HNT‐based organic–inorganic composite membrane for VFBs using dopamine‐modified HNTs (DHNTs) and sulfonated poly(ether ether ketone) (SPEEK) via solution casting.^[^ ^43^ ^]^ To prevent corrosion in a strongly acidic environment, the surface of the HNTs was modified by CuSO_4_/H_2_O_2_‐induced rapid deposition. Owing to the coexistence of catechol and amine functional groups, the polydopamine layer was strongly adsorbed onto the surface of the HNTs. The polydopamine layers formed additional proton‐transport channels owing to the acid–base interactions between the –NH_2_ and –SO_3_H functional groups. Notably, a small amount of filler could not form orderly connected ion transport channels, whereas excess filler likely caused severe phase separation, leading to poor ion selectivity; consequently, the amount of HNTs used in the preparation of the SPEEK/HNT (S/HNT) composite membrane was carefully controlled. Specifically, a series of S/HNT composite membranes with different HNT loadings (1, 3, and 5 wt%) was fabricated (denoted S/HNTs 1%, S/HNTs 3%, and S/HNTs 5%, respectively). An analysis of the degree of sulfonation and water uptake of the fabricated membranes showed that the 3% S/HNT membrane was the most suitable for VFBs. Specifically, the SPEEK membrane with a high degree of sulfonation (0.79) exhibited higher water uptake and swelling ratio than those of a commercial Nafion 212 (N212) membrane. However, these properties tended to diminish with the introduction of the HNTs into the SPEEK matrix because the HNTs acted as physically crosslinked points that limited the movement of the polymer segments. The composite membranes exhibited the following trend in terms of the elongation percentage and breaking strength based on the presence/absence of a robust crosslinking network: S/DHNTs 3% > S/HNTs 3% > SPEEK. Moreover, the S/DHNTs 3% membrane exhibited the lowest VO^2+^ permeability, and its selectivity (3.1 × 10^5^ S min cm^−1^) was approximately three times and 16 times that of the pristine SPEEK membrane (1.0 × 10^5^ S min cm^−1^) and the N212 membrane (0.2 × 10^5^ S min cm^−1^), respectively. When the membranes were incorporated into a VFB single cell, the coulombic efficiency decreased in the order of S/DHNTs 3% > S/HNTs 3% > SPEEK >> N212 for all current densities. Furthermore, the S/DHNTs 3% membrane exhibited the highest energy efficiency among the specimens. Notably, the VFB assembled with the S/DHNTs 3% membrane achieved a capacity (≈15.5 Ah L^−1^) that was close to the theoretical capacity (20.1 Ah L^−1^) at an extremely high current density of 200 mA cm^−2^. The results of long‐term cycling tests conducted at 160 mA cm^−2^ over 500 charge–discharge cycles revealed an extremely stable coulombic efficiency (≈99%) and energy efficiency (≈78%), with an average capacity decay rate of 0.099% per cycle. Furthermore, compared to the N212 membrane, the S/DHNTs 3% membrane exhibited superior temperature tolerance over a wide temperature range (−20 to 60 °C).

#### 2.1.5 1D Mineral Summary

**Figure 11:**
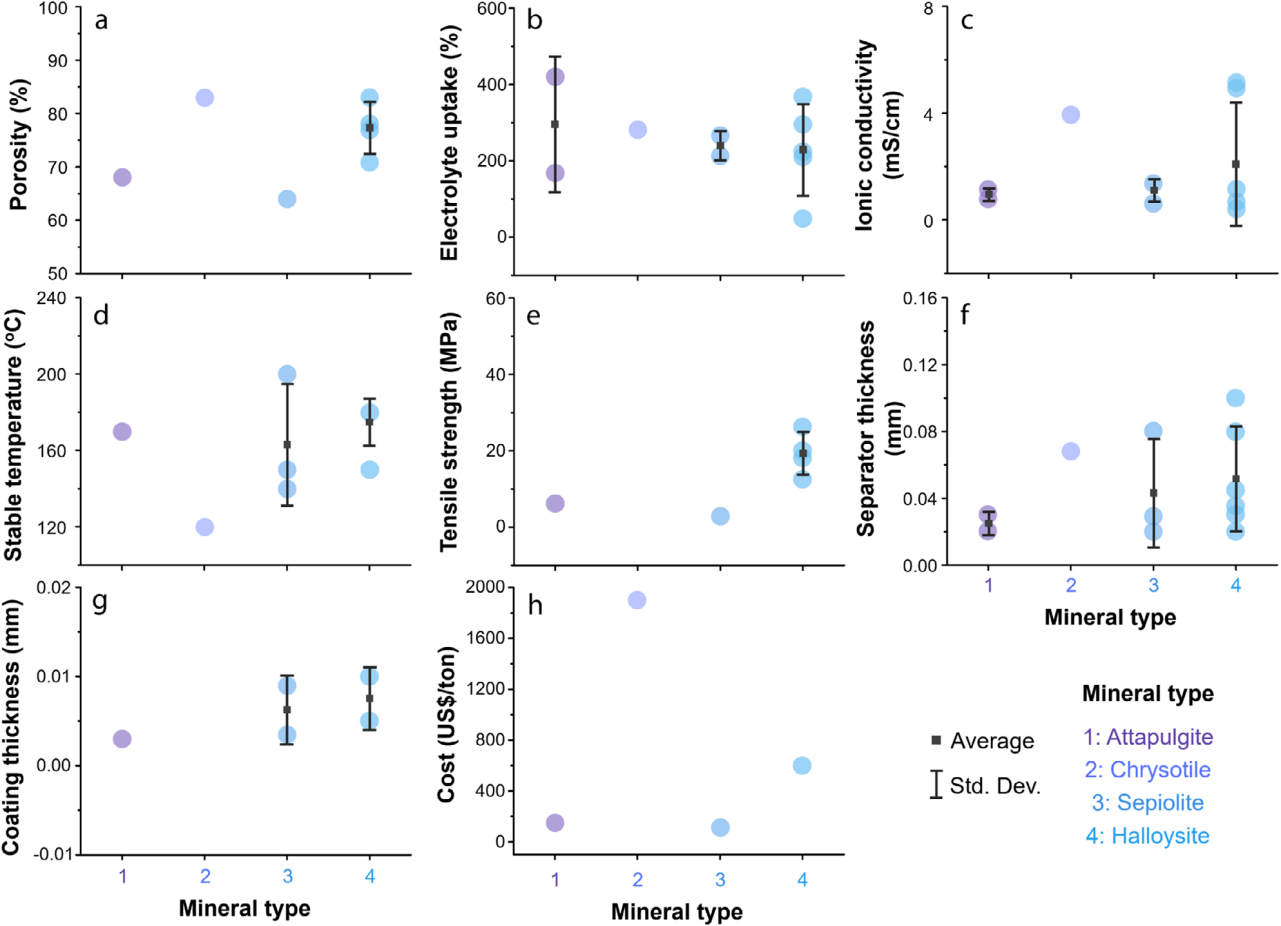
Dependence of separator parameters—a) porosity, b) electrolyte uptake, c) ionic conductivity, d) stable operating temperature, e) tensile strength, f) separator thickness, g) coating thickness, and h) cost —on the type of 1D mineral; Average and standard deviation of the data are indicated as black squares and vertical lines, respectively.

**Figure 12:**
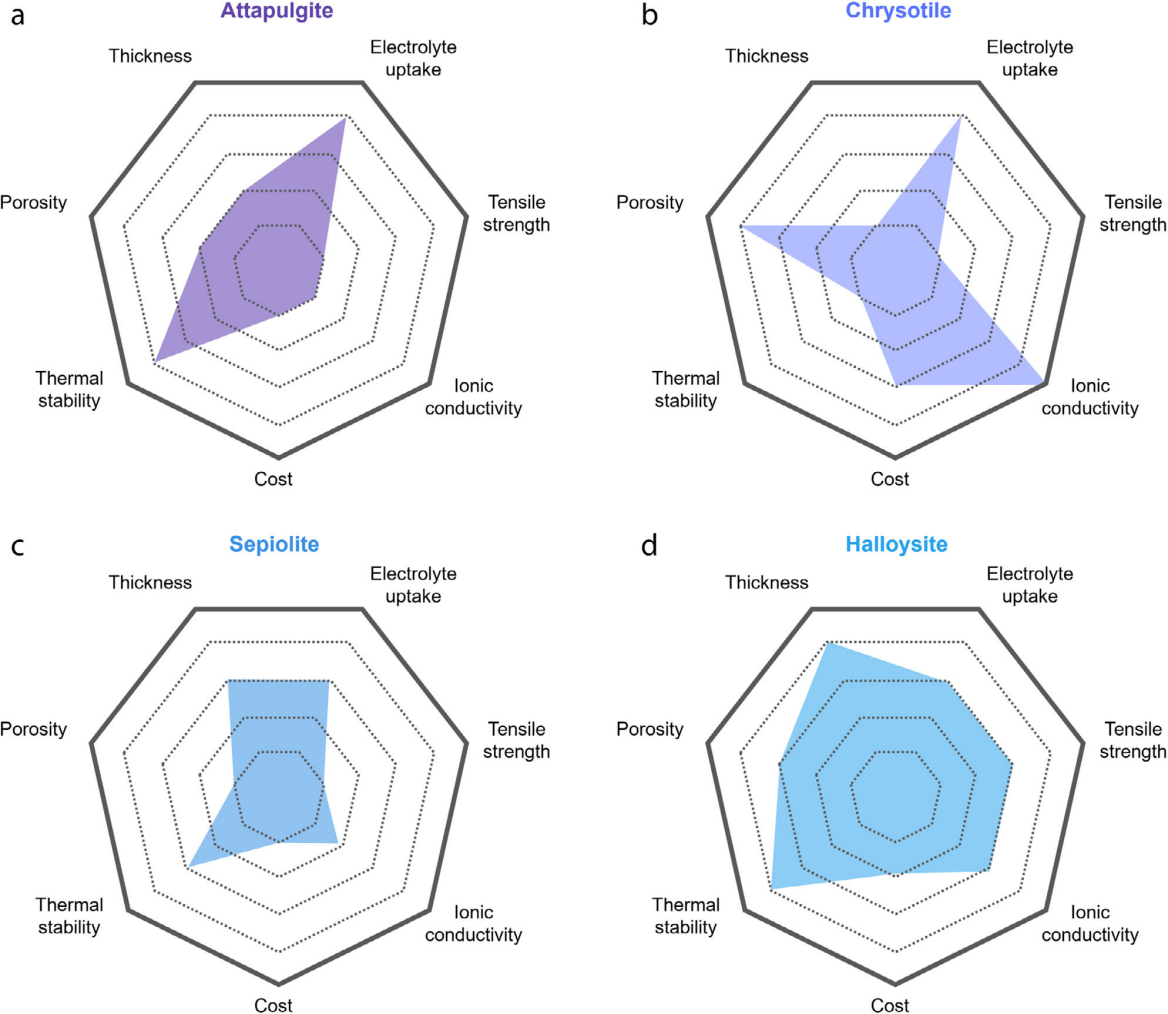
Radar plots for parameters of separators fabricated using different types of 1D minerals.

To understand how the type of 1D mineral affects membrane performance, we compared the reported performance indicators for each mineral (**Figures** **11** and **12**). First, in terms of electrolyte uptake, all 1D minerals generally exhibit high values, which is attributed to their fibrous structures that can absorb electrolyte molecules. Among them, halloysite and chrysotile show relatively high porosity and high ionic conductivity. Notably, halloysite features a multi‐layered rolled structure, and this robust morphology contributes to its high thermal stability. In terms of coating thickness, 1D mineral showed the following order: Halloysite > Sepiolite > ATP > Chrysotile. In terms of cost, chrysotile is significantly more expensive with ≈1900 US$ ton^−1^, compared to the other 1D minerals with their costs ranging for 100–600 US$ ton^−1^. Overall, while chrysotile may be preferred if only electrolyte uptake and ionic conductivity are considered, halloysite is regarded as the most promising coating material among 1D minerals when cost and thermal stability are also taken into account.

### 2.2 Lamellar (2D) Minerals

**Figure 13:**
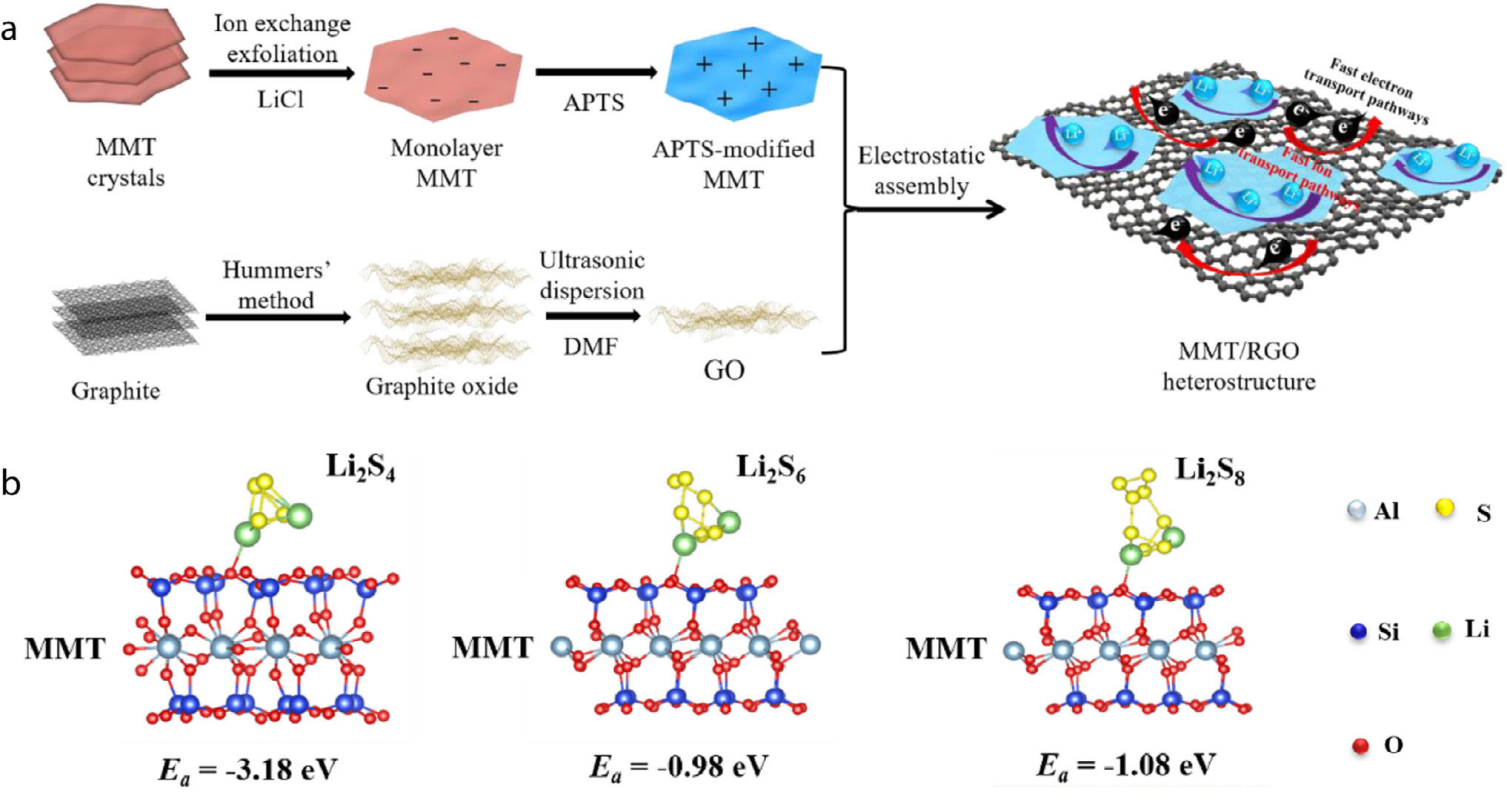
a) Exfoliated 2D montmorillonite sheets bearing exchanged Li⁺ cations form continuous fast‑ion channels between the layers, while the RGO network provides interconnected electron‑transport pathways. b) Density‐functional‐theory ‑optimized adsorption configurations and energies (E_a_) of Li_2_S_4_ (–3.18 eV), Li_2_S_6_ (–0.98 eV), and Li_2_S_8_ (–1.08 eV) on montmorillonite. The abundant Si–OH Lewis‑acid sites on the 2:1 montmorillonite surface selectively capture polysulfide species, suppressing shuttle effects in Li–S batteries. Reproduced with permission.^[^ ^100^ ^]^ Copyright 2022, Elsevier.

2D minerals contain platelet‐shaped particles at the microscale. This morphology is exhibited by crystal structures in which the interatomic bonds along a single direction are particularly weaker than those in other directions. For instance, albite is characterized by weak interatomic bonds along the {001} direction, which can be readily cleaved, leading to the formation of planar particles. Perfect bond cleavage also occurs in kaolin minerals, such as dickite and kaolinite, because the interlayer bonds between the 1:1 layered structures are comparatively weaker and can be readily ruptured. Bond breakage between layered structures is also commonly observed in smectite minerals. Specifically, the 2:1 layered structures of smectite minerals can easily split into platelet‐like particles because of the weak interatomic bonds between the (hydrated) alkali metal ions and the adjacent 2:1 layered structures. Representative examples of 2D smectite minerals include hectorite, illite, laponite, montmorillonite, and vermiculite. When 2D smectite minerals are used to fabricate separators for LIBs, their (hydrated) alkali metal ions can be readily replaced by Li^+^ ions and act as rapid Li diffusion channels, significantly enhancing the ionic conductivity of the separators (**Figure** **13**a). Additionally, 2D smectite minerals are particularly useful in Li–S batteries as they can effectively prevent polysulfide shuttling and the associated short circuits. Because of their 2:1 layered structure with two Si–O tetrahedral sheets, smectite minerals have abundant Si–OH groups, thereby providing numerous Lewis acid active sites that capture polysulfides migrating through the separators during Li–S battery cycling, thus preventing the direct reaction between polysulfides and Li metal anodes (Figure 13b).

**Figure 14:**
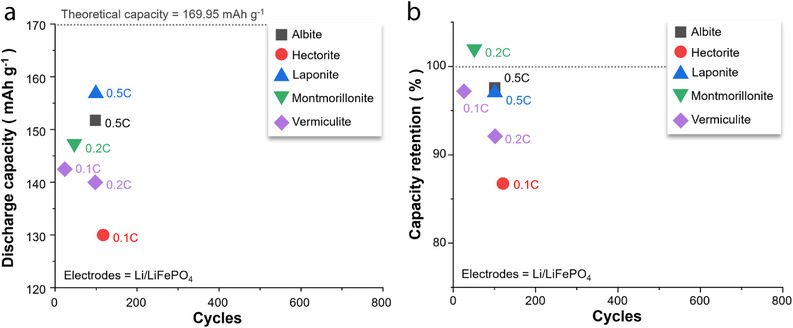
a) Discharge capacity–cycle number and b) capacity retention–cycle number plots for Li/LiFePO_4_ batteries with separators fabricated using lamellar minerals; The discharge capacity was estimated at the end of the cycling test, and the capacity retention was determined as the ratio of the first and last discharge capacities. The C‐rate used for each battery test is indicated next to the symbols.^[^ ^80^, ^82^, ^87^, ^96^, ^99^ ^]^

The aforementioned merits and the superior thermal and mechanical properties of 2D minerals have been leveraged to construct various battery separators (**Table** **2**). 2D minerals are particularly suitable for Li–S batteries, which require the prevention of the shuttling effect, and LIBs, which require separators with high ionic conductivity. In this review, the Li/LiFePO_4_ battery was selected as a representative system to analyze the performance of 2D‐mineral‐coated separators (**Figure** **14**). A comparison of five 2D‐mineral‐based LIBs shows that the system with laponite exhibits the highest discharge capacity of 156 mAh g^−1^ after 100 cycles, followed by albite and montmorillonite, which exhibit discharge capacities exceeding 145 mAh g^−1^ (Figure 14a). Although the discharge capacities of 2D‐mineral‐based separators (≈158 mAh g^−1^) are only slightly higher than those of 1D‐based systems (≈155 mAh g^−1^), it is important to note that the 2D materials achieved these values under higher C‐rate conditions, highlighting their superior rate capability. Moreover, a battery with a montmorillonite‐coated separator exhibits a capacity retention greater than 100%, suggesting that it could significantly improve the cyclability of LIBs (Figure 14b). Additionally, the discharge capacity of 2D‐mineral‐based batteries is generally higher than that of 1D‐mineral‐based batteries (Figure 2a), which may be partially attributed to the fast Li‐ion diffusion channels of 2D smectite minerals. Overall, 2D‐mineral‐coated separators exhibit superior performance owing to the combined effect of various factors such as the microstructure, type of host separator, and electrolyte, which are comprehensively summarized in the sections outlined below.

**Table 2:** Performance of composite separators based on lamellar minerals.

Mineral	Host separator	Method	Electrode materials	Liquid electrolyte	Capacity retention (number of cycles)	C‐rate	Reference
Albite	N/A	Solid‐phase sintering	Li, LiFePO_4_	LiPF_6_, 1:1 EC/DMC	99.74% (100)	0.5C	[80]
Dickite	ES	Coating	Li/Li	LiPF_6_, 1:1 EC/DEC	93.40% (200)	0.5C	[81]
Hectorite	PBI	Coating	Li, LiFePO_4_	LiPF_6_, 1:1 EC/DMC	97.42% (120)	0.1C	[82]
Illite	Celgard@2400	Coating	Li, CNT+S	LiTFSI (1 M) + LiNO_3_ (0.2 M), 1:1 DME/DOL	72.55% (500)	1C	[83]
Kaolinite	Glass fiber	Coating	Zn, MnO_2_	ZnSO_4_ (2 M) + MnSO (0.1 M)	69.14% (1000)	2C	[84]
Laponite	CB‐Celgard	Vacuum filtration	Li, S	LiTFSI (1 M) + LiNO_3_ (0.2 M), 1:1 DME/DOL	69.95% (500)	0.2C	[85]
	CNF	Sol–gel method	Li, NCM811	LiPF_6_, 1:1 EC/DMC	92.60% (100)	0.5C	[86]
	PVDF	Phase inversion	Li, LiFePO_4_	DMF	98.40% (100)	0.5C	[87]
Montmorillonite	PVDF	Electrospinning	Li, LiFePO_4_	LiPF_6_, 1:1:1 EC/EMC/DEC	96% (50)	0.2C	[88]
	PE	Coating	Li, S	LiTFSI (1 M), 1:1 DME/DOL	80% (450)	1C	[89]
	PES	Spray coating	Zn+Carbon felt, C	Zn(OH)_4_^2−^, Fe(CN)_6_^4−^	99.00% (200)	140 mA cm^−2^	[90]
	Celgard@2325	Coating	Graphite, LiNi_1.5_Mn_0.5_O_4_	LiPF_6_, 1:1 EC/DMC	63.64% (190)	5C	[91]
	PI	Solution blow spinning	Li, LiCoO_2_	LiPF_6_, 1:1:1 EC/EMC/DEC	86.60% (100)	0.5C	[92]
	PE	Coating	Li, LiNi_1/3_Co_1/3_Mn_1/3_O_2_	LiPF_6_, 1:1:1 EC/EMC/DEC	80% (100)	0.5C	[93]
	PP	Coating	Li, S	LiTFSI (1 M) + LiNO_3_ (0.2 M), 1:1 DOL/DME	93.40% (50)	0.06C	[94]
	PVDF	Phase inversion	Graphite, LiFePO_4_	LiPF_6_, 4:2:4 EC/DMC/DEC	98% (50)	0.2C	[95]
Vermiculite	Celgard@2500	Coating	Li, LiFePO_4_	LiPF_6_, 1:1 EC/DEC	98.67% (25)	0.1C	[96]
	Celgard@3401	Vacuum filtration	Li, S	LiTFSI, 1:1 DOL/DME	90.30% (50)	0.1C	[97]
	N/A	Electrospinning	Li, Li_4_Ti_5_O_12_	LiPF_6_, 1:1:1 EC/EMC/DEC	99.90% (200)	2C	[98]
	PVDF	Nonsolvent‐induced phase separation	Li, LiFePO_4_	LiPF_6_, 1:1:1 EC/DMC/EMC	95.70% (100)	0.2C	[99]

#### 2.2.1 Albite

**Figure 15:**
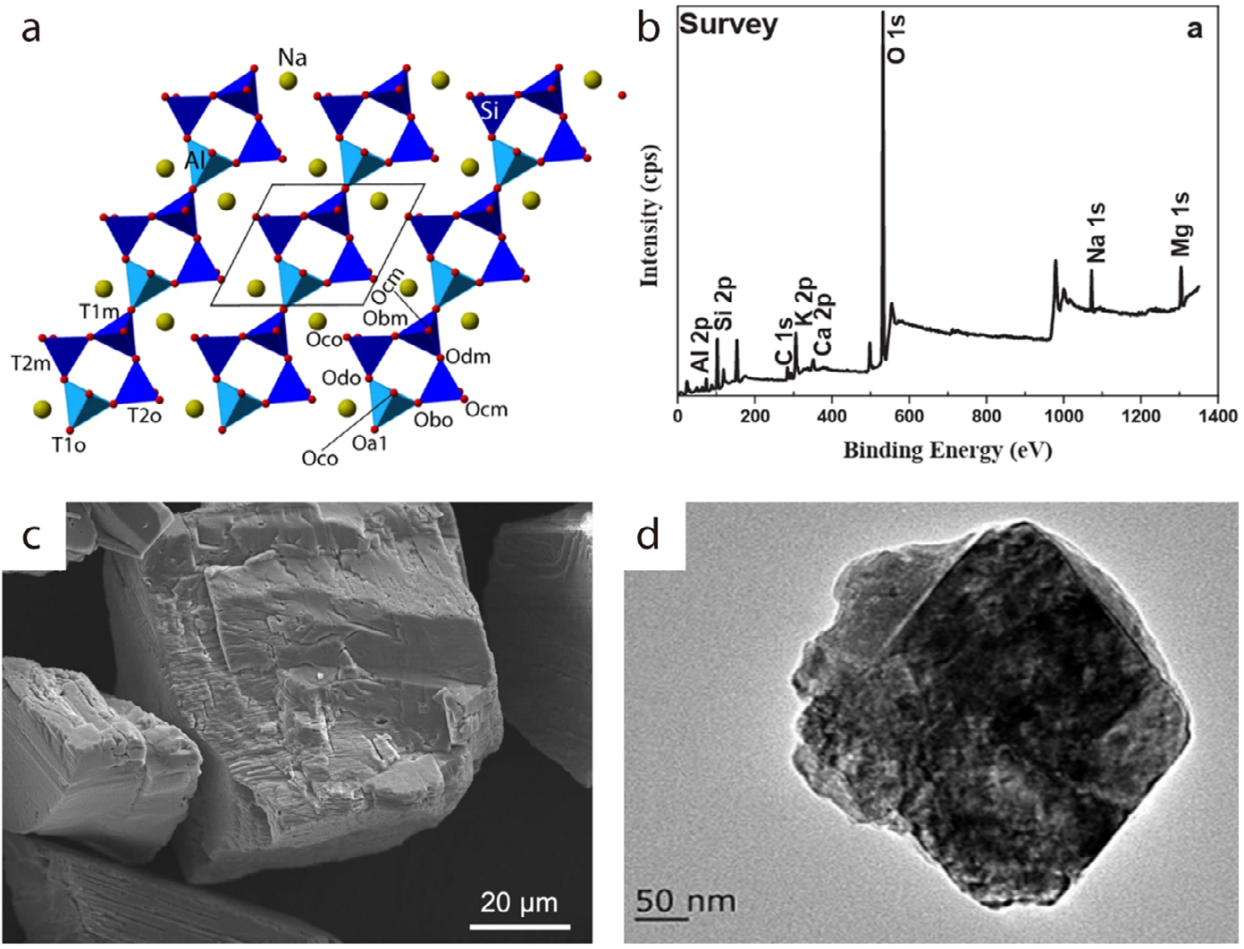
a) Slice of the albite structure (0.5 < y < 0.9), showing chains comprising four‐membered AlO_4_ and SiO_4_ tetrahedral units (light blue and dark blue, respectively) along the [001] direction, along with T–O–T linkages and Na atoms (golden spheres). Reproduced with permission.^[^ ^101^ ^]^ Copyright 2016, Nature. b) Full survey XPS scan of albite. Reproduced with permission.^[^ ^102^ ^]^ Copyright 2017, Elsevier. c) SEM image of single albite grains with a few fine particles at their surface. Reproduced with permission.^[^ ^103^ ^]^ Copyright 2024, Elsevier. d) TEM image of natural albite. Reproduced with permission.^[^ ^102^ ^]^ Copyright 2017, Elsevier.

Natural albite (NaAlSi_3_O_8_), which belongs to the plagioclase group of minerals and exhibits a sodium‐terminated composition, can also be categorized as a tectosilicate. It is abundantly found in pegmatitic masses and quartzite rocks such as granite. Additionally, it is distributed in the typical greenstone metamorphic layers in rocks, and also appears in certain hydrothermal vein deposits. The albite structure comprises SiO_4_ and aluminum–oxygen tetrahedra (AlO_4_), which are bonded together via oxygen atoms to form a 3D skeletal structure (**Figure** **15**a).^[^ ^101^ ^]^ Notably, Na^+^ ions can occupy the large interstitial sites within the skeletal structure of albite. Si and Al share the same positions, with the (Al and Si)/O atomic ratio being 1:2. X‐ray Photoelectron Spectroscopy (XPS) survey scans have confirmed the presence of Na, Al, Si, and O in albite (Figure 15b).^[^ ^102^ ^]^ Natural albite powder comprises large, regularly shaped particles, which contain a few fine particles on their surfaces (Figure 15c).^[^ ^103^ ^]^ It can also exist as planar‐shaped particles without micropores or mesopores, owing to the weak interatomic bonds along the {001} direction. Moreover, TEM imaging has confirmed that natural albite exhibits a small specific surface area (Figure 15d).^[^ ^102^ ^]^

**Figure 16:**
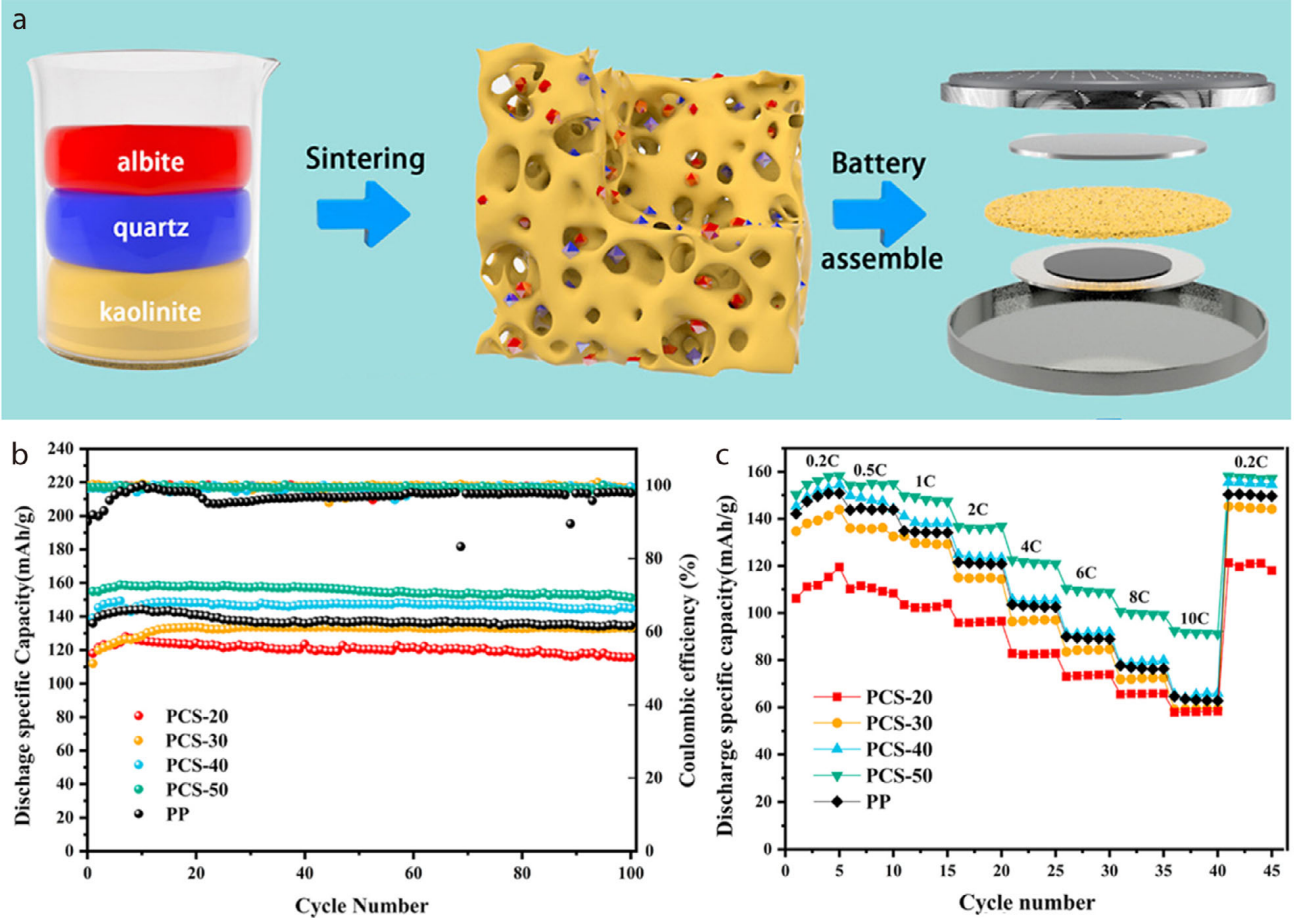
a) Schematic illustrating fabrication of porous ceramic separator and assembly of battery. b) Cycling performance and c) C‐rate performance of batteries assembled with different separators. Reproduced with permission.^[^ ^80^ ^]^ Copyright 2021, Elsevier.

Albite does not naturally contain pores; therefore, additional pore‐forming agents are required to fabricate albite‐based separators with porous structures. For instance, Wang et al. prepared porous ceramic separators (PCSs) for LIBs via solid‐phase sintering using ammonium bicarbonate (NH_4_HCO_3_) as the pore‐forming agent. This was achieved by mixing kaolinite, albite, and quartz in anhydrous ethanol at a mass ratio of 3:3:2 to form a slurry (**Figure** **16**a).^[^ ^80^ ^]^ After drying the clay mixture, different amounts of NH_4_HCO_3_ were added (20, 30, 40, or 50 wt%). The resulting mixture was pulverized, pressed into round pellets, and then sintered at 1100 °C to fabricate ceramic separators with varying amounts of NH_4_HCO_3_ (denoted PCS‐20, PCS‐30, PCS‐40, and PCS‐50). The contact angle of the PCSs was smaller than that of the PP separator, indicating good wettability for LiPF_6_, which reduced the Li^+^ migration resistance of the separator and enhanced the ionic conductivity, thereby improving the electrochemical performance of the battery. The cycling performance of cells with commercial PP separators or PCSs was analyzed at a current rate of 0.5C. The discharge capacity of the cells with PCS‐40 or PCS‐50 was higher than that of the cells with the PP separator (Figure 16b). Except for PCS‐20, all cells assembled with the PCSs exhibited a higher capacity retention rate and coulombic efficiency than those of the cell with the PP separator (93.22%) over 100 cycles. Furthermore, the cells with PCS‐40 or PCS‐50 showed a higher discharge capacity than that of the cell with the PP separator at current rates ranging from 0.2C to 10C (Figure 16c).

#### 2.2.2 Dickite

**Figure 17:**
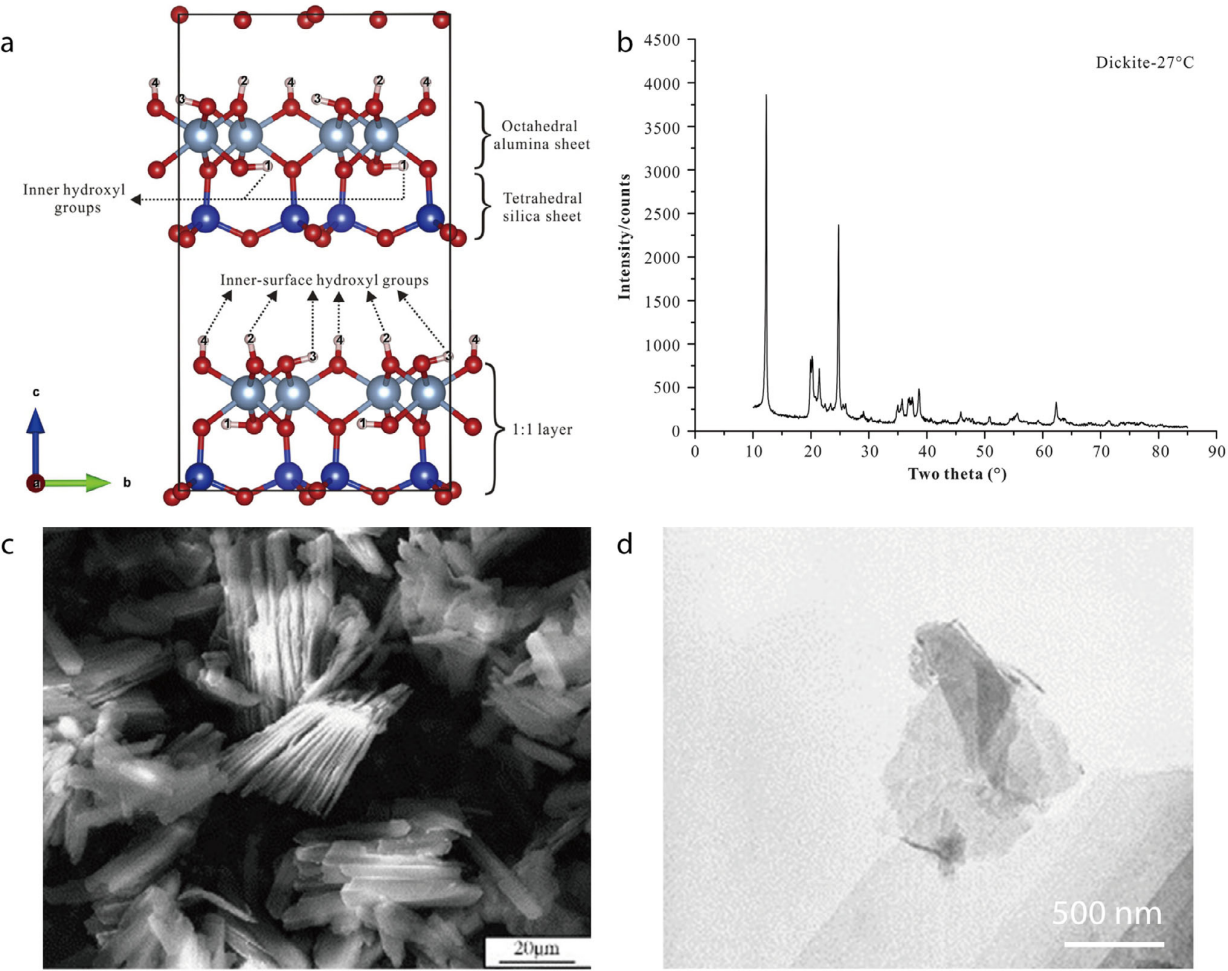
a) Structure of dickite. b) XRD pattern of dickite. Reproduced with permission.^[^ ^105^ ^]^ Copyright 2018, Elsevier. c) SEM image of untreated dickite. d) TEM image of untreated dickite. Reproduced with permission.^[^ ^106^ ^]^ Copyright 2007, Elsevier.

Dickite (Al_2_Si_2_O_5_(OH)_4_) is a 1:1 dioctahedrally structured clay mineral that belongs to the kaolinite group.^[^ ^81^, ^104^ ^]^ It has 2D interconnected channels with an interlayer spacing of 0.72 nm and can thus allow some Li ions to pass through (**Figure** **17**a).^[^ ^105^ ^]^ Additionally, the interactions between the Lewis acid sites of dickite and the anions of Li salts facilitate bond dissociation inside the Li salts, which releases more Li ions and enhances ionic conductivity. In its XRD pattern, dickite typically exhibits characteristic primary reflections at 7.21 and 3.59 Å (Figure 17b).^[^ ^105^ ^]^ SEM imaging has revealed that dickite crystals exhibit a highly uniform morphology with platelet‐like particles (Figure 17c).^[^ ^106^ ^]^ This morphology has been more clearly observed by TEM, and the size of dickite particles has been confirmed to be <1 µm (Figure 17d).^[^ ^106^ ^]^

**Figure 18:**
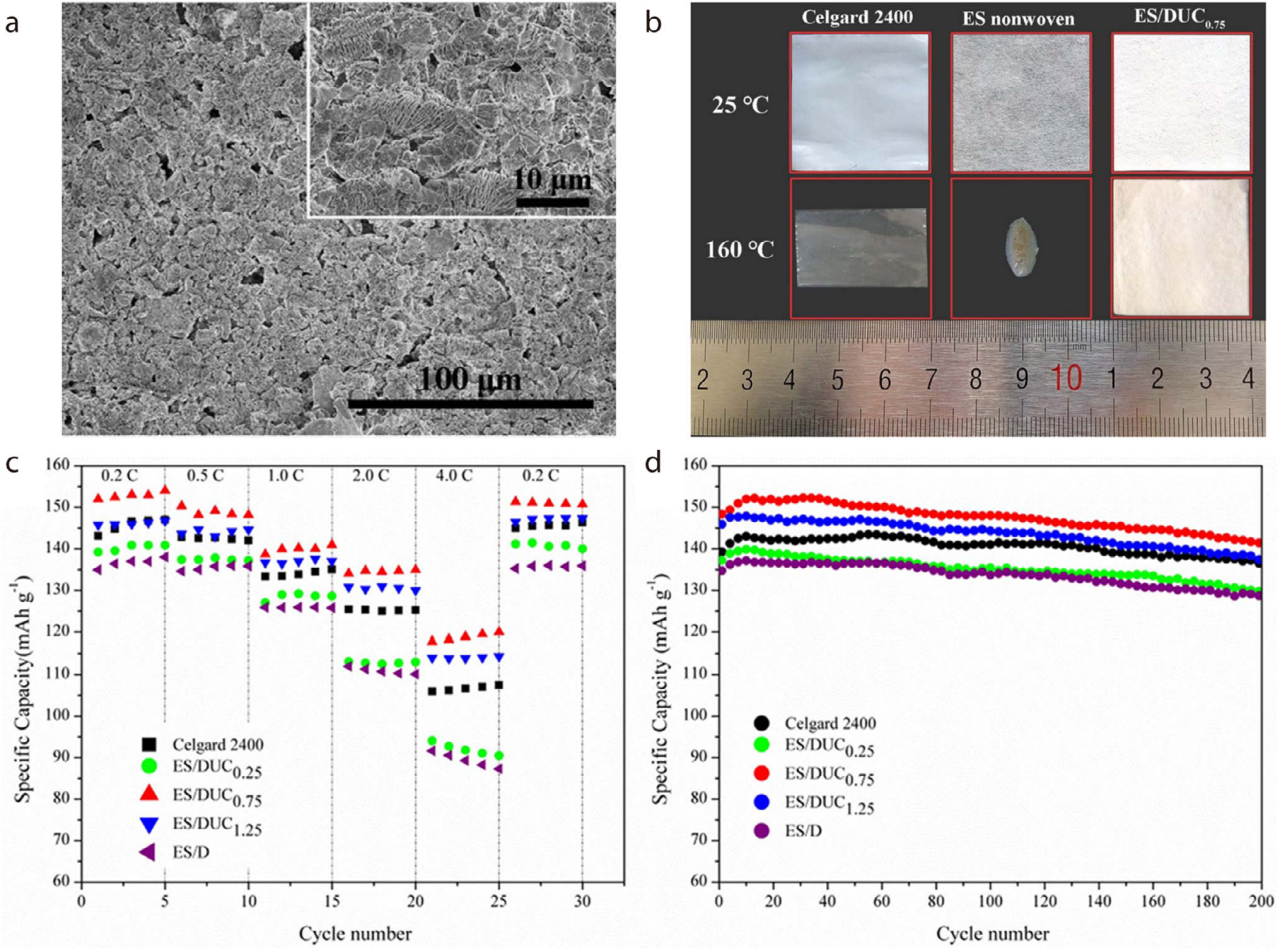
a) SEM image of ES nonwoven fabric coated with a dickite specimen expanded using urea (DUC_0.75_). b) Digital images of Celgard 2400, ES nonwoven fabric, and ES/DUC_0.75_ before (top line) and after (bottom line) heat treatment at 160 °C for 30 min. c) C‐rate capability and d) cycling performance of cells assembled with the Celgard2400 and composite separators. Reproduced with permission.^[^ ^81^ ^]^ Copyright 2019, Institute of Physics.

Notably, additional Li ions or electrolyte molecules can be stored between the layers in dickite. To maximize this characteristic, the interlayer spacing of dickite has been increased by adding large organic molecules between its layers. For instance, Liu et al. used urea to synthesize dickite with an enlarged interlayer spacing (samples denoted DU_δ_, with δ representing the urea/dickite ratio, that is, 0.25, 0.75, or 1.25; expanded dickite referred to as DUC_δ_). Subsequently, a composite separator was fabricated by dip‐coating a slurry of acrylonitrile multi‐copolymer binder (LA133) and DUC_δ_ in a mass ratio of 1:9 onto ethylene–propylene side‐by‐side (ES) nonwoven fabrics. When DUC_δ_ was used as a filler to coat the ES nonwoven fabric, the surface of the fabric was completely covered by DUC_δ_, which maintained its porous structure and unique morphology (**Figure** **18**a).^[^ ^81^ ^]^ Among the ES/DUC_δ_ separators, ES/DUC_0.75_ exhibited optimal electrolyte absorption owing to its high porosity. Moreover, ES/DUC_0.75_ showed enhanced thermal stability as it did not shrink at 160 °C (Figure 18b). The electrochemical performance of the composite separators was evaluated using a Li/separator/Li cell. When the rate performance of the cells was measured at different C‐rates, the discharge capacity of the cell assembled with ES/DUC_0.75_ was higher than that of the cells assembled with Celgard2400 or the other composite separators (Figure 18c). The cycling capacity was evaluated under constant charge–discharge conditions at 0.5C, and the results indicated that the capacity retention of the cell assembled with some of the DUC_δ_‐coated separators was somewhat lower than that of the cell with Celgard 2400 after 200 cycles (Figure 18d). This was due to the wide distribution of DUC_δ_ particles in the layer spacing, which resulted in an uneven current across the separator. However, the cell assembled with the ES/DUC_0.75_ separator showed the best cycling stability among the cells, including the one with commercial Celgard 2400.

#### 2.2.3 Hectorite

**Figure 19:**
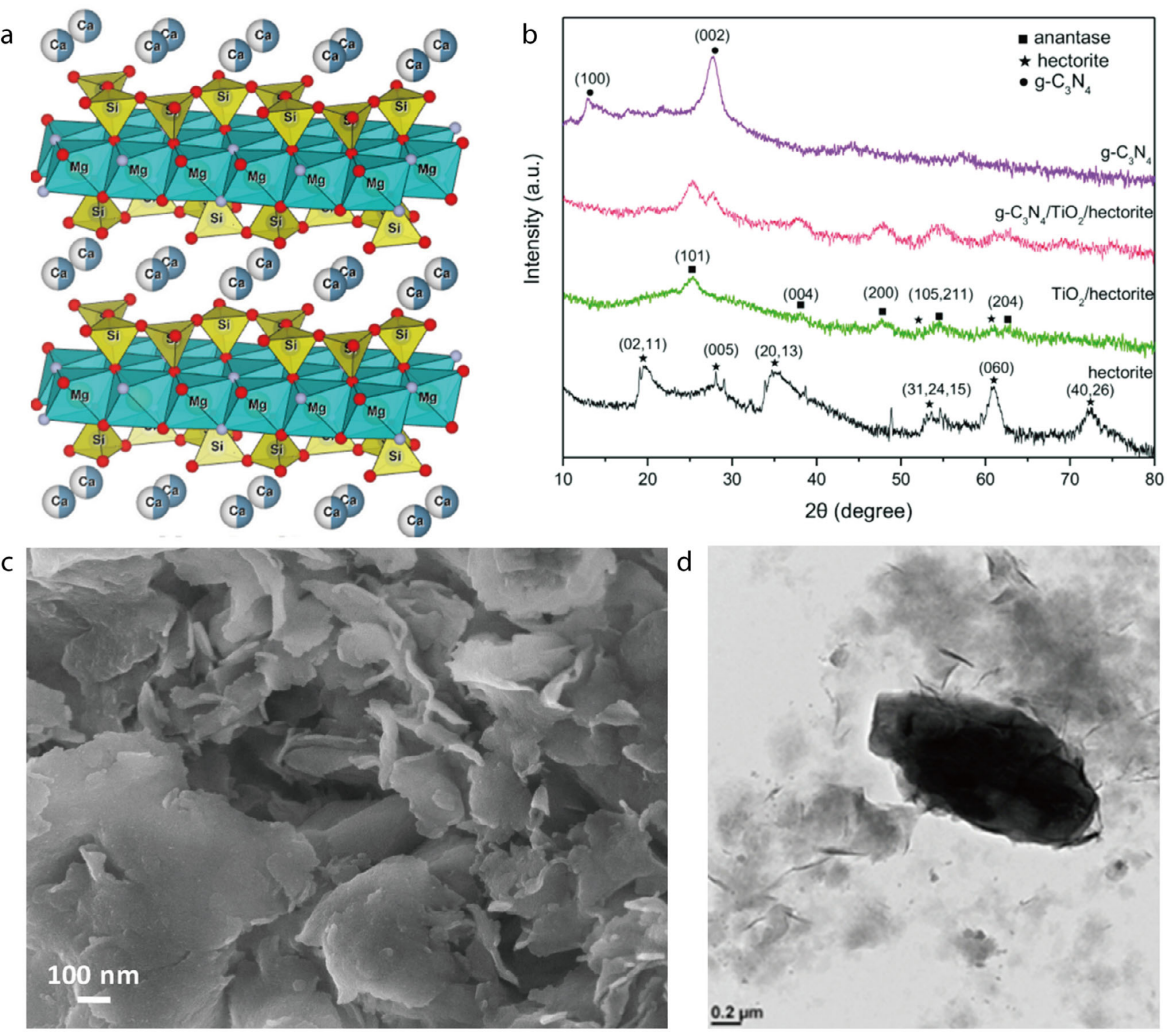
a) Molecular structure of HT. Reproduced with permission.^[^ ^107^ ^]^ Copyright 2023, Elsevier. b) XRD patterns of HT, TiO_2_/HT, g‐C_3_N_4_/TiO_2_/HT, and g‐C_3_N_4_. Reproduced with permission.^[^ ^108^ ^]^ Copyright 2020, Multidisciplinary Digital Publishing Institute. c) SEM image of HT. d) TEM image of pristine HT. Reproduced with permission.^[^ ^109^ ^]^ Copyright 2022, Elsevier.

Hectorite (HT) is a trigonal clay mineral with the formula Na_0.3_Mg_2.7_Li_0.3_Si_4_O_10_(OH)_2_. It is a natural layered Mg–Li silicate belonging to the smectite group and is characterized by a 2:1 layered structure featuring a single oxide octahedral sheet containing Mg or Li, bonded to two silicon oxide tetrahedral sheets (**Figure** **19**a).^[^ ^107^ ^]^ Hydrated cations with Li or Na ions can be inserted between the 2:1 layers, thus increasing the interlayer spacing. The edges and basal surfaces of the HT nanolayers are negatively charged because of the oxide‐type functional groups; nevertheless, this charge state can be reversed by submerging HT in liquid solutions under high‐pH conditions (>9.15). This charge reversal is particularly significant for separator applications, as it can modify the electrostatic interactions between HT and Li ions or polymer matrix components. By adjusting the surface charge, HT can facilitate more uniform Li‐ion migration, reduce ion aggregation, and improve compatibility with separators. HT exhibits a specific surface area of ≈350 m^2^ g^−1^ and a high cation‐exchange capacity (50–150 mmol 100 g^−1^) over a pH range of 6–13. In its XRD pattern, HT shows diffraction peaks at 2*θ* values of approximately 19.6°, 28.0°, 35.1°, 53.3°, 61.0°, and 72.3° (Figure 19b).^[^ ^108^ ^]^ Moreover, HT exhibits a petal‐stacked structure with curled edges at the microscale (Figure 19c) and a lamellar structure at the nanoscale (Figure 19d).^[^ ^109^ ^]^ HT has significant potential as a drug delivery vehicle for applications in biology, tissue engineering, and regenerative medicine.

**Figure 20:**
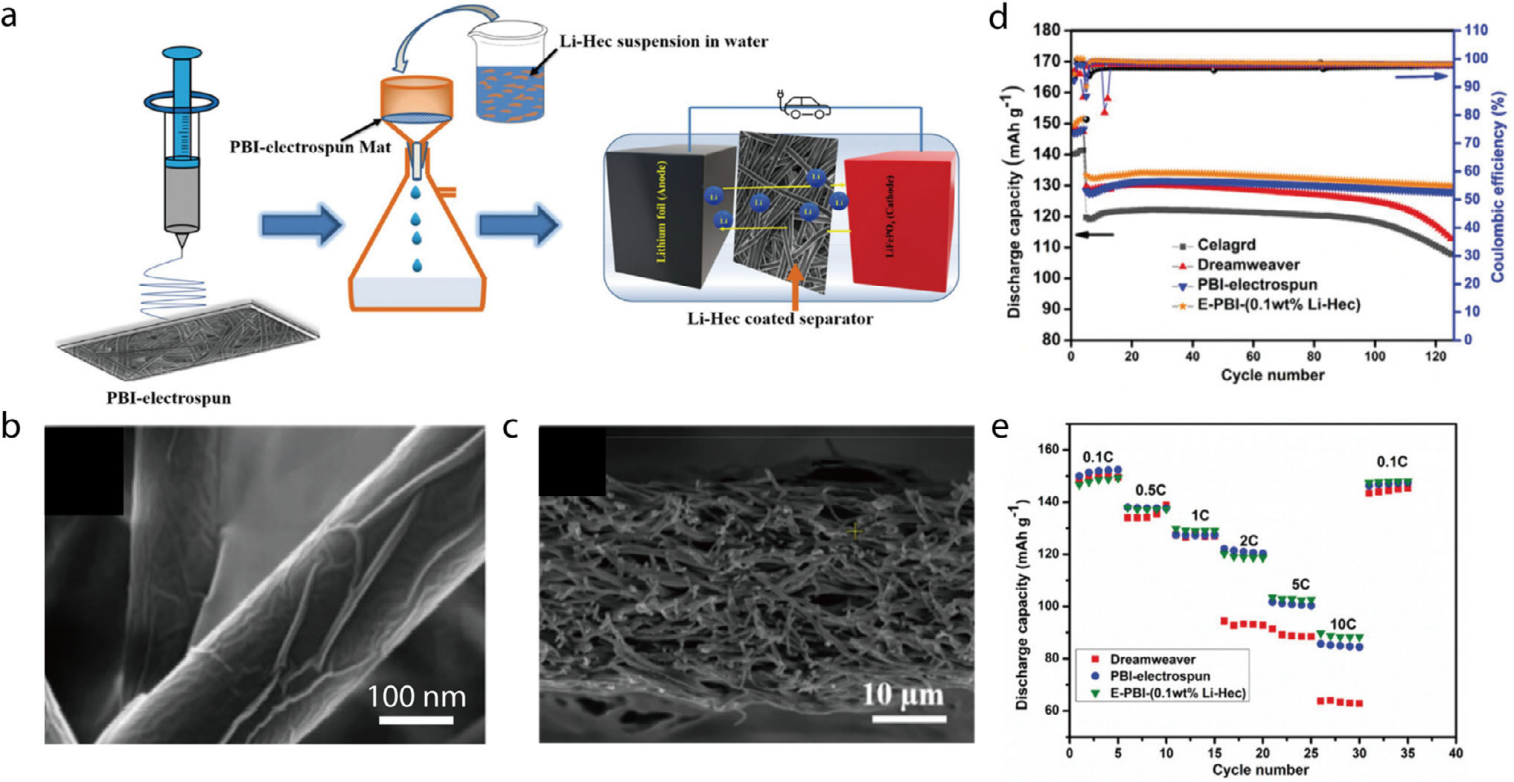
a) Schematic of preparation of Li‐HT coated electrospun PBI separator. b) SEM image of electrospun PBI membrane coated with Li‐HT (final sample denoted E‐PBI‐(0.1 wt% Li‐HT)). c) EDX‐based Si‐atom mapping of a cross‐section of E‐PBI‐(0.1 wt% Li‐HT). d) Cycling performance of coin cells assembled with Celgard, Dreamweaver, PBI‐electrospun, or E‐PBI‐(0.1 wt% Li‐HT) separators; Of the 125 cycles, the first four were formation cycles at 0.1C, whereas the subsequent 121 cycles were performed at 1C. e) Rate performance of Dreamweaver, PBI‐electrospun, and E‐PBI‐(0.1 wt% Li‐HT) separators integrated into coin cells at current rates of 0.1C, 0.5C, 1C, 2C, 5C, and 10C, and reverting to 0.1C. Reproduced with permission.^[^ ^82^ ^]^ Copyright 2023, Wiley‐VCH.

Among the various HT specimens, the variant with hydrated Li ions between the oxide layers (denoted Li HT (Li‐HT)) has inherent 2D diffusion slits offering high Li‐ion conductivity. To exploit this feature, Li‐HT has been coated onto separators to improve ionic conductivity while ensuring high mechanical stability. For instance, Joshi et al. prepared battery separators by coating Li‐HT onto thin, high‐temperature‐stable porous electrospun polybenzimidazole (PBI) membranes (**Figure** **20**a).^[^ ^82^ ^]^ The pristine and Li‐HT‐coated electrospun membranes (E‐PBI and E‐PBI‐(0.1wt% Li‐HT), respectively) exhibited considerably higher solvent uptake capacity (552% and 413%) than that of commercial separators such as Celgard and Dreamweaver. SEM imaging indicated that E‐PBI‐(0.1 wt% Li‐HT) exhibited a wrinkled surface as it was covered with Li‐HT nanosheets (Figure 20b). The Li‐HT coating on the PBI fibers was corroborated by Si‐atom mapping via energy dispersive X‐ray analysis (EDX) analysis of a cross‐sectional cut of the separator (Figure 20c). Moreover, the coated specimen showed excellent dimensional stability, in contrast to Celgard, which exhibited unidirectional shrinkage at 200 °C. The cell performance was tested by assembling a LiFePO_4_|separator|Li coin cell, and the results suggested that E‐PBI‐(0.1 wt% Li‐HT) showed higher capacity retention (97.42%) than that of the commercial separators (Figure 20d). Additionally, E‐PBI‐(0.1 wt% Li‐HT) exhibited higher rate performance than that of the commercial Dreamweaver separator. This was attributed to the high wettability, high porosity, high electrolyte uptake, low internal resistance, and high Li‐ion conductivity of Li‐HT (Figure 20e).

#### 2.2.4 Illite

**Figure 21:**
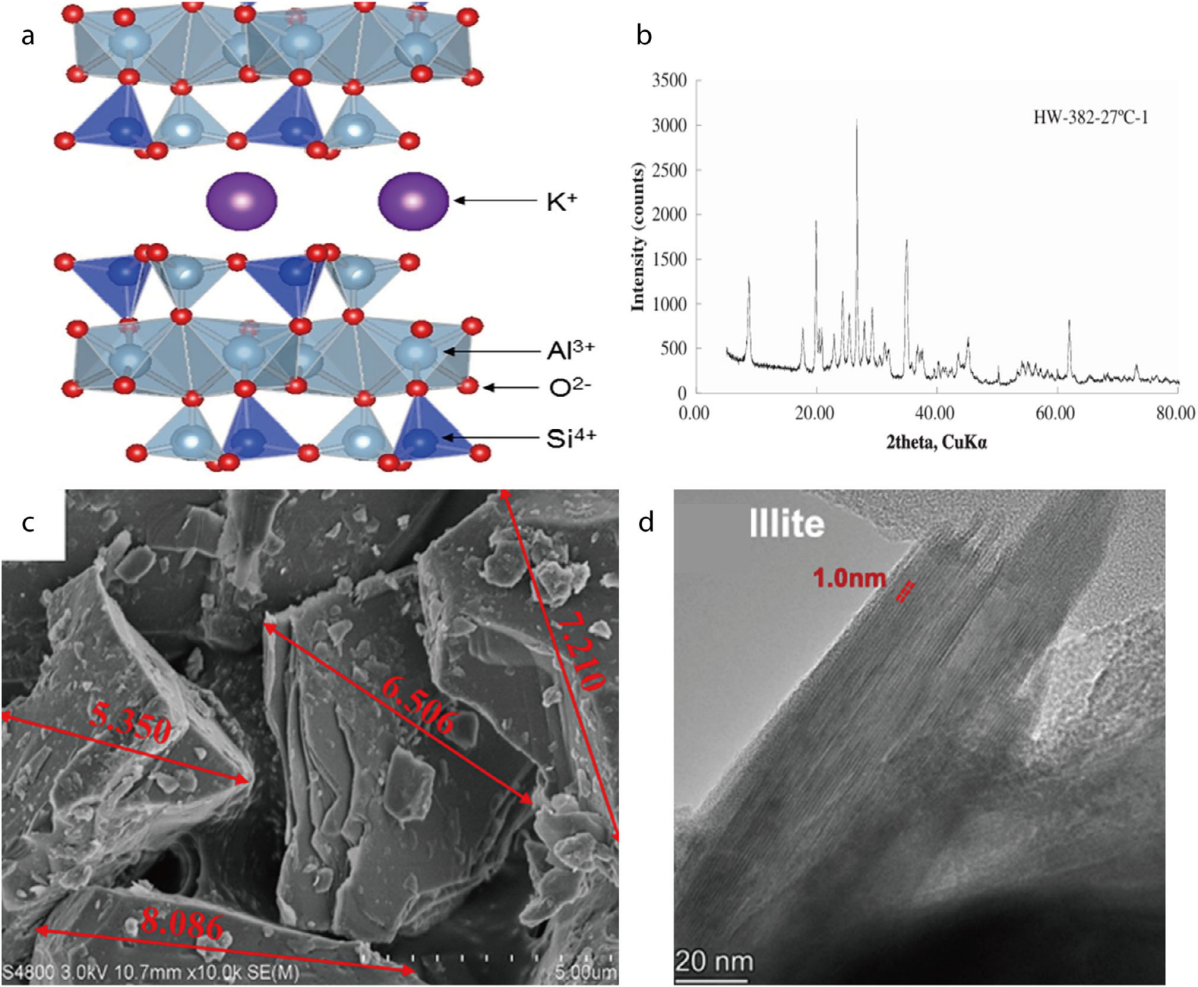
a) Structure of illite. Reproduced with permission.^[^ ^110^ ^]^ Copyright 2020, Elsevier. b) XRD pattern of illite at ambient temperature. Reproduced with permission.^[^ ^111^ ^]^ Copyright 2017, Elsevier. c) SEM image of pristine illite. Reproduced with permission.^[^ ^112^ ^]^ Copyright 2024, Elsevier. d) TEM image of illite. Reproduced with permission.^[^ ^113^ ^]^ Copyright 2023, Elsevier.

Illite ((K, H_3_O)Al_2_(Si_3_Al)O_10_(H_2_O, OH)_2_) is a natural mineral belonging to the mica group and an altered product of muscovite or potassium feldspar in weathering and hydrothermal environments. It has a 2:1 structure consisting of an octahedral sheet with Al as the central atom, surrounded by two silicon oxide tetrahedral sheets (**Figure** **21**a).^[^ ^110^ ^]^ Illite exhibits a cation‐exchange capacity of 10–40 meq 100 g^−1^ and a specific surface area of 50–100 m^2^ g^−1^. Illite is characterized as a mixed oxide based on its XRD pattern and exhibits a bulk chemical composition of 57.5% SiO_2_, 20.8% Al_2_O_3_, 6.9% FeO, and 7.4% K_2_O, with no pyrite and very little quartz (7%) (Figure 21b).^[^ ^111^ ^]^ Illite particles are 5.35–8.086 µm in size, indicating that the narrow passages between pores are too small for any material to move through (Figure 21c).^[^ ^112^ ^]^ At the nanoscale, illite has a linear layer structure and an interlayer distance of 1.0 nm in the (001) plane (Figure 21d).^[^ ^113^ ^]^

**Figure 22:**
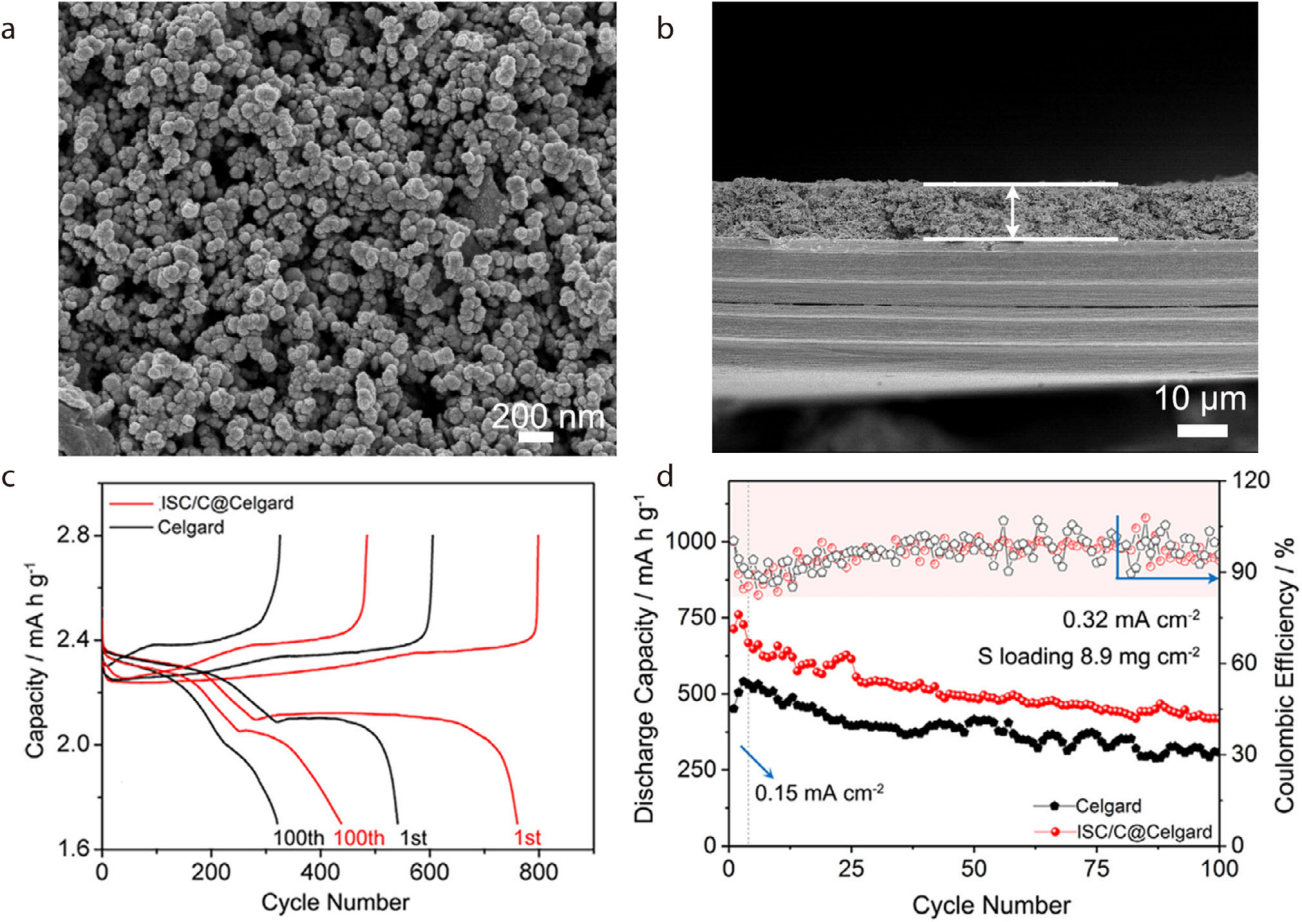
a) SEM image of ISC/C@Celgard separator. b) Cross‐sectional SEM image of ISC/C@Celgard separator. c) Charge–discharge profiles. d) Cycling stability, and coulombic efficiency of Li–S batteries fabricated using a self‐supporting CNT/S cathode and the different ISC‐based separators. Reproduced with permission.^[^ ^83^ ^]^ Copyright 2020, Elsevier.

A useful feature of illite is the abundant –OH groups on the surface of its 2:1 layered structure, which provide many active sites to trap polysulfides. This characteristic can be leveraged in Li–S batteries to prevent polysulfide shuttling and the associated severe safety issues. For instance, Wang et al. prepared illite/smectite (ISC)/C@Celgard separators for Li–S batteries by coating ISC and carbon black (CB) onto one side of a Celgard separator via simple blade coating. Because ISC increased the interfacial resistance owing to its electronically insulating nature, CB with high electrical conductivity was additionally coated. A Celgard separator, which was selected as the control specimen in the aforementioned study, exhibits a highly uneven pore size of several hundred nanometers, which allows polysulfides to pass freely and react with the Li anode, causing Li‐anode degradation and substantial loss of active sulfur. Nevertheless, the separator performance can be enhanced by adding carbonaceous materials, including CNTs and CB, because their high electrical conductivity can reduce the internal resistance. However, polysulfide shuttling is barely suppressed by carbonaceous materials as they weakly interact with polysulfides. In the aforementioned study, the ISC separator coated with CB (ISC/C) contained 30–100‐nm‐sized pores and could remove sulfides by physical shielding and strong chemical adsorption (**Figure** **22**a).^[^ ^83^ ^]^ The thickness of the ISC/C layer was 10 µm, according to a cross‐sectional SEM image (Figure 22b). Furthermore, when ISC/C was spread onto a Celgard separator, the resulting membrane (ISC/C@Celgard) exhibited good mechanical stability owing to the ISC–PVDF‐binder interactions. The ISC/C layer enhanced the electrolyte wettability of the Celgard separator owing to its uniform porous surface and the intrinsic electrolyte affinity of ISC. This promoted Li^+^ transport, which improved the rate performance and reduced the resistance of the Li–S battery. The electrochemical performance of the ISC/C@Celgard separator was evaluated by assembling a Li–S battery into a CR2032 coin cell in an argon atmosphere with a CNT/S cathode. The charge and discharge profiles of the batteries were acquired at a current rate of 0.1C, and the initial discharge capacity of the battery with the ISC/C@Celgard separator (1322 mAh g^−1^) was found to be considerably higher than that of the batteries with Celgard, C@Celgard, or CNT@Celgard separators. At a rate of 2.0C, the discharge capacity of the battery with the ISC/C@Celgard separator (701.7 mAh g^−1^) was higher than that of the batteries with the other separators. The discharge capacity remained high when the rate was reduced to 0.2C (870.1 mAh g^−1^), indicating a capacity retention of 91.8%. Additionally, the ISC/C@Celgard separator exhibited the slowest capacity decay at 1C, with a decay rate of 0.054% per cycle, which was significantly lower than that of the other separators. After pre‐activation at 0.15 mA cm^−2^ for three cycles, the battery with the ISC/C@Celgard separator and the self‐supporting CNT/S cathode achieved a high capacity of 667.3 mAh g^−1^ at 0.32 mA cm^−2^ (Figure 22c), which was considerably higher than that of the battery with the Celgard separator (532.1 mAh g^−1^). After 100 cycles at 0.32 mA cm^−2^, the ISC/C@Celgard separator retained a higher discharge capacity (418.6 mAh g^−1^) than that of the Celgard separator (308.1 mAh g^−1^) (Figure 22d). Moreover, the battery with the ISC/C@Celgard separator demonstrated a more stable coulombic efficiency than that of the cell with the Celgard separator.

#### 2.2.5 Kaolinite

**Figure 23:**
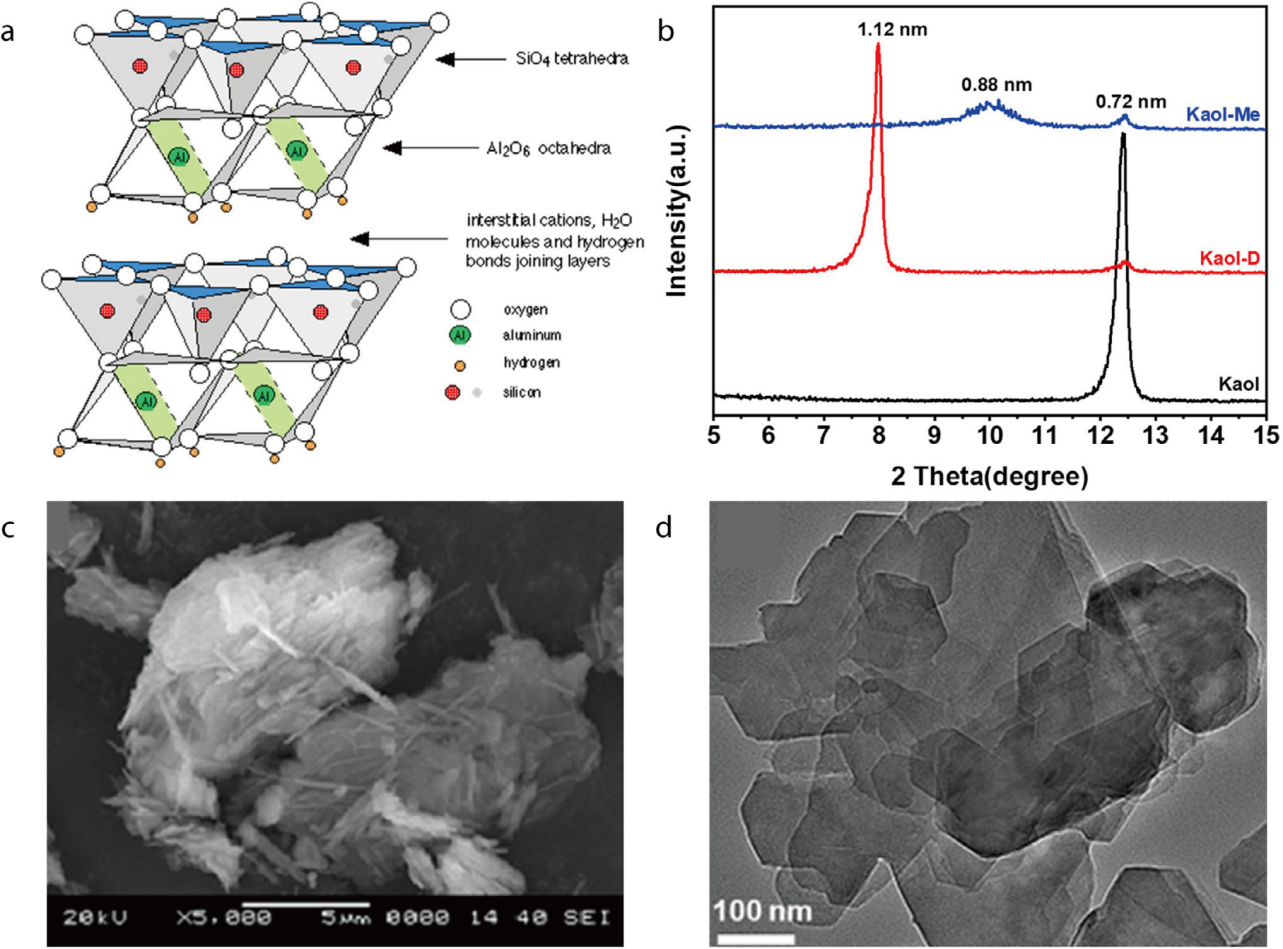
a) Structure of KL featuring Si, Al, and OH groups. Reproduced with permission.^[^ ^114^ ^]^ Copyright 2017, Taylor & Francis. b) XRD patterns of KL, KL/dimethyl sulfoxide intercalation composite (KL‐D), and KL/methanol intercalation composite (KL‐Me). Reproduced with permission.^[^ ^116^ ^]^ Copyright 2022, Elsevier. c) SEM image. Reproduced with permission.^[^ ^117^ ^]^ Copyright 2012, Elsevier. d) TEM image. Reproduced with permission.^[^ ^116^ ^]^ Copyright 2022, Elsevier.

Kaolinite (KL; Al_4_Si_4_O_10_(OH)_8_) is formed through the chemical weathering of Al‐rich silicates such as muscovite and feldspar. It has a 1:1 structure consisting of an octahedral sheet with central Al^3+^ cations and a tetrahedral sheet with central Si^4+^ cations (**Figure** **23**a).^[^ ^114^ ^]^ In KL, the 1:1 layers are connected to each other by hydroxyl groups on the octahedral Al sheets.^[^ ^115^ ^]^ KL is typically white; however, it can also appear green or yellow depending on the amount of iron hydroxide in it. It is refractory, soft, and SiO_2_‐rich. Moreover, it is a nonswelling clay mineral with no exchangeable cations in the interlayer space, which results in a low cation‐exchange capacity (1–15 meq 100 g^−1^) and low specific surface area (10–20 m^2^ g^−1^). In its XRD pattern, KL exhibits a characteristic peak at 2*θ* values of 12.36° (001), which can shift depending on the type of externally introduced intercalated molecules, even though it lacks naturally exchangeable cations in the interlayer space (Figure 23b).^[^ ^116^ ^]^ At the microscopic scale, KL evidently contains plate‐like or lamellar structures (Figure 23c).^[^ ^117^ ^]^ TEM imaging has indicated that kaolin particles are regular polygonal sheets stacked together (Figure 23d).^[^ ^116^ ^]^ KL has been applied to rubber fillings, paper coatings, cement, and ceramic materials.

**Figure 24:**
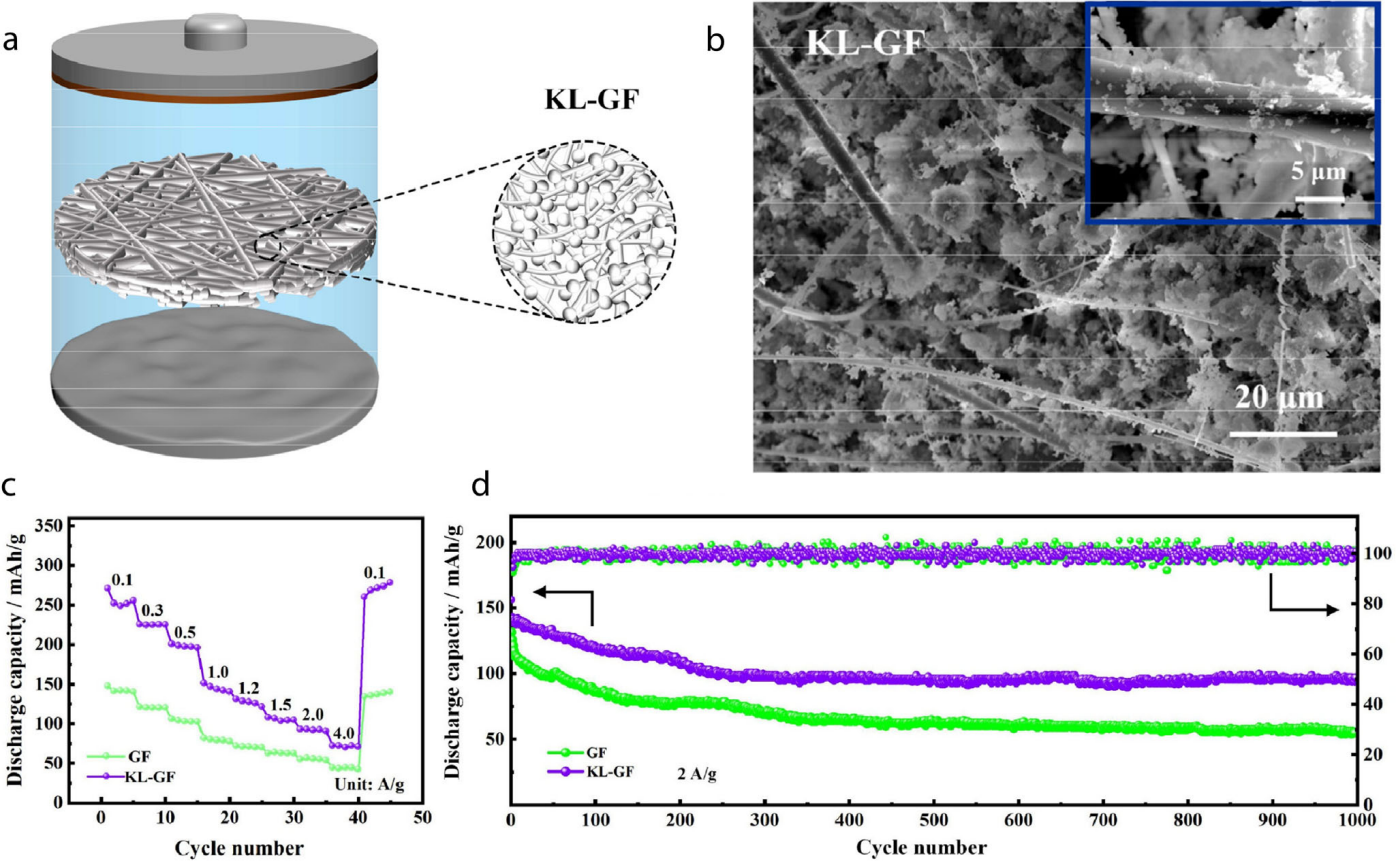
a) Model diagram of cell with pristine or KL‐modified glass fiber (GF or KL‐GF, respectively) systems, with the magnified image showing the structure of KL‐GF. b) SEM image of KL‐GF at different magnifications. c) Rate performance of Zn|GF|MnO_2_ and Zn|KL‐GF|MnO_2_ cells. d) Cycling performance and coulombic efficiency of both cells at 2 A g^−1^. Reproduced with permission.^[^ ^84^ ^]^ Copyright 2024, Elsevier.

Because of its unique layered structure, KL offers numerous channels for ion migration and exhibits impressive mechanical properties that can prevent dendrite growth on the electrodes. To exploit these merits, KL has been used as a filler material for battery separators to suppress the growth of anode dendrites while permitting ion transport. For example, Zhao et al. prepared a KL‐modified glass fiber (KL‐GF) separator to enhance the performance of aqueous Zn‐ion batteries (AZIBs) (**Figure** **24**a).^[^ ^84^ ^]^ The KL in the KL‐GF separator grew uniformly on the surface of the GFs (Figure 24b), thereby solving a common problem of GF separators that hinders uniform ion migration and promotes dendrite formation during electrode coating and stripping. GF separators hardly prevent dendrite growth on Zn anodes; however, KL has potential for suppressing dendrite growth in AZIBs as it is a functional material with good mechanical properties and abundant zincophilic sites. Additionally, KL exhibits excellent wettability, a large surface area, and high ionic conductivity, similar to GF. The performance of the fabricated separators was further evaluated by incorporating KL‐GF into a Zn//MnO_2_ cell. The capacity of the Zn|KL‐GF|MnO_2_ cell was always higher than that of the Zn|GF|MnO_2_ cell at different current densities (Figure 24c). The cell with KL‐GF demonstrated a high discharge capacity of 96.8 mAh g^−1^ after 1000 cycles at 2 A g^−1^, indicating good reversibility and stability (Figure 24d). Furthermore, after cycling and resting, the discharge capacity of the full cell with GF decreased significantly to 81.5 mAh g^−1^, whereas that of the full cell with KL‐GF was maintained at 161.6 mAh g^−1^. This indicated the favorable contributions of KL‐GF to the ability of the cell to recover after resting.

#### 2.2.6 Laponite

**Figure 25:**
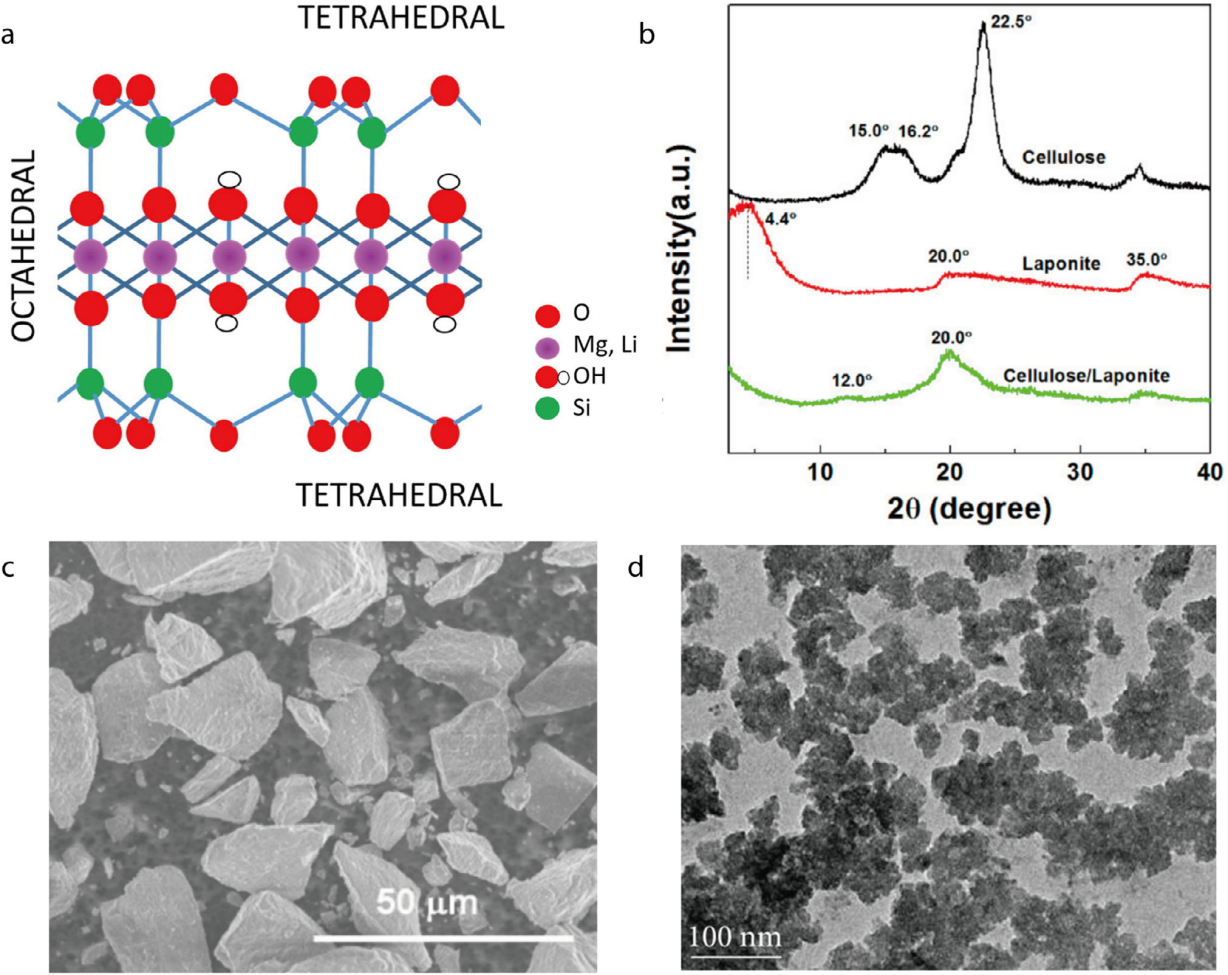
a) Atomic structure of laponite. Reproduced with permission.^[^ ^120^ ^]^ Copyright 2018, Elsevier. b) XRD pattern. c) SEM image of laponite. Reproduced with permission.^[^ ^121^ ^]^ Copyright 2021, Multidisciplinary Digital Publishing Institute. d) TEM image of nanosized laponite. Reproduced with permission.^[^ ^122^ ^]^ Copyright 2018, Wiley‐VCH.

Laponite (Na_0.7_[(Si_8_Mg_5.5_Li_0.3_)O_20_(OH)_4_]) is a synthetic Mg–Li phyllosilicate that belongs to the smectite family.^[^ ^118^, ^119^ ^]^ The laponite unit cell consists of six octahedral magnesium oxides sandwiched between two layers of four tetrahedral silicon oxides (**Figure** **25**a).^[^ ^120^ ^]^ In the powdered state, laponite crystals form highly oriented packed platelets by sharing interlayer sodium ions (Na^+^).^[^ ^85^, ^118^, ^119^ ^]^ In its XRD pattern, laponite shows three distinct peaks at 2*θ* values of 4.4°, 20.0°, and 35.0°, corresponding to the (001), (100), and (110) crystal planes, respectively (Figure 25b).^[^ ^121^ ^]^ SEM imaging has shown that laponite particles exhibit an irregular shape with an average diameter of ≈20 µm, indicating that layered disks of laponite can stack to form micron‐sized particles in dry powder form (Figure 25c).^[^ ^121^ ^]^ The layered shape of laponite has been corroborated by TEM imaging, which revealed its lamellar structure (Figure 25d).^[^ ^122^ ^]^ Specifically, laponite exhibits a high specific surface area (350 m^2^ g^−1^), aspect ratio (25), and discoid single crystals. Additionally, laponite is highly modifiable through the broken edges of the octahedral and tetrahedral sheets, which contain accessible hydroxyl groups that are available for silanation. Additionally, laponite contains exchangeable cations between the tetrahedral layers, which can balance the negative charges on its faces.^[^ ^123^ ^]^

In the laponite structure, the Mg and Si ions in the polyhedral sheets complement their coordination by bridging OH groups and O atoms, both of which can effectively trap polysulfides in Li–S batteries. Furthermore, laponite can facilitate Li‐ion transport upon the partial replacement of Mg^2+^ in the octahedral sheets by Li^+^. To exploit these properties, Yang and Zhang deposited a suspension containing laponite‐RD nanosheets (LNSs) and CB on one side of a commercial PP separator via vacuum filtration to fabricate an LNS/CB‐Celgard separator for Li–S batteries.^[^ ^85^ ^]^ CB served as an upper current collector and provided abundant electron pathways that could not be attained from the electronically nonconductive LNS alone. The fabricated separator exhibited remarkable flexibility owing to the large contact area with the Celgard separator. Furthermore, some of the LNSs in the LNS/CB layer were trapped in the pores of the Celgard separator, thereby strengthening the bonds between the LNS/CB layer and the separator and preventing exfoliation, even when folded or bent. Moreover, the ≈3.5‐µm‐thick LNS/CB layer was more uniformly porous than the Celgard separator. Additionally, the LNS/CB layer was adept at preventing polysulfide shuttling in Li–S batteries. In the case of the LNS/CB‐Celgard separator, the O active sites in the LNSs formed Li–O and O–S bonds with polysulfides, inhibiting polysulfide shuttling and significantly curbing the self‐discharge feature. Additionally, the nanolayered structure and intrinsic hydrophilicity of the LNSs and reutilization of the captured active materials through the conductive LNS/CB layer significantly improved the performance and properties of the battery, leading to high Li^+^ conductivity, excellent wettability, and enhanced thermal stability. When applied to Li–S batteries, the LNS/CB‐Celgard separator showed higher rate performance and cycling stability than those of the CB‐coated Celgard separator or Celgard separator. Furthermore, the separator could be manufactured using a simple tape‐casting method with PVDF, indicating its potential for achieving excellent performance in various battery types beyond Li–S batteries.

**Figure 26:**
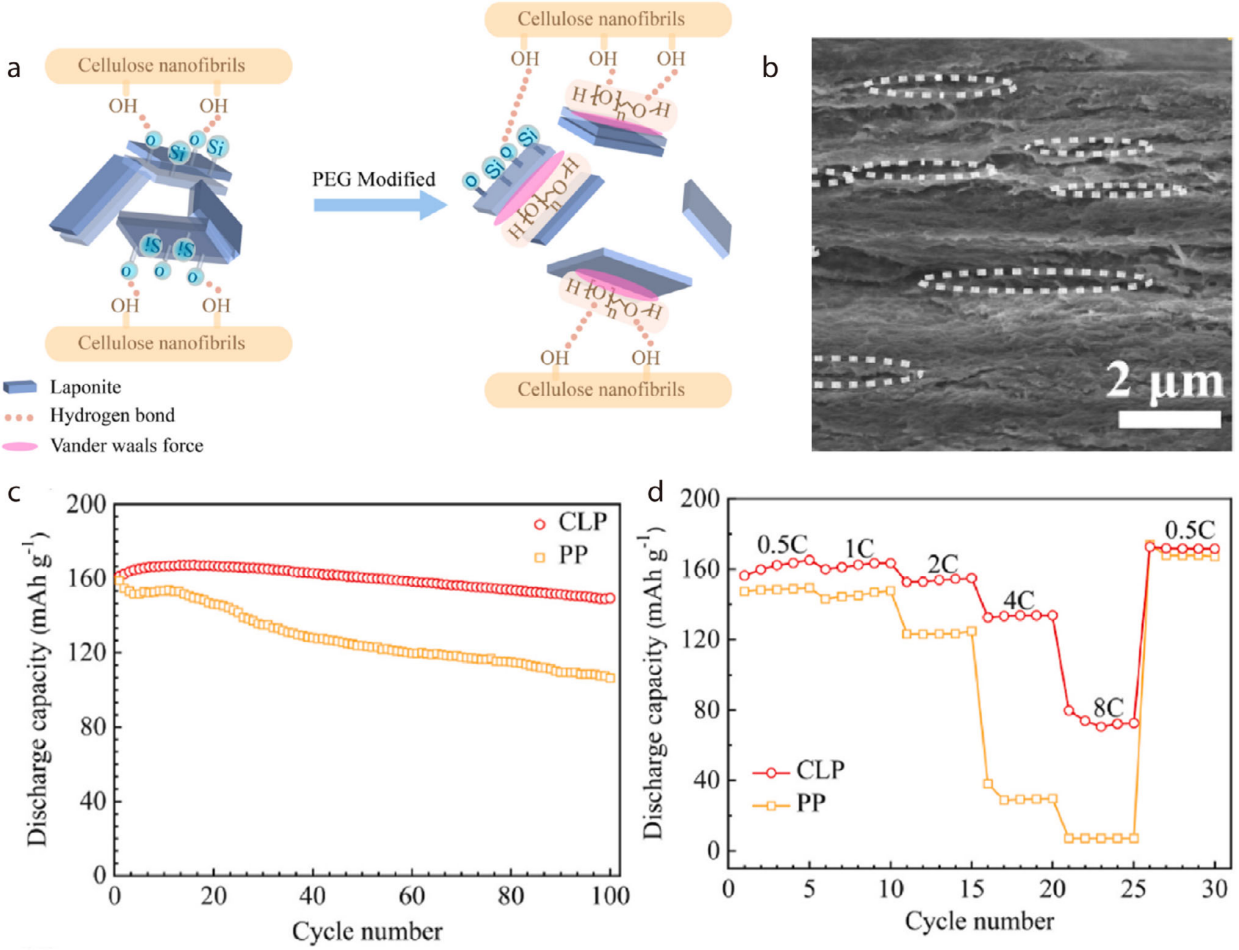
a) Schematic illustrating the distribution of inorganic laponite nanoparticles in a hybrid sol and their interactions with cellulose nanofibrils (CNFs). b) Cross‐sectional FE‐SEM image of CNF/PEG‐modified laponite (CLP) separator magnified 20 000×. c,d) Cycling performance and C‐rate capability of cells assembled with c) CLP separator or d) PP separator. Reproduced with permission.^[^ ^86^ ^]^ Copyright 2021, Elsevier.

In addition to Li–S batteries, LIBs can be incorporated with laponite to enhance the electrochemical properties of the separator. For example, Wang et al. fabricated a composite membrane of nacre‐like cellulose nanofibrils (CNFs) and laponite for LIBs using a sol–gel process (**Figure** **26**a).^[^ ^86^ ^]^ To that end, a laponite solution modified using polyethylene glycol (PEG) was mixed with the CNF solution and dried. Subsequently, PEG was removed using an ethanol bath, yielding the CNF/PEG‐modified laponite (CLP) separator. The fabricated separator exhibited excellent thermal stability, mechanical strength, electrochemical stability, and ionic conductivity. PEG helped regulate the CNF–laponite interface and develop a porous structure upon being removed. The hydrogen bonding between the CNFs and PEG improved the interfacial compatibility between the CNFs and laponite, resulting in a homogeneous distribution, which led to a unique porous structure when the uniformly dispersed PEG was removed. Consequently, the CLP separator contained uniform layers of sheets and a nacre‐like architecture with a narrow pore‐size distribution and an average pore size of 67 nm, which enhanced its mechanical properties and ionic conductivity. Moreover, numerous 100–200‐nm‐wide gaps acted as electrolyte reservoirs, enhancing the electrochemical performance (Figure 26b). Remarkably, the CLP separator exhibited a lower porosity (68%) and electrolyte uptake (260%) than those of the CNF/PEG (CP) separator but showed a higher ionic conductivity (0.977 mS cm^−1^). The cycling performance of the CLP separator was evaluated using an LIB with NCM811 as the cathode and Li metal as the anode (Figure 26c). The cell assembled with the CLP separator exhibited a high‐coulombic efficiency of 99.7% in the first cycle and formed a stable SEI layer. Furthermore, after 100 cycles, the PP separator retained only 66.9% of its initial discharge capacity, whereas the CLP separator exhibited an improved cycling performance of 92.6%. Subsequent rate performance tests (Figure 26d) showed that at all discharge rates, the cell with the CLP separator exhibited a substantially higher capacity than that of the cell with the PP separator. At 8.0C, the cell with the CLP separator showed a considerably higher capacity retention (45.7%) than that of the cell with the PP separator (4.8%).

For similar applications, laponite has been introduced together with other minerals. For example, Xu et al. fabricated organic–inorganic composite separators by introducing different amounts of vermiculite (VMT) and laponite into a PVDF matrix using the phase inversion method.^[^ ^87^ ^]^ The synergistic effects of the two inorganic nanofillers were explored. The incorporation of VMT and laponite effectively reduced the thermal shrinkage rate and crystallinity of PVDF and enhanced its thermal dimensional stability, porosity, and electrolyte uptake. Notably, the addition of equal amounts of VMT and laponite (PVDF/V5/L5) resulted in a high ionic conductivity and low interfacial impedance. However, the addition of these two types of clay nanoparticles did not appreciably improve the porosity and electrolyte uptake. Nevertheless, the PVDF separator with VMT (PVDF/V10) and another with laponite (PVDF/L10) exhibited slightly higher porosity and electrolyte uptake than those of PVDF/V5/L5. In particular, PVDF/L10 exhibited the smallest contact angle among the specimens, indicating that only one type of inorganic nanofiller had a strong affinity for the electrolyte. Laponite helped in reducing the pore size and making the pore distribution uniform because laponite with small particles and high dispersibility exhibited spatial complementarity in the organic matrix. When the PVDF/V5/L5 separator was applied to LIBs, a high discharge capacity, high coulombic efficiency, and remarkable capacity retention (98.4%) were achieved.

#### 2.2.7 Montmorillonite

**Figure 27:**
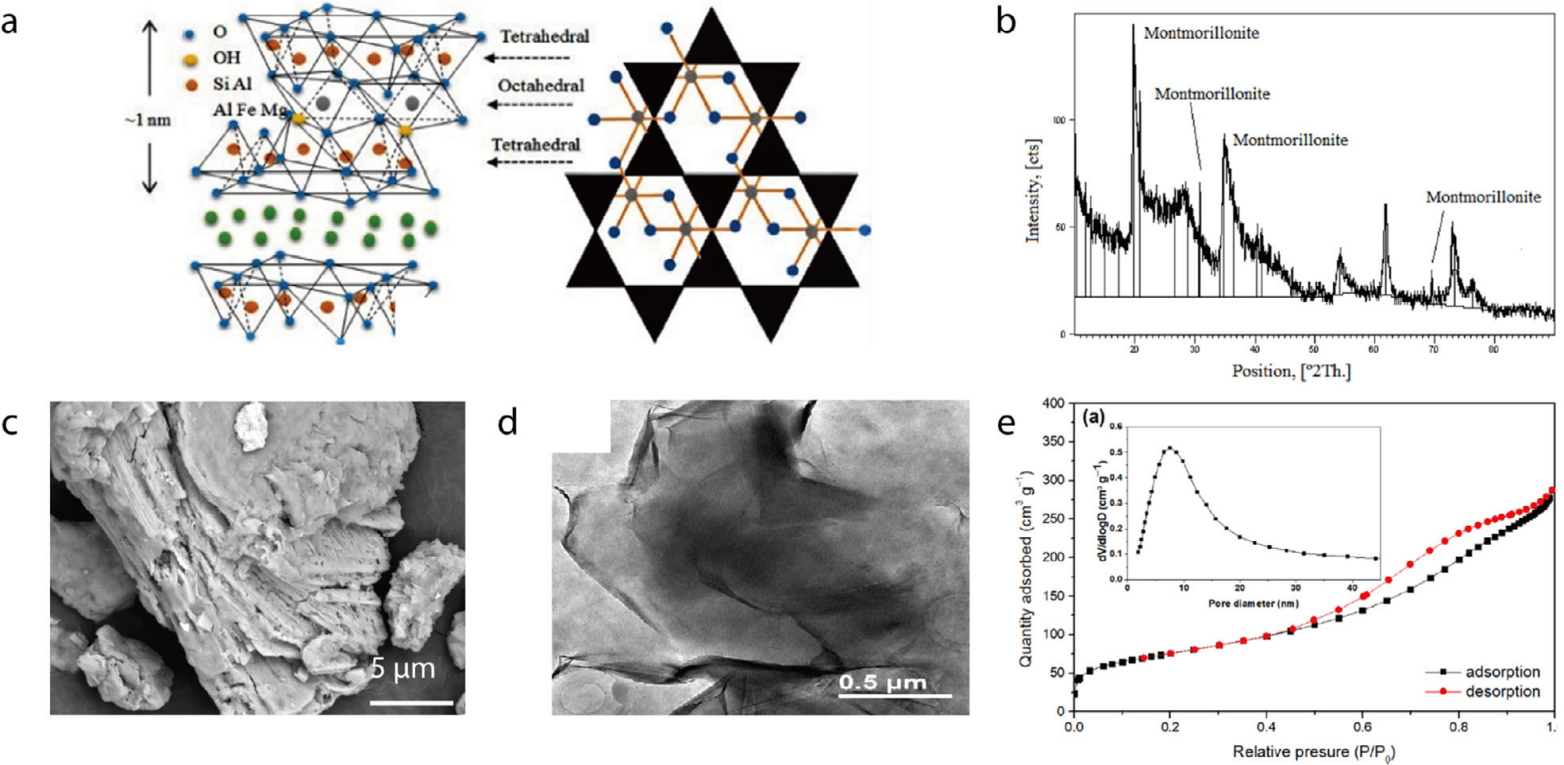
a) Layered structure of MMT. b) XRD pattern. Reproduced with permission.^[^ ^124^ ^]^ Copyright 2022, Elsevier. c) SEM image of MMT. Reproduced with permission.^[^ ^125^ ^]^ Copyright 2020, Multidisciplinary Digital Publishing Institute. d) TEM image of MMT clay. e) N_2_ adsorption–desorption isotherms of MMT, with corresponding pore‐size distribution (inset). Reproduced with permission.^[^ ^124^ ^]^ Copyright 2022, Elsevier.

Montmorillonite (MMT; (Na, Ca)_0.33_(Al, Mg)_2_(Si_4_O_10_)(OH)_2_•n(H_2_O)) belongs to the phyllosilicate group of minerals. It has a nanolayered structure in which a central octahedral alumina sheet (O–Al(Mg)–O) is sandwiched between two tetrahedral silicate sheets (O–Si–O) (**Figure** **27**a).^[^ ^124^ ^]^ The layers are approximately 100 nm long, 100 nm wide, and 1 nm thick. The layers are negatively charged owing to the isomorphous substitution of Al^3+^ by Mg^2+^ in the octahedral sheets, leading to the presence of cations such as Na^+^, Mg^2+^, and Ca^2+^ in the interlayer space of MMT. The crystallographic parameters of MMT have been evaluated based on the (001) and (080) XRD peaks (Figure 27b).^[^ ^124^ ^]^ SEM and TEM images have shown lamellar structures typically observed in particles with sizes ranging from ≈20 to ≈30 µm (Figure 27c,d).^[^ ^124^, ^125^ ^]^ The lamellar structure of MMT enables it to have a large specific surface and thereby effectively adsorb liquids. For instance, according to analyses of adsorption–desorption isotherms and the Barrett–Joyner–Halenda (BJH) pore‐size distribution, MMT exhibits a high pore volume and surface area (0.423 cm^3^ g^−1^ and 258.108 m^2^ g^−1^, respectively) (Figure 27e).^[^ ^124^ ^]^ Owing to its adsorbent properties, MMT is widely used in the construction industry to prevent fluid leakage. Furthermore, MMT exhibits hydrophilic and flame‐retardant properties, making it a favorable nanofiller for the separator matrix. Notably, the phyllosilicate structure of MMT allows the insertion of other molecules into its interlayer gap. However, the uniform dispersion of MMT within the polymer matrix is difficult owing to its small interlayer spacing.^[^ ^94^ ^]^

The negatively charged layers in MMT allow Li‐ion transfer with a significantly lower diffusion energy barrier (0.155 eV) than that for a carbon‐based surface (0.293 eV).^[^ ^93^ ^]^ This rapid Li‐ion‐conducting characteristic has triggered extensive studies on the application of MMT in LIB separators. For example, Fang et al. prepared composite separators with different PVDF and MMT contents via electrospinning.^[^ ^88^ ^]^ The membranes exhibited multilayered 3D network structures with numerous randomly oriented continuous fibers. The composite membranes demonstrated higher electrolyte uptake than that of the PVDF membrane, which was attributed to their higher porosity, good affinity toward the electrolyte, and the highly active surface of MMT. When evaluating the thermal dimensional stability at 150 °C, the commercial Celgard PP membrane changed in color (white to transparent) and shape (round to oval). In contrast, the PVDF and composite membranes retained their shapes, although the sample area decreased. Among the pure PP membrane, Celgard PP membrane, and composite membranes with different PVDF/MMT proportions, the PVDF/MMT‐5% specimen exhibited a particularly high ionic conductivity (4.2 mS cm^−1^) and low interfacial resistance (97 Ω). When this composite membrane was incorporated into a Li/LiFePO_4_ cell, it demonstrated nearly 100% capacity retention after 50 cycles at 0.2C rate. Furthermore, when being charged and discharged at different current rates (0.2C–2C) for 10 cycles, the cell assembled with the PVDF/MMT‐5% composite membrane showed excellent capacity retention (≈96%; ≈138 mAh g^−1^), which was equivalent to the capacity of the PP separator at 0.2C. This indicated that the cell assembled with the PVDF/MMT‐5% composite membrane had a more stable cycling performance and higher capacity than that of the variant with the commercial Celgard PP membrane.

A similar study on LiFePO_4_/graphite full‐cell LIBs was conducted by Dyartanti et al., who fabricated polymer electrolyte membranes (PEMs) by modifying PVDF via phase inversion (that is, nonsolvent‐induced phase separation; NIPS) using 7 wt% polyvinylpyrrolidone (PVP) and 8 wt% MMT as the pore‐forming agent and filler, respectively.^[^ ^92^ ^]^ The additive‐modified membranes exhibited increased porosity, electrolyte uptake, and ionic conductivity. In the cycling performance assessment of LiFePO_4_/PEM/graphite batteries, the prepared PEMs with MMT exhibited a higher discharge capacity and coulombic efficiency than those of pure PVDF and Celgard, thus demonstrating excellent stability.

MMT‐based separators have also been fabricated and tested for LIBs with oxide cathodes. For instance, Para et al. employed the doctor blade technique to modify Celgard 2325 with two composites prepared using different MMT/polyaniline (PANI) ratios for LiNi_1.5_Mn_0.5_O_4_/graphite full cells.^[^ ^90^ ^]^ To prepare the composites with different structural arrangements, the MMT/PANI ratio was set to 87:13 or 12:88. In the MMT/PANI_87/13 composite, PANI was preferentially located in the interlamellar spaces of MMT, whereas in the MMT/PANI_12/88 specimen, the inorganic sheets were integrated in an amorphous polymeric matrix. Both samples contained 3D multilayered network structures. However, MMT/PANI_87/13 was an intercalated composite in which the polymer chains were present at regular distances between the inorganic clay layers, whereas MMT/PANI_12/88 was an exfoliated composite with a high percentage of PANI agglomerates on its surface. The coated separators demonstrated higher thermal resistance, electrolyte affinity, mechanical strength, and ionic conductivity than those of the bare separators. Notably, the LiNi_1.5_Mn_0.5_O_4_/graphite full‐cell assembled with MMT/PANI_87/13 showed optimal cycling performance, thus exhibiting low capacity fading and high cyclability.

Another study on MMT‐based separators for LIBs with oxide cathodes was conducted by Li et al., who fabricated intercalated organic MMT (OMMT)–polyimide (PI) separators for LiCoO_2_/Li‐metal batteries.^[^ ^94^ ^]^ Notably, a new method—solution blow spinning followed by thermal imidization—was proposed for low‐cost mass production. OMMT was obtained by intercalating cetyltrimethylammonium bromide into the interlayer spacing of MMT, which decreased the porosity while increasing the wettability and electrolyte uptake. This was due not only to the interconnected porous structure that allowed OMMT to accommodate more electrolyte but also due to the exposed OMMT nanosilicate layer that induced good affinity between the PI/OMMT separators and the carbonate groups in the electrolyte. After heat treatment at 180 °C for 30 min, the system exhibited good thermal dimensional stability with no thermal shrinkage. Notably, in tensile tests, the hybrid separator showed a 102.6% improvement in mechanical strength over that of the pristine PI separator, with a corresponding maximum strength of 26.23 MPa for the separator containing 7 wt% OMMT. This improvement was attributed to the good interfacial adhesion between the well‐dispersed OMMT filler and the polymer, enabling the filler to make the polymer matrix both rigid and flexible. However, as the OMMT content increased further, both the tensile strength and elongation decreased owing to the formation of OMMT agglomerates and stress concentration defects. Likewise, the highest ionic conductivity was also exhibited by the separator with 7 wt% OMMT. When applied to LiCoO_2_/Li‐metal batteries, the PI/7wt%‐OMMT hybrid separator showed better cycling stability and rate capability than those of the cells assembled with pristine PI or Celgard 2400 separators. Overall, the proposed environmentally friendly, convenient fabrication method yielded an LIB separator with improved performance as well as showed promise for industrial‐scale utilization.

**Figure 28:**
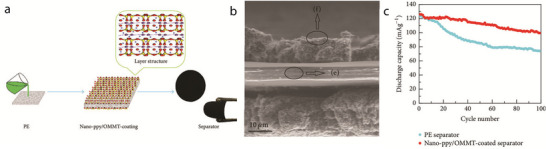
a) Schematic illustrating preparation of nano‐ppy/OMMT‐coated separator. b) Cross‐sectional structure of nano‐ppy/OMMT‐coated separator. c) Charge–discharge curves of cells assembled with PE separator or nano‐ppy/OMMT‐coated separator after 100 cycles at 80 °C. Reproduced with permission.^[^ ^95^ ^]^ Copyright 2017, Wiley‐VCH.

Similarly, Yang et al. coated both sides of a PE separator with nanopolypyrrole (nano‐ppy)/OMMT to develop high‐performance separators for LiNi_1/3_Co_1/3_Mn_1/3_O_2_/Li‐metal batteries. OMMT was obtained via cation exchange between the Al^3+^ in MMT and octadecyl dimethylammonium chloride (ODAC) (**Figure** **28**a).^[^ ^95^ ^]^ The long alkyl chain of ODAC increased the interlayer spacing of MMT, allowing nano‐ppy to readily enter the MMT layers. The nano‐ppy/OMMT‐coated separator exhibited a considerably higher onset decomposition temperature than that of the PE separator. This was because the restricted chain motion of the coated separator required higher thermal energy to initiate chain scission, and the exfoliated and well‐dispersed nano‐ppy/OMMT acted as an insulating surface, hindering the outward diffusion of the decomposed products. Consequently, the composite separator withstood decomposition at elevated temperatures of up to 255 °C, which is the decomposition temperature of ppy. These results indicated that the composite separator could be used in LIBs at high temperatures. Furthermore, the nano‐ppy/OMMT‐coated separator with a high specific surface area and a 3D multilayered network structure exhibited an organic electrolyte uptake that was more than two times higher than that of the PE separator, enhanced ionic conductivity, and excellent mechanical properties (Figure 28b). The cycling performance of a LiNi_1/3_Co_1/3_Mn_1/3_O_2_/Li‐metal battery assembled with the nano‐ppy/OMMT‐coated separator was evaluated at 0.5C after 100 cycles at 80 °C. The initial discharge capacity of the battery (125.9 mAh g^−1^) decreased slightly to 99.12 mAh g^−1^ after 100 cycles, which was ≈80% of the initial discharge capacity. In contrast, the cell with the PE separator exhibited an initial discharge capacity of 123.7 mAh g^−1^, which sharply dropped to 74.1 mAh g^−1^ after 100 cycles, thereby losing over 40% of its initial capacity. These results indicated that coating the surface of PE separators with nano‐ppy/OMMT could reduce the loss of discharge capacity at high temperatures (Figure 28c).

A notable feature of MMT is the numerous Lewis active sites that can anchor polysulfides migrating in their vicinity. Therefore, when MMT is used to coat separators, it can prevent the shuttle effect in Li–S batteries and significantly improve their cyclability. For example, Wang et al. fabricated a Se_0.06_SPAN/MMT@PP separator by doctor‐blade casting using MMT and selenium‐doped sulfurized PAN (Se_0.06_SPAN) for Li–S batteries.^[^ ^91^ ^]^ Doping Se into SPAN enhanced its electrical and ionic conductivities and catalytic activity for polysulfide conversion. The Se_0.06_SPAN/MMT@PP composite was prepared by in situ polymerization, followed by selenization and sulfurization, in a 1:1 ratio. The composite exhibited high wettability and excellent mechanical, chemical, and electrochemical stabilities. The layered structure of MMT effectively anchored the dissolved polysulfides and provided sufficient channels for Li‐ion transport. Additionally, Se_0.06_SPAN supported on MMT accelerated the conversion of immobilized polysulfides and activated “dead sulfur” by lowering the potential barrier for transforming insoluble Li_2_S into soluble Li_2_S_x_. Its reaction product with Li also catalyzed the conversion of polysulfides. Interestingly, the measured discharge capacity (1759 mAh g^−1^) exceeded the theoretical specific capacity of the sulfur cathode (1675 mAh g^−1^). Furthermore, the specimen exhibited an ultrahigh areal capacity (33.07 mAh cm^−2^) under high sulfur loading (26.75 mg cm^−2^), showed low Li excess (ratio of negative to positive (N/P) = 3.2), and allowed lean electrolyte use (4.5 µL mg^−1^). To simulate its practical applications, the developed separator was assembled into a large‐scale pouch cell with dimensions of 4.3 × 3.4 cm. The cell showed a discharge capacity of 741 mAh g^−1^ after 20 cycles under lean‐electrolyte conditions (8 µL mg^−1^), thus demonstrating significant potential in practical applications of high‐capacity Li–S batteries.

Another notable example of MMT‐based separators for Li–S batteries was reported by Yang et al., who employed an interfacial engineering approach to coat polypyrrole (ppy)‐modified Li‐MMT (ppy/Li‐MMT) onto a PP separator.^[^ ^93^ ^]^ Raw MMT was first converted to intermediate hydrogen‐ion MMT (H‐MMT) using 0.5 m H_2_SO_4_ and then to Li‐ion MMT (Li‐MMT) using 1 m LiOH solvent, followed by collection through freeze‐drying. The ppy/Li‐MMT@PP powders were produced by polymerizing pyrrole monomers on the Li‐MMT surface. Subsequently, the ppy/Li‐MMT@PP separator was fabricated by pouring and coating a slurry (ppy/Li‐MMT and PVDF mixed in a mass ratio of 9:1) onto the PP separator. The strong adsorption properties and excellent electrical conductivity of the interface‐engineered separator enabled rapid conversion of long‐chain polysulfides to short‐chain polysulfides, resulting in a high discharge capacity. Moreover, compared to those of the PP separators, the wettability and electrolyte uptake (348.6%) were considerably improved, resulting in a high Li‐ion conductivity (3.63 mS cm^−1^). The ppy/Li‐MMT@PP separator did not show thermal shrinkage at a high temperature of 150 °C and exhibited stable battery performance even at an internal temperature of 80 °C. Furthermore, it could not be punctured during the hot metal rod piercing test conducted at 130 °C. ppy/Li‐MMT exhibited excellent thermal stability because it absorbed heat energy uniformly. Additionally, when assembled with a sulfur cathode, ppy/Li‐MMT exhibited stable cycling performance over 600 cycles and capacity retention.

In addition to Li^+^, other cations can replace the cations in the MMT layers by submerging MMT in an alkaline solution, thus enhancing the conductivity of other cations. For example, Chen et al. coated MMTs onto a polyethersulfone (PES) substrate via spray coating for alkaline zinc–iron flow batteries (AZIFBs).^[^ ^89^ ^]^ The PES membrane (PS‐M) was fabricated by phase inversion using minimal amounts of SPEEK. The prepared composite membrane (MMT‐M) mechanically outperformed the PS‐M and Nafion115 membranes. The high mechanical strength of MMT prevented damage caused via the dendritic growth of metallic zinc. Additionally, the negatively charged properties of MMT effectively equilibrated the distribution of zincate ions at the membrane–electrode interface. Furthermore, through spray‐coating, ≈5‐µm‐thick MMT layers were stacked in an orderly manner. The AZIFB incorporated with MMT‐M exhibited a high coulombic efficiency and voltage efficiency, along with improved cycling stability and stable battery performance compared with those of the battery with PS‐M. After the cycling tests, the batteries were disassembled to investigate the morphology of the Zn deposited on the negative electrode. The batteries with PS‐M showed a “honeycomb‐like” morphology, whereas those with MMT‐M showed a compact and flat “stick‐like” morphology, indicating that MMT‐M effectively protected the membrane from Zn dendrite damage.

#### 2.2.8 Vermiculite

**Figure 29:**
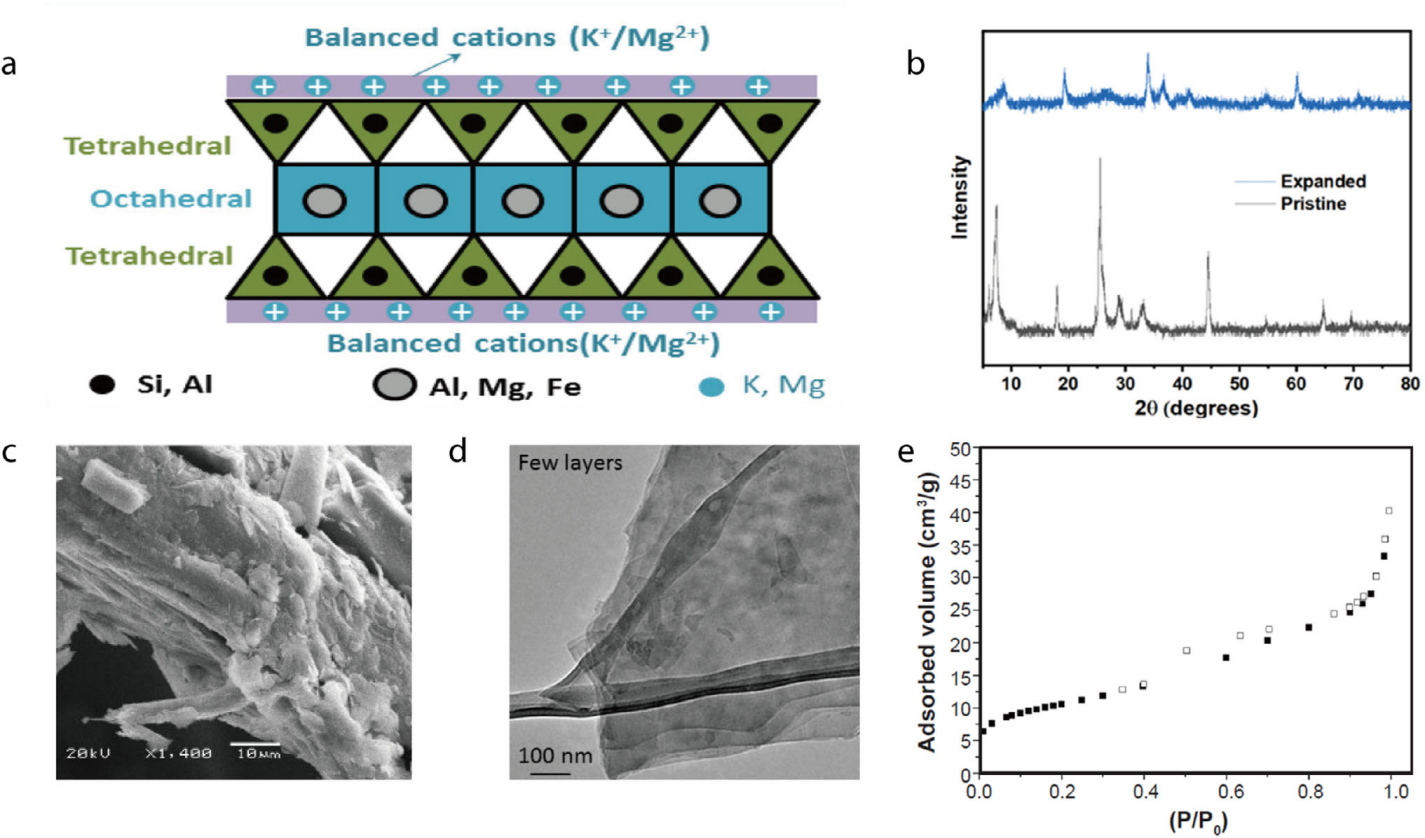
a) Structure of VMT. Reproduced with permission.^[^ ^127^ ^]^ Copyright 2019, Wiley‐VCH. b) XRD patterns of VMT ore and expanded VMT. Reproduced with permission.^[^ ^96^ ^]^ Copyright 2021, Elsevier. c) SEM image of raw VMT. Reproduced with permission.^[^ ^129^ ^]^ Copyright 2008, Elsevier. d) TEM image of bare VMT. Reproduced with permission.^[^ ^127^ ^]^ Copyright 2019, Wiley‐VCH.) e) N_2_ adsorption (filled square) and desorption (open square) isotherms of raw VMT. Reproduced with permission.^[^ ^130^ ^]^ Copyright 2021, SciELO.

VMT is formed in nature through the hydration of phlogopite and magnesium–iron biotite and is abundantly present in countries such as the USA, Russia, South Africa, and Brazil.^[^ ^126^ ^]^ At the atomic scale, VMT exists as a mica‐type trioctahedral silicate with the molecular formula (OH)_4_(Mg.Ca)_x_(Si_8‐x_Al_x_)(Mg.Fe)_6_O_20_•yH_2_O. Specifically, Mg and Ca cations surrounded by water molecules are located in the interlayer spaces, whereas Mg and Fe cations are present in the octahedral layer, with (Si_8‐x_Al_x_) atoms being in the tetrahedral layer (**Figure** **29**a).^[^ ^127^ ^]^ VMT exhibits a lamellar sandwich structure containing a sheet of magnesia (Mg–O) octahedra between two sheets of SiO_4_ tetrahedra.^[^ ^87^, ^99^ ^]^ The interlayer between the parallel layers is weakly bound by water molecules, compensating for the positive charge deficiency from exchangeable cations such as Ca^2+^, Mg^2+^, Na^+^, K^+^, and Al^3+^ in the tetrahedral layers. VMT shows excellent promise for use in nanocomposites after undergoing treatments such as mechanical grinding, ion exchange exfoliation, modification, and thermal shock.^[^ ^87^ ^]^ In particular, when subjected to heat treatment, VMT becomes resistant to rotting, while being able to absorb and retain liquids and gases, rendering it suitable for use in various industries.^[^ ^126^ ^]^ In its XRD pattern, VMT exhibits a characteristic peak at a 2*θ* value of ≈8°, corresponding to the (002) plane with *d*_002_ = 14.4 Å.^[^ ^128^ ^]^ This XRD peak can shift when other molecules are intercalated into VMT to expand its interlayer spacing (Figure 29b).^[^ ^96^ ^]^ At the microscopic level, raw VMT has been observed to exhibit a multilayered structure (Figure 29c),^[^ ^129^ ^]^ with TEM imaging revealing a nanosheet configuration (Figure 29d).^[^ ^127^ ^]^ Additionally, the adsorption isotherm of raw VMT is similar to the Type II curve established according to Braunauer–Emmett–Teller theory (Figure 29e).^[^ ^130^ ^]^

Similar to other 2D minerals, VMT exhibits improved mechanical and thermal stability and can be coated onto separators to enhance their properties. For instance, Carter et al. cast a 7‐µm‐thick layer of 1:9 VMT/PVDF onto PP using a doctor blade.^[^ ^96^ ^]^ The resulting specimen exhibited enhanced electrolyte uptake, electrode reaction kinetics, and stability without participating in redox processes. Moreover, the results showed lesser exothermic heat release and a higher melting temperature during thermal runaway than those of the cell assembled with the pristine PP separator.

Zhai et al. prepared multiscale‐structured PVDF/PAN/VMT nanosheet (VN) fibrous membranes for LMBs via one‐step electrospinning.^[^ ^98^ ^]^ The membranes exhibited enhanced ionic conductivity, wettability, thermal stability, and tensile strength. Additionally, they showed a reduced average pore size and uniform pore‐size distribution, promoting a more homogeneous Li^+^ flux distribution at the separator–electrode interface, thereby mitigating the formation and growth of Li dendrites. When applied to Li/Li_4_Ti_5_O_12_ cells, the membrane exhibited a better rate capability and longer Li plating life than those of a cell with a Celgard membrane.

Similarly, Yang et al. applied different amounts of VNs to PVDF separators via NIPS to develop high‐performance LIBs.^[^ ^99^ ^]^ The introduction of VNs reduced the crystallinity of PVDF and increased both the melting temperature *T*_m_ and crystallization temperature *T*_c_, thereby improving its thermal stability. In particular, the tensile strength and Young's modulus increased significantly owing to the hydrogen bonding between the PVDF matrix and the hydroxyl groups on the VNs. The incorporation of 2D VNs resulted in the creation of large finger‐like pore structures and increased the porosity, electrolyte uptake rate, and ionic conductivity and improved the electrolyte affinity and interface stability, thereby enhancing the electrochemical stability by protecting the PVDF separators from adverse reactions. When the composite film with 7.0 wt% VNs was assembled in a cell with LiFePO_4_ as the positive active material, the resulting system exhibited better rate capability, cyclability, and a wider electrochemical window than those of a cell with the commercial separator.

**Figure 30:**
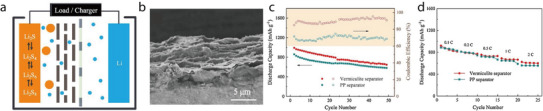
a) Schematic of exfoliated VMT separator in Li‐S battery, which can effectively repel the polysulfide anions and mitigate the “shuttle effect”. b) SEM image of VMT separator. c) Long‐term cycling performance at 0.1C. d) Rate performance of Li–S batteries with VMT‐based or conventional PP separators between 0.1C and 2C. Reproduced with permission.^[^ ^97^ ^]^ Copyright 2017, Elsevier.

Another interesting characteristic of VMT is its ability to repel polysulfide anions through electrostatic interactions. This phenomenon has been exploited to prevent polysulfides from penetrating the separator in Li–S batteries and improve their cycling stability. For example, Xu et al. fabricated a lamellar separator from exfoliated VMT for Li–S batteries (**Figure** **30**a).^[^ ^97^ ^]^ The VMT nanosheets were subjected to ion exchange in a LiCl solution to enrich the Li ions in the interlamellar structures of the VMT nanosheets. The dispersed VMT nanosheets were then reassembled into a freestanding membrane via vacuum filtration. The thickness was adjusted by controlling the amount of VMT dispersion to be filtered. The VMT silicate sheets arranged in a layer‐by‐layer manner were ≈10 µm thick and exhibited a densely stacked morphology (Figure 30b). The 2D exfoliated VMT sheets were negatively charged, which repelled polysulfide anions through electrostatic interactions and prevented the shuttle effect while allowing Li‐cation transport. Furthermore, the dense surface structure comprising rigid VMT sheets inhibited the growth of Li dendrites, thereby increasing battery safety. When applied to Li–S batteries, the cell assembled with the VMT‐coated separator exhibited a high initial specific capacity with an average coulombic efficiency of 90.3% for 50 cycles at a current rate of 0.1C. In contrast, the cell assembled with the PP separator exhibited a fluctuating coulombic efficiency of less than 70% and a considerably lower initial capacity (Figure 30c). However, a thick Li_2_S layer was formed as the discharge process continued, causing continuous capacity loss. The ion‐transfer resistance was investigated by evaluating the rate performance from 0.1C to 2C (Figure 30d). High discharge capacities of 700 and 600 mAh g^−1^ were obtained at rates of 1C and 2C, respectively.

#### 2.2.9 2D Mineral Summary

**Figure 31:**
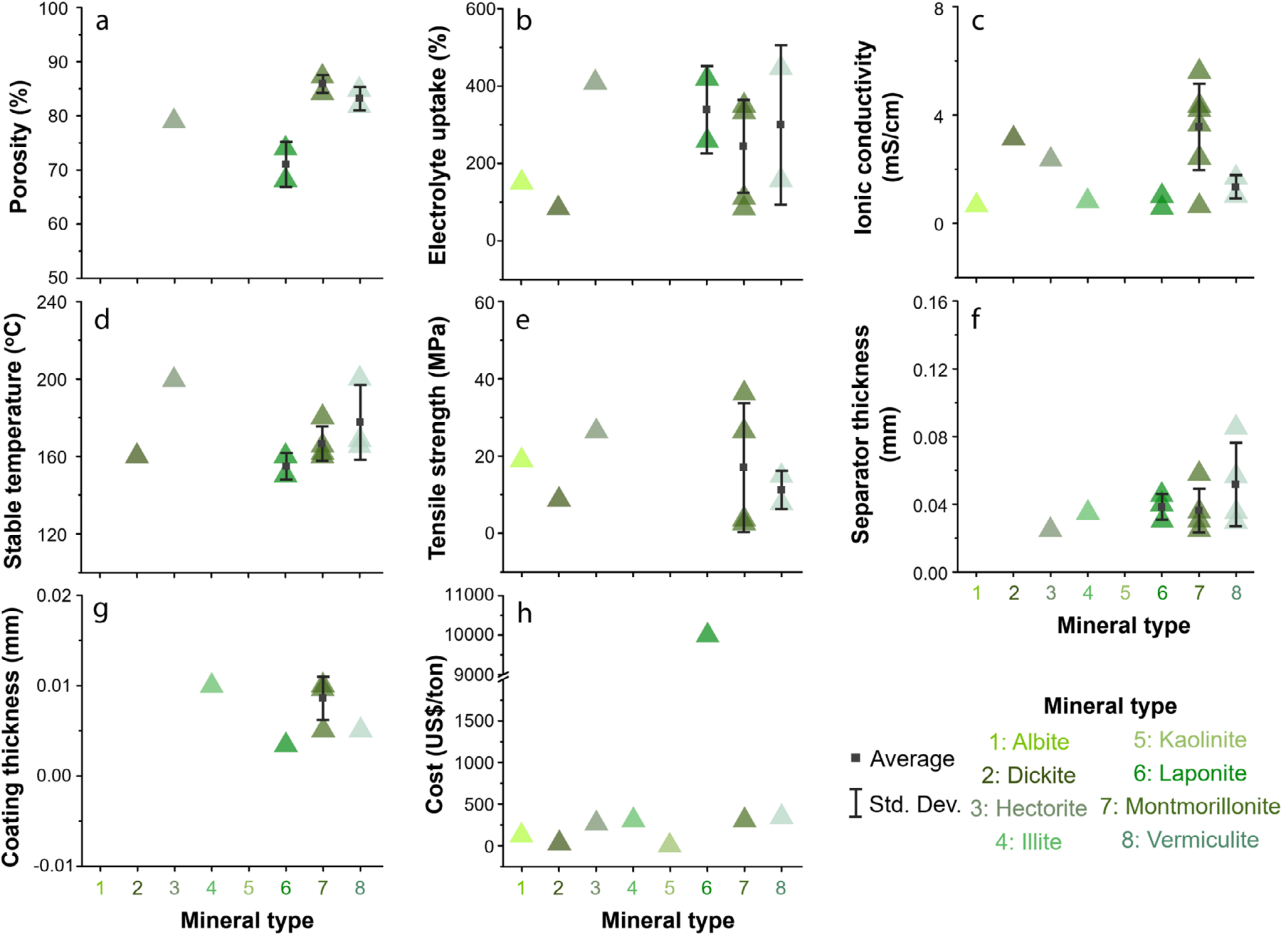
Dependence of separator parameters—a) porosity, b) electrolyte uptake, c) ionic conductivity, d) stable operating temperature, e) tensile strength, f) separator thickness, g) coating thickness, and h) cost—on the type of 2D mineral; Average and standard deviation of the data are indicated as black squares and vertical lines, respectively.

**Figure 32:**
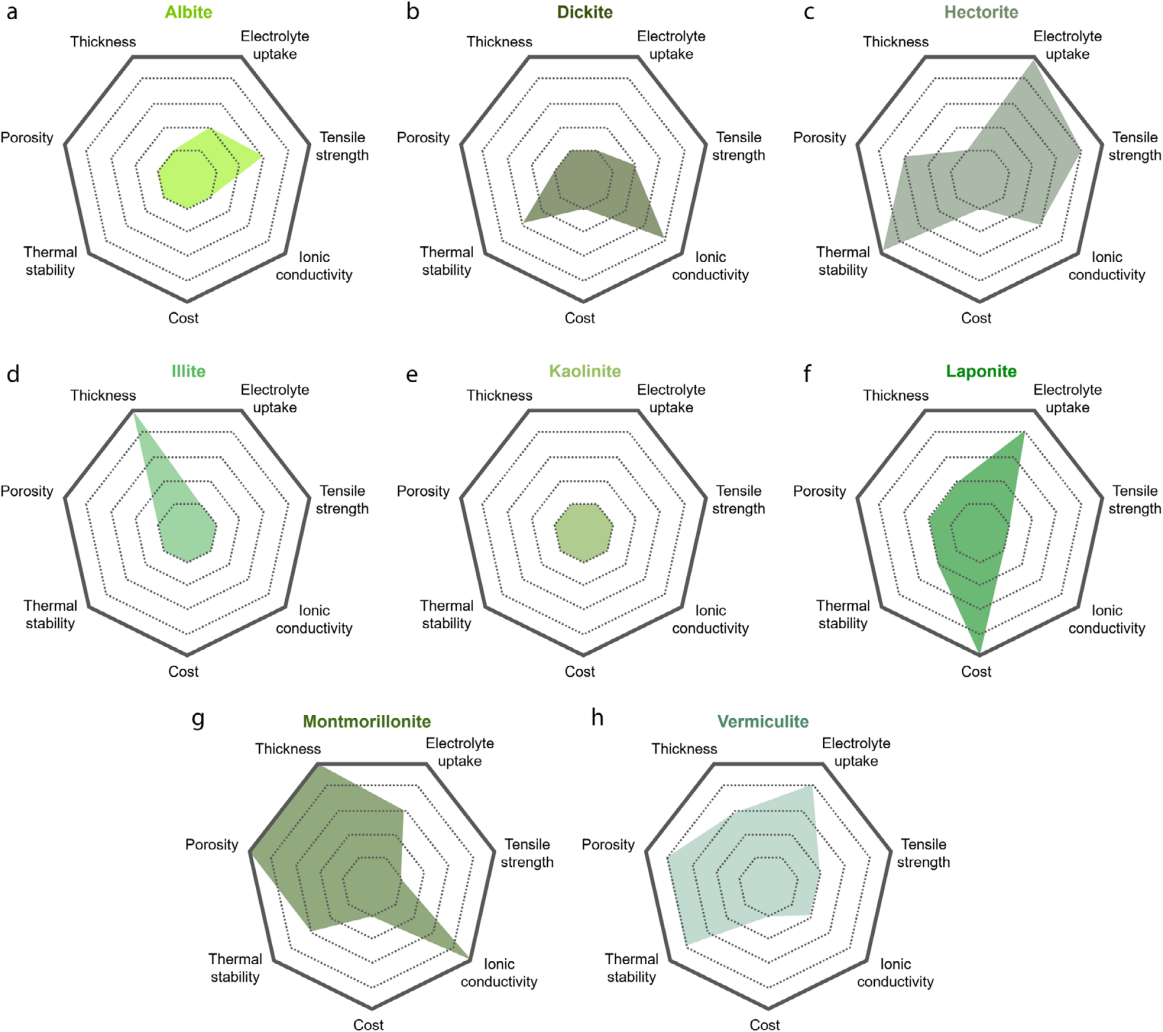
Radar plots for parameters of separators fabricated using different types of 2D minerals.

**Figures** **31** and **32** exhibit the comparison of performance indicators for 2D minerals reviewed in this study. Among 2D minerals, minerals that contain large hydrated cations between the layers, such as HT, MMT, and VMT, demonstrate high porosity, electrolyte uptake, and excellent ionic conductivity. All four 2D minerals discussed here—dickite, HT, MMT, and VMT—also demonstrate high thermal stability. This is attributed to their common structural basis on SiO_4_ tetrahedra, which contain strong Si–O bonds that are resistant to chemical decomposition upon heating. From a mechanical standpoint, albite and HT exhibit excellent tensile strength, thanks to their oxide skeletal framework and octahedral layers, respectively. From an economic perspective, laponite is significantly more expensive than other minerals, yet its performance does not appear to justify the cost. In contrast, KL is the most affordable (≈5 US$ ton^−1^)^[^ ^131^ ^]^ but underperforms in nearly all performance metrics. Notably, dickite and albite offer moderate to good performance at low cost. Dickite is the second most affordable 2D mineral (9–30 US$ ton^−1^)^[^ ^132^ ^]^ while still offering excellent ionic conductivity and decent mechanical and thermal stability. Albite also shows good mechanical strength at a relatively low cost (110 US$ ton^−1^).^[^ ^133^ ^]^ Taking both cost and performance into account, HT stands out as a highly promising 2D mineral. While MMT and VMT are also attractive materials in terms of conductivity, their poor mechanical strength suggests that they should be blended with stronger minerals such as albite or HT for practical use.

### 2.3 Bulk and/or Porous (3D) Minerals

**Figure 33:**
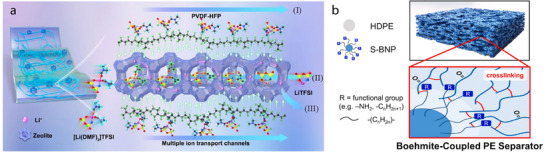
a) Exfoliated zeolite cages embedded in a PVDF‐*co*‐hexafluoropropylene (PVDF‑HFP)/LiTFSI matrix form a rigid 3D network of interconnected Li⁺ transport channels. Reproduced with permission.^[^ ^144^ ^]^ Copyright 2025, Elsevier. b) Sulfated boehmite nanoparticles crosslinked within a high‐density PE (HDPE) matrix create a 3D scaffold that enhances mechanical strength and interfacial adhesion. Reproduced with permission.^[^ ^145^ ^]^ Copyright 2023, Elsevier.

Unlike 1D and 2D minerals, 3D minerals have multiple or uncountable preferential bond cleavage directions, resulting in the formation of polyhedral particles or even porous structures. For instance, boehmite—a periodic crystal with three elements (Al, O, and H)—has three families of bond cleavage directions ({010}, {100}, and {101}), with the surface energy of these planes depending on the pH of the reaction medium.^[^ ^143^ ^]^ When the pH of the reaction medium is near 7, which is the most common coating condition for separators, interatomic bond breakage along the aforementioned directions leads to the formation of nearly rectangular pellets. Another representative 3D mineral is diatomite, which is formed by the fossilization of hard‐shelled microalgae. Owing to the diversity of microalgae, fossilized diatomite can exhibit various structural morphologies that can be roughly categorized into disk and linear configurations. Despite their morphological diversity, most diatomite specimens exhibit a porous structure. Another representative 3D mineral is zeolite, which can exhibit more than 200 types of 3D‐networked structures through complex combinations of corner‐sharing bonds between SiO_4_ and AlO_4_ tetrahedra. Depending on the manner in which the tetrahedra are connected, zeolites can have pores ranging in diameter from 0.3 to 1 nm, through which ions and molecules can migrate (**Figure** **33**a). Overall, compared with 1D or 2D minerals, 3D minerals have atomic frameworks that are strongly connected in multiple directions. This well‐developed multidirectional connectivity and inherent porosity significantly increase their interfacial contact and interactions with polymers and other surrounding materials (Figure 33b). Consequently, 3D minerals typically exhibit robust mechanical properties that are often represented by a high tensile strength.

**Figure 34:**
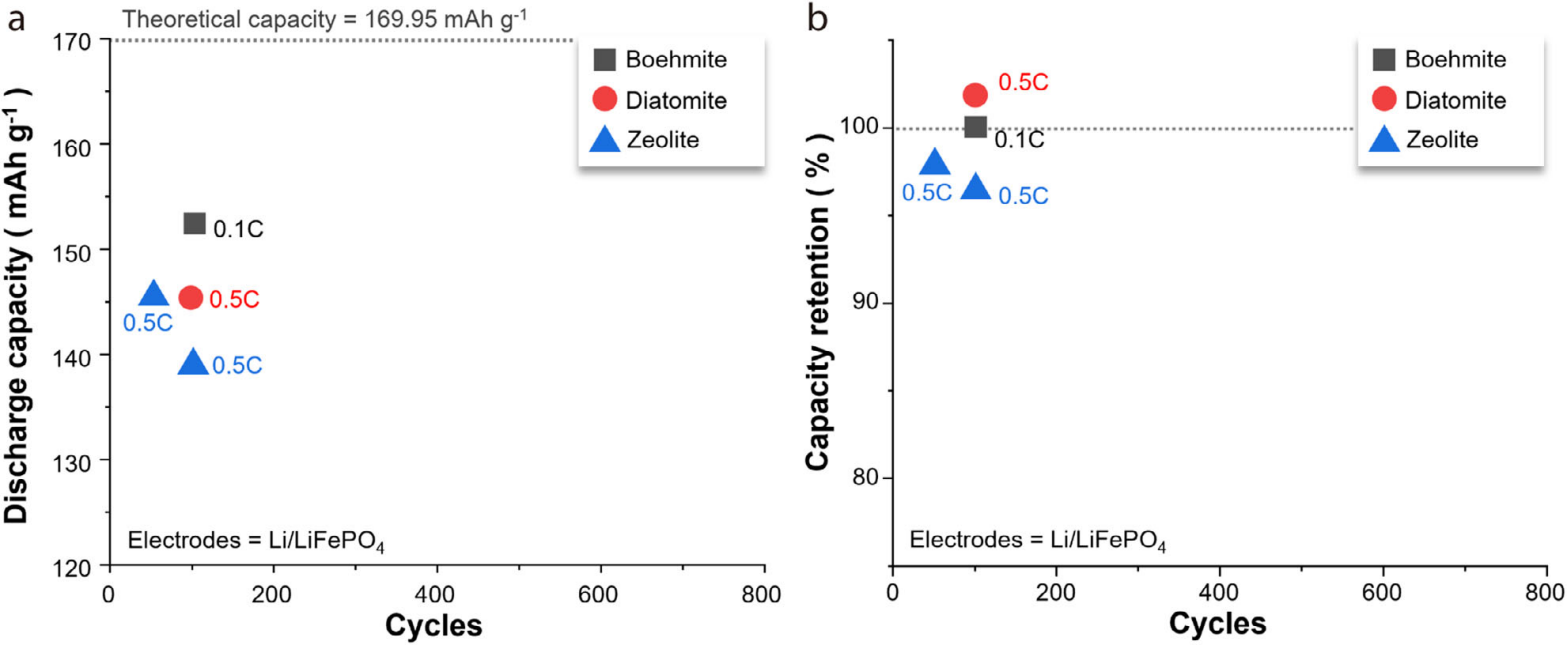
a) Discharge capacity–cycle number and b) capacity retention–cycle number plots for Li/LiFePO_4_ batteries with separators composed of bulk and/or porous minerals; The discharge capacity was estimated at the end of the cycling test, and the capacity retention was estimated as the ratio of the first and last discharge capacities. The C‐rate of each battery test is indicated next to the symbols.^[^ ^134^, ^136^, ^137^, ^141^ ^]^

The high mechanical strength of 3D minerals has triggered extensive studies on LIB separators fabricated using them (**Table** **3**). The superior mechanical properties of these separators significantly enhance the coulombic efficiency of LIBs. Among the various battery types, Li/LiFePO_4_ systems have been extensively used to test separators fabricated using 3D minerals; other cells have rarely been employed in this regard. A comparison of the performance of Li/LiFePO_4_ batteries containing 3D‐mineral‐coated separators (**Figure** **34**a) indicates that the discharge capacity in the last cycle is lower than that measured for batteries with 1D‐ or 2D‐mineral‐coated separators. This suggests that the separators with 3D minerals may not be suitable for developing high‐energy‐density batteries. In contrast, the use of 3D minerals remarkably improves the capacity retention of the batteries (Figure 34b), reaching values close to 100%, which suggests that coating with 3D minerals is an effective strategy for improving the long‐term cycling capability of batteries. The enhanced cyclability presumably emanates from the high mechanical and thermal stabilities of the 3D minerals, as discussed in the sections below.

**Table 3:** Performance of composite separators based on bulk and/or porous minerals.

Mineral	Host separator	Method	Electrode materials	Liquid electrolyte	Capacity retention (number of cycles)	C‐rate	Reference
Boehmite	PI	Coating	Li, LiFePO_4_	LiPF_6_, 1:1:1 EC/DEC/DMC	99% (100)	0.1C	[134]
	PE	Coating	Li, Li_4_Ti_5_O_12_	LiPF_6_, 1:1 EC/DEC	96.30% (100)	1C	[135]
Diatomite	PET	Coating	Li, LiFePO_4_	LiPF_6_, 1:1 EC/DEC	98.79% (100)	0.5C	[136]
Zeolite	PI	Coating	Li, LiFePO_4_	LiPF_6_, 1:1 EC/DMC	97.60% (50)	0.5C	[137]
	Celgard@2320	Coating	Graphite, NMC532+LMO	LiPF_6_, 1:1 EC/DEC	78.30% (500)	6C	[44]
	Celgard@2500	Coating	Li, LTO	LiPF_6_, 1:1:1 EC/DMC/DEC	99.00% (12)	0.2C	[138]
	PET	Coating	Li, LiFePO_4_	LiPF_6_, 1:1 EC/DEC	99.00% (100)	0.5C	[139]
	Celgard	Coating	Li, LiNi_0.8_Co_0.1_Mn_0.1_O_2_	LiPF_6_, 1:1 EC/DMC	95.45% (120)	0.1C	[140]
	Celgard@2400	Coating	Li, LiFePO_4_	LiPF_6_, 1:1:1 EC/DMC/DEC	96.20% (100)	0.5C	[141]
	Celgard	Coating	Li, S	LiTFSI (1 M) + LiNO_3_ (2.0%), Li_2_S_6_ (0.1 M), 1:1 DOL/DME	89.50% (100)	0.5C	[142]

#### 2.3.1 Boehmite

**Figure 35:**
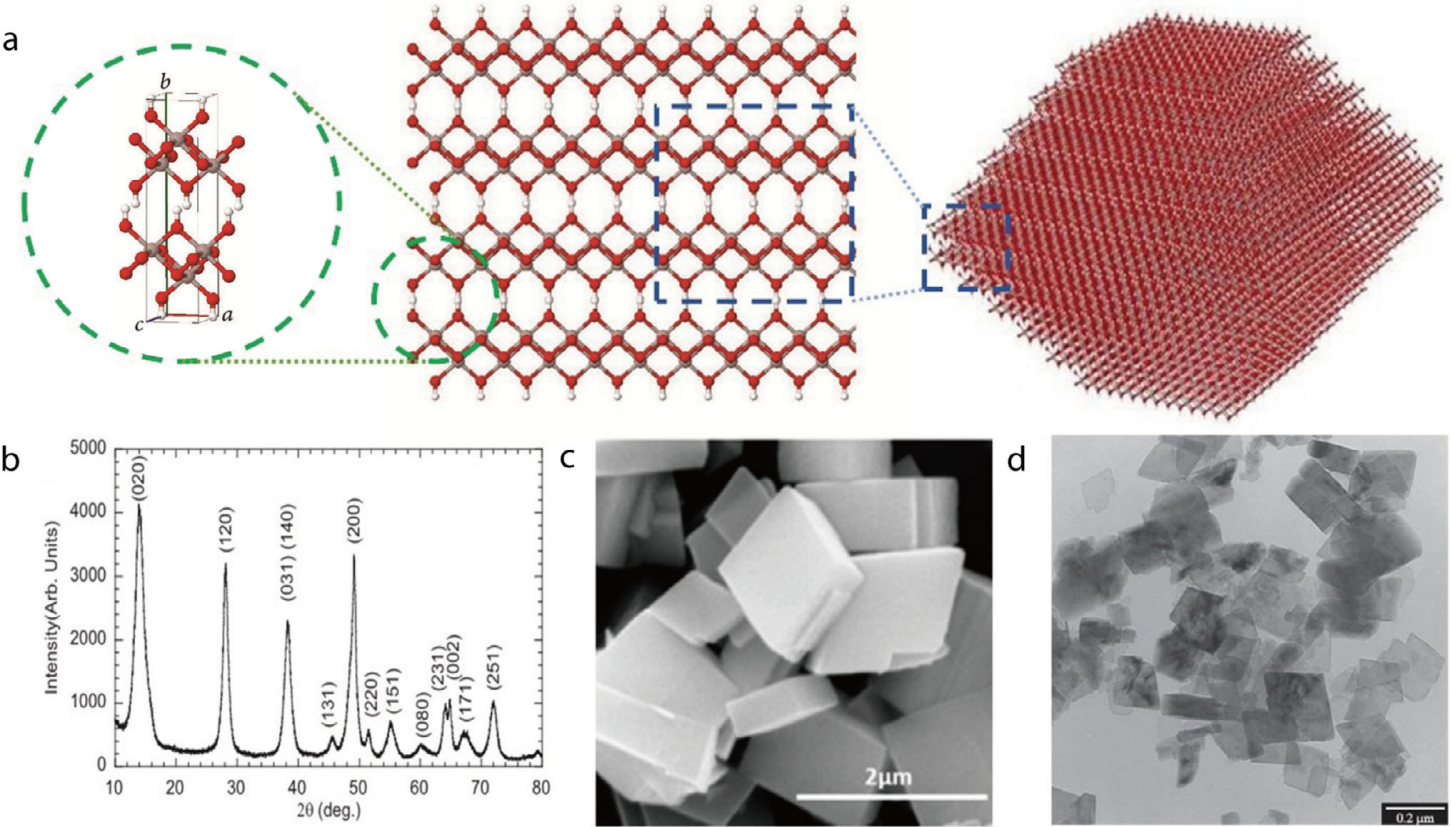
a) 3D structure of boehmite. Reproduced with permission.^[^ ^146^ ^]^ Copyright 2016, Wiley‐VCH, Copyright 2016, ECCOMAS Proceedia. b) XRD pattern of boehmite. Reproduced with permission.^[^ ^148^ ^]^ Copyright 2009, Elsevier. c) SEM image showing the boehmite morphology. Reproduced with permission.^[^ ^149^ ^]^ Copyright 2018, Elsevier. d) TEM image of boehmite particles. Reproduced with permission.^[^ ^150^ ^]^ Copyright 2009, SciELO.

Boehmite (AlOOH) comprises binary layers in which oxygen is present in a cubic packing structure. The individual layers of boehmite are connected by hydrogen bonds between the hydroxyl groups, resulting in a 3D crystal structure in the orthorhombic system (**Figure** **35**a).^[^ ^146^, ^147^ ^]^ The stable orthorhombic structure features a high density of hydroxyl groups on the surface, enabling surface modification with various functional groups. Boehmite has unique properties such as nontoxicity; high active‐phase dispersibility; biocompatibility; thermal, mechanical, and chemical stabilities; low cost; and corrosion resistance. Consequently, boehmite is used as an adsorbent, membrane, catalyst, coating, cosmetic, corrosion inhibitor, vaccine adjuvant, and composite reinforcing material for ceramics. In its XRD pattern, boehmite exhibits strong peaks originating from the (020), (200), and (120) crystallographic planes, in reference to the 21‐1307 JCPDS file (Figure 35b).^[^ ^148^ ^]^ Moreover, SEM imaging has shown that bulk boehmite contains rhombic particles with an average size of <1 µm, with a basal (010) surface and four (101) faces on the sides (Figure 35c).^[^ ^149^ ^]^ TEM imaging has revealed the morphology of the {010} cleavage plane inherited from boehmite (Figure 35d).^[^ ^150^ ^]^

**Figure 36:**
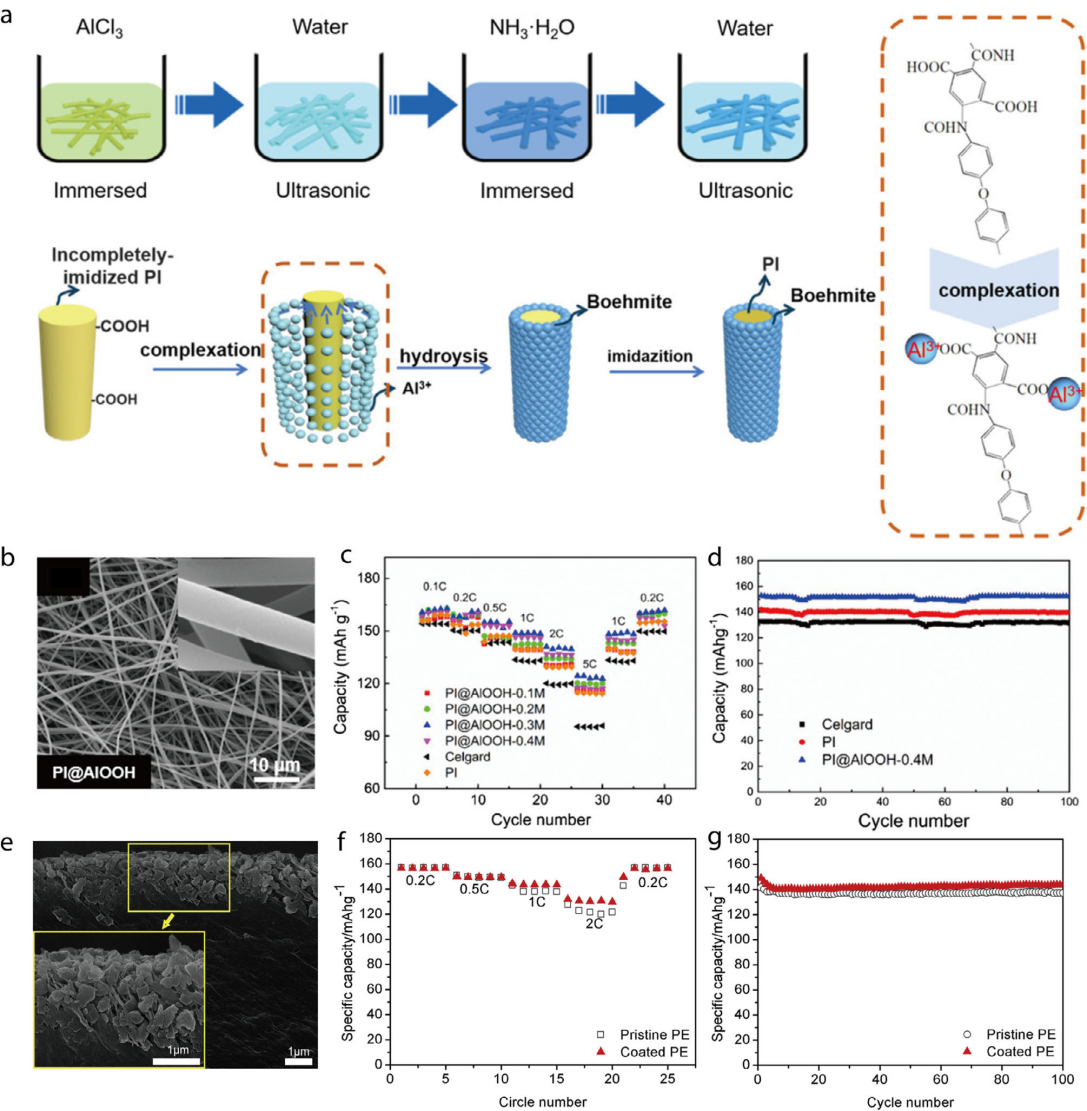
a) Illustrative protocol of the preparation of PI@AlOOH composite nanofiber membrane through the absorption/complexation−hydrolysis process. b) SEM image of PI@Boehmite composite nanofiber membrane prepared via absorption/complexation–hydrolysis using a 0.4 m aluminum chloride/ethanol solution as the precursor. c) Rate capability and d) cycling performance at 25 °C for LiFePO_4_/separator/Li half‐cells with a Celgard membrane, PI nanofiber membrane, or PI@AlOOH composite nanofiber membrane. Reproduced with permission.^[^ ^134^ ^]^ Copyright 2022, Wiley‐VCH. e) Cross‐sectional SEM image of boehmite‐coated PE membrane heated at 150 °C for 0.5 h (with inset showing an enlarged image of the coating interface). f) Discharge rate capability evaluated at a constant charge current rate of 1C over a voltage range of 1.0–2.5 V. g) Cycling performance of two CR2016 coin‐type half‐cells. Reproduced with permission.^[^ ^135^ ^]^ Copyright 2017, Elsevier.

Similar to numerous inorganic nanoparticles such as SiO_2_, TiO_2_, and Al_2_O_3_, boehmite exhibits excellent mechanical and thermal stabilities and can be incorporated into separators to enhance the stability and safety of LIBs. For instance, Kefan et al. prepared boehmite‐encapsulated PI membranes for LIBs (**Figure** **36**a).^[^ ^134^ ^]^ Essentially, boehmite was uniformly coated onto PI nanofibers via hydrolysis, in which the imidized PI nanofiber membrane was immersed in a dilute solution of ammonia and anhydrous aluminum chloride in ethanol. The addition of boehmite improved the mechanical stability of the host PI separator, as evidenced by the fact that the tensile strength of the PI@Boehmite composite nanofiber membrane (35.21 MPa) was more than six times that of the PI nanofiber membrane. Furthermore, the PI@Boehmite separator did not shrink at a high temperature of 250 °C, in contrast to the commercial Celgard membrane that completely melted. Furthermore, the electrolyte uptake and porosity of the composite were significantly higher than those of Celgard PP membranes. SEM imaging indicated that the PI@Boehmite composite nanofiber membrane had a smooth surface, validating the coaxial inorganic encapsulation strategy (Figure 36b). To assess the effect of the boehmite coating on the LIB performance, the cycling performance of LiFePO_4_/separator/Li half cells with a Celgard membrane, PI membrane, or PI@Boehmite composite nanofiber membrane was evaluated at 25 °C. At 5C, the battery with PI@Boehmite showed a considerably higher capacity (124.3 mAh g^−1^) than that of the cell with the PP membrane (95.1 mAh g^−1^) (Figure 36c). Additionally, the battery with PI@Boehmite exhibited better long‐term cycling performance and higher capacity than those of the cells with PI or Celgard membranes (Figure 36d).

Yang et al. prepared a slurry of 35 wt% boehmite particles, 1 wt% PVA, and distilled water and then coated it onto both sides of a PE membrane using roller coating equipment.^[^ ^135^ ^]^ As the temperature was increased from 110 to 150 °C, the pristine PE membrane gradually contracted and lost more than 85% of its original area. In contrast, the modified PE membrane showed significantly superior thermal stability, as it retained its shape at 140 °C. Furthermore, cross‐sectional SEM imaging of the modified PE membrane preheated at 150 °C for 0.5 h revealed that PE was partially embedded in the boehmite particle assembly, forming an interlocking interfacial structure (Figure 36e). This feature significantly increased the contact area between the boehmite particles and PE, thereby enhancing the thermal stability of the modified PE membrane. Furthermore, the boehmite particles improved the electrolyte wettability of the separator, compensating for the reduced ion mobility caused by decreased permeability. To estimate the performance enhancement after boehmite coating, the cycling performance was evaluated for two CR2016 coin‐type half‐cells (Li titanate/Li metal) with pristine or modified PE membranes as the separator. The cell assembled with the modified PE membrane exhibited a better rate performance than that of the system with pristine PE at a high current rate of 2C (Figure 36f). Additionally, the initial capacity of the cell assembled with the modified PE separator (150 mAh g^−1^) was higher than that of the system with pristine PE (142 mAh g^−1^) (Figure 36g). After 100 cycles, the cell assembled with the modified PE separator retained 96.3% of its initial capacity, surpassing that of the cell constructed using pristine PE (94.6%).

#### 2.3.2 Diatomite

**Figure 37:**
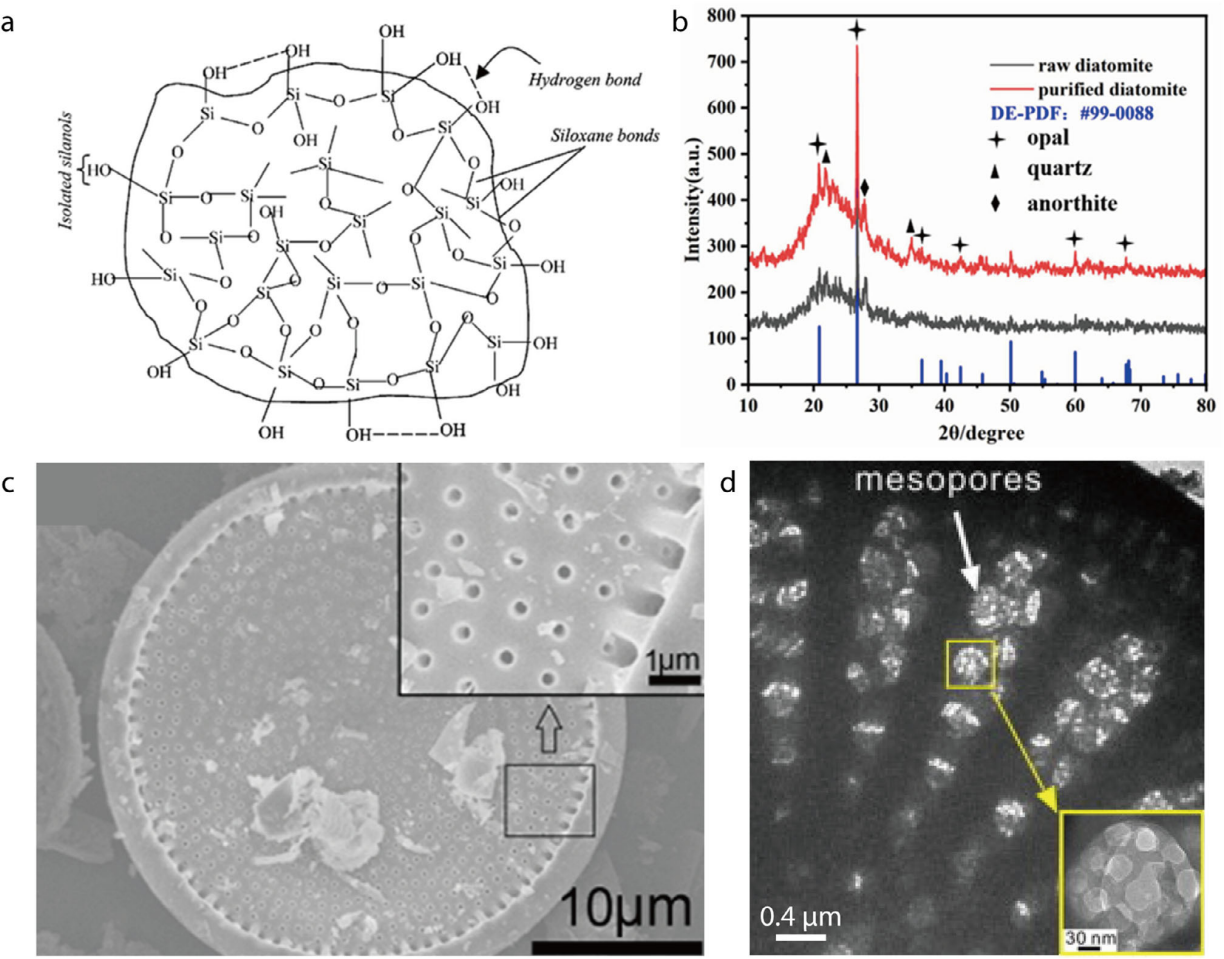
a) Various types of bonds on the surface of SiO_2_. Reproduced with permission.^[^ ^152^ ^]^ Copyright 2011, Elsevier. b) XRD patterns of raw and purified DT. Reproduced with permission.^[^ ^153^ ^]^ Copyright 2024, Multidisciplinary Digital Publishing Institute. c) SEM image of DT. Reproduced with permission.^[^ ^154^ ^]^ Copyright 2021, Elsevier. d) TEM image of DT, showing the detailed porous structure. Reproduced with permission.^[^ ^155^ ^]^ Copyright 2013, Elsevier.

Diatomite (DT) is a natural biological mineral obtained via the accumulation and deposition of unicellular aquatic plankton and mainly comprises SiO_2_•nH_2_O.^[^ ^151^ ^]^ Isolated silanol groups (–SiOH), free dual silanol groups (–Si(OH)_2_), and –Si–O–Si bridges with oxygen atoms are distributed on the surface of SiO_2_ (**Figure** **37**a).^[^ ^152^ ^]^ The silanol groups on the surface of DT cause it to have a high affinity for electrolytes.^[^ ^136^ ^]^ XRD analysis has indicated that the main components of raw and purified DT include amorphous Al_2_O_3_, Fe_2_O_3_, and SiO_2_, as well as a small number of other organics and oxides (Figure 37b).^[^ ^153^ ^]^ DT often contains impurities such as opal, dolomite, and quartz. At the microscopic scale, DT exhibits various shapes depending on the microalgae from which it originates. For example, one DT specimen has round sieve‐shaped particles with uniformly distributed ≈0.3‐µm‐sized pores on the surface (Figure 37c).^[^ ^154^ ^]^ This pore shape has also been observed by TEM, which has revealed a smooth, clean surface with open pores (Figure 37d).^[^ ^155^ ^]^ Because of its high porosity, large specific surface area, high water absorption capacity, low bulk density, high purity, affordability, and light weight, DT has been widely utilized as an insulation material, absorbent, filter, adsorbent, and coating material.

DT exhibits multiple beneficial properties as a filler material for LIB separators. It has large, well‐developed pores with high porosity and is rich in silanol groups, thereby increasing the electrolyte uptake of the separator. By leveraging these attributes, Li et al. prepared a composite membrane for LIBs by coating purified DT onto a polyethylene terephthalate (PET) nonwoven fabric.^[^ ^136^ ^]^ They prepared a slurry of purified DT in a solution of dimethylformamide (DMF) and PVDF, which was then coated onto a PET nonwoven substrate using a doctor blade with 150 µm spacing. The pore structure of the PVDF separator hindered the transport of Li ions. In contrast, the porous structure of the DT composite separators provided better channels for Li‐ion transport. As the DT content increased, the wettability of the membrane improved and its thermal shrinkage rate decreased, thus enhancing the high‐temperature stability of the LIB. To ascertain the effects of DT on the discharge capacity and coulombic efficiency, the synthesized separators were tested in cells for 100 cycles at 0.5C. The cell assembled with a composite separator with a DT content of 60 wt% (DP‐60) exhibited a decrease in discharge capacity after 70 cycles, presumably owing to the presence of large pores on the surface. However, the cells assembled with DP‐70 or DP‐80 composite separators exhibited improved discharge capacities (143.2 and 144.3 mAh g^−1^, respectively) and capacity retention rates (98.79% and 98.26%) after 100 cycles. Subsequent rate performance tests performed at current rates of 0.2C–4C indicated that separators with higher DT content showed improved discharge capacities, with the DP‐80 separator exhibiting optimal C‐rate performance and excellent cycling stability.

**Figure 38:**
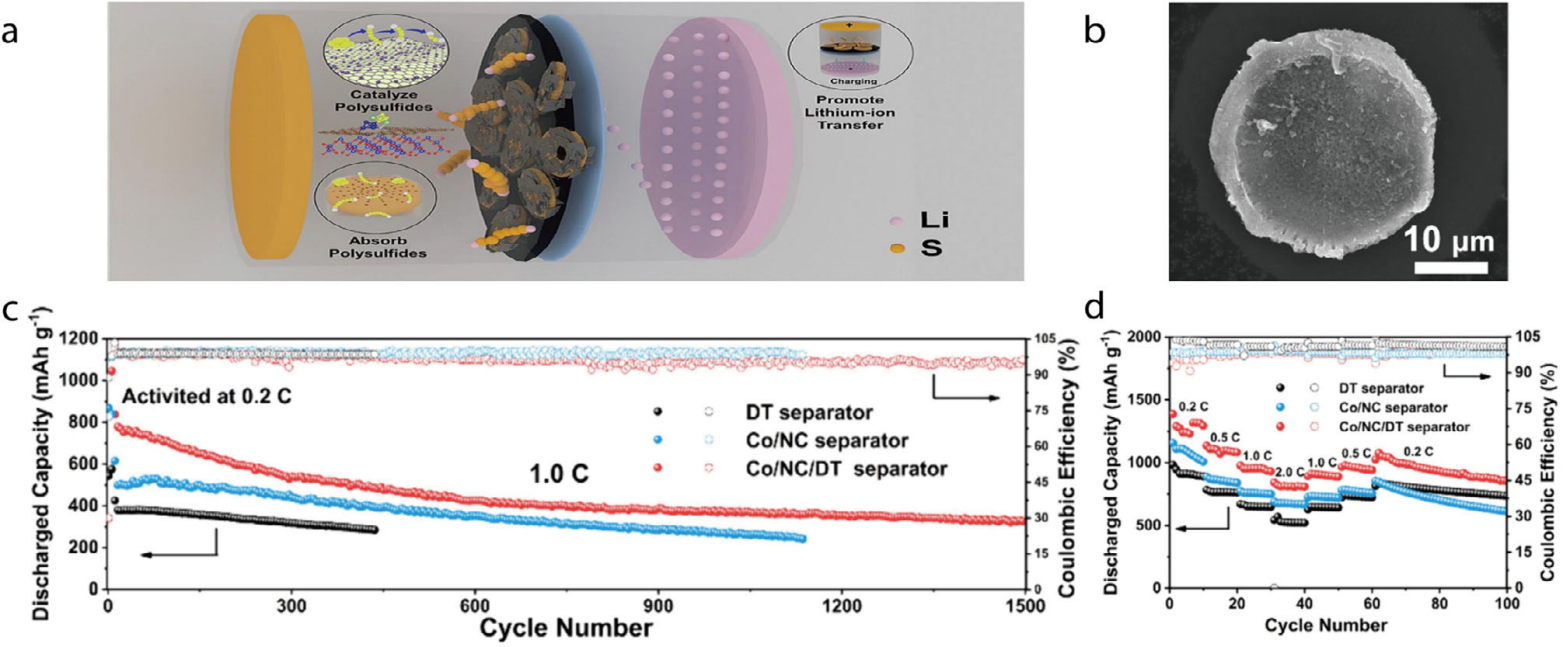
a) Schematic showing polysulfide absorption, catalysis, and Li⁺ transport promotion. b) SEM image of Co/NC/DT. c) Cycling performance at 1.0C of Co/NC/DT, Co/NC, and DT modified separators for Li‐S batteries. d) Rate performance of Co/NC/DT, Co/NC, and DT modified separators for Li‐S batteries. Reproduced with permission.^[^ ^156^ ^]^ Copyright 2024, Elsevier.

In another study, Yan et al. fabricated a multifunctional separator for Li–S batteries by using DT as a structural support to anchor nitrogen‐doped carbon with highly loaded cobalt nanoclusters (Co/NC/DT). A zeolitic imidazolate framework precursor based on Co and Zn was mixed with DT and molten salts (LiCl, KCl), then calcined at 400 °C to exfoliate the zeolitic imidazolate framework structure. A second calcination at 800 °C removed Zn and allowed nitrogen‐doped carbon and Co nanoclusters to be immobilized within the DT pores. For comparison, cobalt catalyst separators without DT (Co/NC) and pristine diatomtie were also prepared as controls. The resulting materials were mixed with Super P and PVDF to form a slurry, which was coated on the cathode side of a Celgard 2500 separator for cell assembly. Co/NC/DT can effectively adsorb soluble polysulfides through the porous DT framework and the active sites on the Co–NC surface, thereby suppressing the shuttle effect, catalyzing polysulfide conversion, and facilitating Li⁺ transport (**Figure** **38**a).^[^ ^156^ ^]^ An H‐type cell experiment confirmed the excellent polysulfide‐blocking ability of the Co/NC/DT separator. When using the PP separator, the initially clear solution gradually turned pale yellow after 6 h and became fully yellow after 24 h, whereas the solution with the Co/NC/DT separator showed almost no color change after 12 h and only a slight yellow tint after 24 h. A SEM image showed that DT retained its original disc‐like structure (≈200 µm diameter) and pore size (3–5 µm) after calcination, confirming good thermal stability (Figure 38b). A high‐resolution TEM further revealed ordered lattice fringes with a spacing of 2.06 Å corresponding to the Co (111) plane, verifying the presence of Co nanoclusters with sizes of several tens of nanometers. Depth‐profile XPS analysis and density‐functional theory calculations indicated that the addition of DT converted the dominant nitrogen species in the carbon matrix from pyridinic N to pyrrolic N, enhancing the binding stability of Co nanoclusters and promoting stronger polysulfide adsorption and conversion activity. Tafel plots and Li⁺ transference number measurements also confirmed that the Co/NC/DT separator provides high Li⁺ conductivity, which supports uniform Li deposition on the anode. Electrochemical performance tests showed that the Li–S battery with the Co/NC/DT separator delivered an initial discharge capacity of 1548.7 mAh g^−1^ at 0.1C and maintained 998.6 mAh g^−1^ after 50 cycles, outperforming Co/NC (848.5 mAh g^−1^) and DT (827.1 mAh g^−1^). At 1.0C, all batteries exhibited stable coulombic efficiency, but the Co/NC/DT battery maintained excellent long‐term stability with over 1500 cycles and an ultra‐low capacity decay rate of only 0.039% per cycle (Figure 38c). Furthermore, the Co/NC/DT battery achieved the highest discharge capacity of 1387.5 mAh g^−1^ at 0.2C in rate performance tests, while the DT‐only battery showed good stability at low rates but significant performance drop at higher rates (Figure 38d).

#### 2.3.3 Zeolite

**Figure 39:**
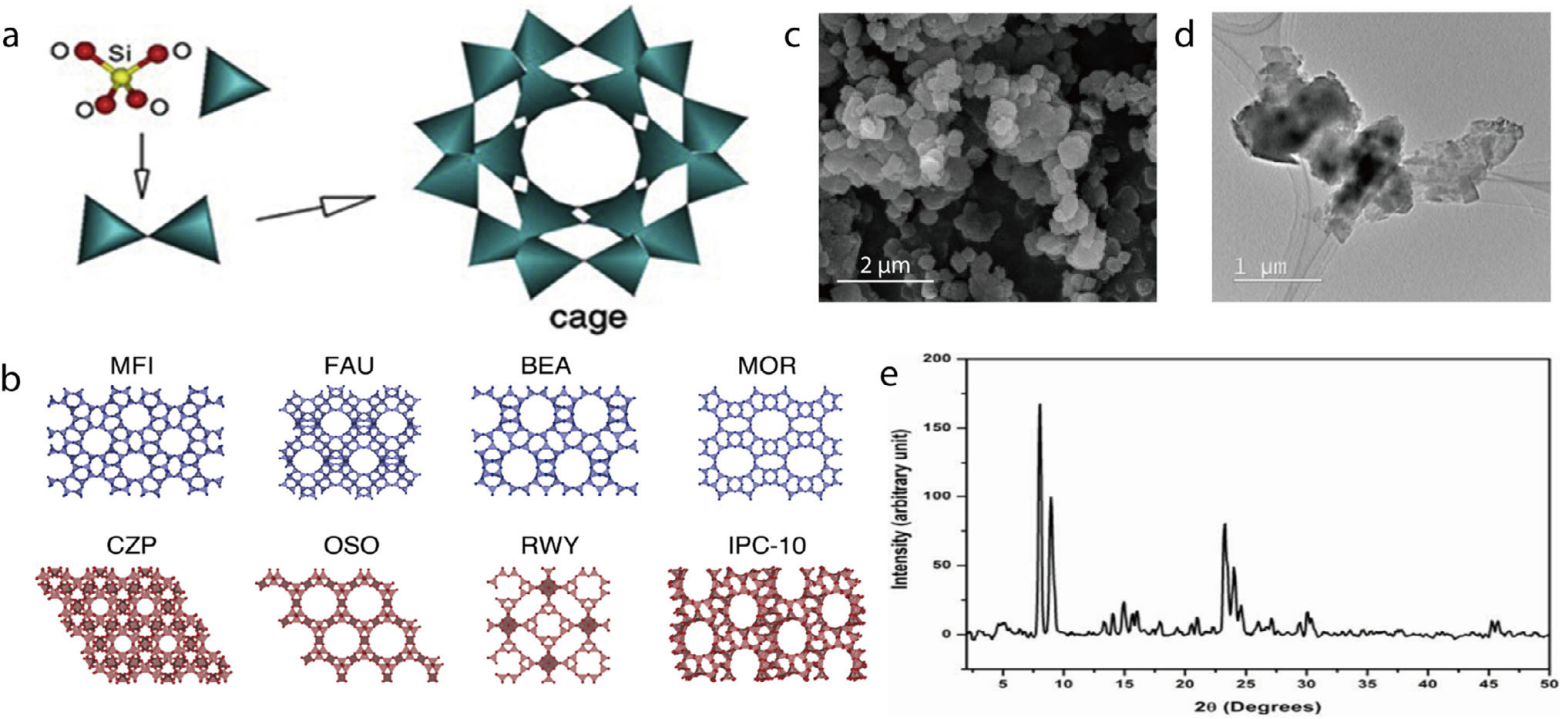
a) Structure of zeolite. Reproduced with permission.^[^ ^158^ ^]^ Copyright 2019, Elsevier. b) Representative zeolite framework structures (ZSM‐5 (MFI), faujasite (FAU), beta (BEA), mordenite (MOR), clinoptilolite (CZP), offretite (OSO), rubidium‐filled zeolite Y (RWY), and Institute of Physical Chemistry‐10 (IPC‐10)). Reproduced with permission.^[^ ^159^ ^]^ Copyright 2016, American Chemical Society. c) SEM image of zeolite. Reproduced with permission.^[^ ^160^ ^]^ Copyright 2016, Elsevier. d) TEM image of zeolite. Reproduced with permission.^[^ ^161^ ^]^ Copyright 2023, Elsevier. e) XRD pattern of MFI zeolite. Reproduced with permission.^[^ ^162^ ^]^ Copyright 2017, International Water Association Publishing.

Zeolites are porous hydrated 3D‐structured aluminosilicates. The primary building units (PBUs)—SiO_4_ and aluminum (AlO_4_)—are connected by common oxygen atoms to form secondary building units (SBUs). These structures can be filled with ions and water molecules and provide significant freedom of movement. According to Löwenstein's rule, silicon–oxygen tetrahedra can be adjacent to each other (Si–O–Si), whereas aluminum–oxygen tetrahedra can only be connected to the silicon–oxygen tetrahedra (Si–O–Al). Thus, replacing the Si^4+^ cation in the tetrahedral position with Al^3+^ makes the aluminosilicate framework negatively charged, thus attracting positive cations such as K^+^, Na^+^, Ca^2+^, and Mg^2+^.^[^ ^137^, ^157^ ^]^ Zeolites exhibit good mechanical and thermal properties, high surface areas, and tunable surface characteristics such as basicity/acidity and hydrophobicity/hydrophilicity.^[^ ^140^, ^157^ ^]^ Natural zeolites primarily originating from volcanic sources include clinoptilolite, mordenite, and chabazite. Over 100 types of zeolite structures have been synthetically produced using methods such as alkali activation and hydrothermal synthesis (**Figure** **39**a).^[^ ^158^ ^]^ Because certain zeolites have the same general structure but differ only in composition, the concept of the framework type has been introduced to classify them. The framework type is expressed as a three‐letter code, with each framework corresponding to a unique set of building blocks, regardless of its composition. For instance, Zeolite Socony Mobil‐5 (ZSM‐5) and silicalite are categorized as the same framework type—that is, MFI (derived from ZSM‐5)—although they have different Si and Al compositions. To date, various framework types have been defined using three‐letter codes (Figure 39b).^[^ ^159^ ^]^ Zeolites have often been observed as mesoporous structures with an amorphous framework by both SEM and TEM (Figure 39c,d).^[^ ^160^, ^161^ ^]^ In its XRD pattern, MFI zeolite exhibits high crystallinity with characteristic peaks at 2*θ* values of 8° and 23.5° (Figure 39e).^[^ ^162^ ^]^ Compared with natural zeolites, synthetic zeolites have more uniform pore sizes and allow control over the Al content during synthesis, significantly enhancing their adsorption capacity. Consequently, synthetic zeolites are being used more frequently.^[^ ^140^, ^163^ ^]^

The high porosity and mechanical stability of zeolites have been exploited to prepare effective zeolite‐coated separators for batteries, particularly LIBs. For example, Li et al. fabricated a ZSM‐5/PI composite separator for a LiFePO_4_/Li cell by phase inversion using a PI matrix and the ZSM‐5 zeolite filler.^[^ ^137^ ^]^ ZSM‐5 contributed to the electrolyte uptake and wettability, whereas PI affected the mechanical strength and thermal stability. The addition of 5% or 10% ZSM‐5 (resulting samples denoted Z/PI‐5 or Z/PI‐10, respectively) led to smaller pores and better interpore connectivity, which improved the ion transport and hindered the growth of Li dendrites. However, when 15% ZSM‐5 was added (Z/PI‐15), the pore size increased without a change in the number of pores, leading to a decrease in tensile strength. Therefore, the Z/PI‐10 composite separator was considered an effective specimen considering both the porosity and mechanical strength. Z/PI‐10 showed excellent thermal stability, exhibiting no shrinkage even after treatment at 180 °C. This was attributed to the stable aromatic heterocyclic structure of the PI molecular chain. Moreover, Z/PI‐10 exhibited excellent wettability, influenced by the highly interconnected sponge‐like pore structure and excellent electrolyte affinity of ZSM‐5. The abundant interconnected micropores of the ZSM‐5 nanoparticles and the Lewis acid–base interactions between ZSM‐5 and the electrolyte promoted the free transport of Li ions, thereby improving the charge–discharge properties of the battery. A LiFePO_4_/Li cell assembled with the Z/PI‐10 composite separator showed better cycle performance and rate capability than that of the cells with the PP or pristine PI separators.

Dong et al. applied a thin coating of MFI zeolite onto both sides of a commercial PP separator (ZCPP) to enhance the performance of Li_4_Ti_5_O_12_/Li cells.^[^ ^44^ ^]^ This separator featured a significantly thinner zeolite layer (2 µm thick on each side) than the PP layer (20 µm). When the liquid electrolyte (16 µL) was added dropwise onto the surface of the separator, the ZCPP25 separator (Si/Al ratio = 25:1) exhibited greater wettability than that of the PP separator. Additionally, the channel diameter of the MFI‐type zeolite was larger than the molecular sizes of DMC, DEC, and LiPF_6_. The Li_4_Ti_5_O_12_/Li cell assembled with the ZCPP25 separator exhibited higher cycling stability and charge–discharge capacity than those of the cell with an uncoated PP separator.

Various types of zeolites with different framework structures have been utilized in the same context. Xu et al. coated Li‐ion‐exchanged zeolite (Li‐zeolite) onto both sides of Celgard 2320.^[^ ^138^ ^]^ The resulting separator absorbed adjacent trace water to reduce the formation of HF and acted as an HF scavenger to suppress damage to the positive electrode. Moreover, it inhibited capacity fading due to transition metal reduction on the graphite surface by capturing transition metal ions dissolved in the electrolyte. Experimental results confirmed that battery performance metrics such as discharge rate, coulombic efficiency, and cycling performance were improved in a full‐cell LIB with a graphite anode and a composite cathode fabricated using LiNi_0.5_Mn_0.3_Co_0.2_O_2_ (NMC532) and LMO.

Xiao et al. coated NaA‐type zeolite/PVDF‐*co*‐hexafluoropropylene (PVDF–HFP) onto a PET nonwoven to fabricate an NZP composite separator.^[^ ^139^ ^]^ The separator exhibited improved wettability, electrolyte uptake, thermal dimensional stability, and ionic conductivity. In a battery with LiFePO_4_ as the cathode and Li metal as the anode, the NZP composite separator exhibited a higher C‐rate capability and cycling performance than those of the Celgard 2400 separator. These improvements were attributed to the development of a microporous structure and strong affinity for large ions (such as PF_6_^−^) and polar solvents, which favorably affected ionic transport and electrolyte retention during cycling.

Sabetzadeh et al. coated both sides of a PP separator with the 4A zeolite using a PVDF binder and then plasma‐treated it to create a composite separator.^[^ ^140^ ^]^ They compared several separators, including a 4A‐zeolite‐coated separator (4A‐PP), plasma‐treated uncoated separator (P‐PP), plasma‐treated 4A‐zeolite‐coated separator (P‐4A‐PP), and PP separator. Among these separators, P‐4A‐PP exhibited the highest porosity and air permeability. The thermal stability of the separators was evaluated by heat‐treating them at 175 °C for 45 min. The results showed that PP and P‐PP melted completely, whereas P‐4A‐PP exhibited slightly more thermal shrinkage than that of 4A‐PP. This was because the degradation affinity of the PP and PVDF binders improved after plasma treatment. The 4A zeolite induced strong interactions between the particles and polar liquid electrolytes, and its porous structure enhanced the electrolyte uptake. Plasma treatment created oxygenated chemical groups on the surface without affecting the zeolite–PVDF adhesion, thus enhancing the electrolyte adsorption and electrochemical performance. When the separator was integrated into a Li(Ni_0.8_Co_0.1_Mn_0.1_)O_2_/Li‐metal cell, the rate capability and performance stability were improved.

Shekarian et al. coated the 4A zeolite onto the upper face of commercial PP separators using PVDF as a binder.^[^ ^141^ ^]^ By coating these heat‐resistant materials, all the coated membranes prepared at different constituent ratios exhibited low thermal shrinkage, with the degree of shrinkage decreasing with increasing zeolite content. The surface properties of the 4A zeolite particles and Lewis acid–base interactions enhanced the electrolyte absorption and wetting properties. However, as the zeolite content increased, a denser‐coated layer was formed, resulting in increased thickness and decreased wetting properties. Conversely, when the zeolite content decreased, the interparticle gaps within the 4A zeolite were filled with PVDF, reducing the porosity of the coated layer. As a representative separator, a specimen with a zeolite‐to‐binder ratio of 8 (PPA8) was fabricated and subjected to electrochemical performance analysis in a LiFePO_4_/Li half‐cell. The cell assembled with the PPA8 separator exhibited a higher initial discharge capacity than that of the cell assembled with the PP separator. The discharge capacity retention of the cells assembled with the PPA8 or PP separators was 96.2% and 83.4%, respectively, indicating an improvement in cyclability. Furthermore, rate analysis revealed good C‐rate performance, especially at high current densities, which was attributed to the greater influence of ion transfer under ohmic polarization.

**Figure 40:**
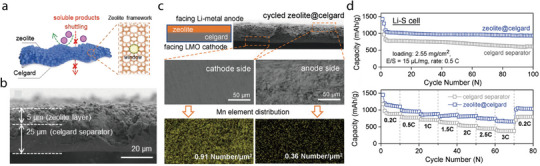
a) Schematic illustration of zeolite@celgard and working mechanism of zeolite molecular sieve. b) Cross‐sectional SEM image of zeolite@Celgard. c) Cross‐sectional SEM images of cycled zeolite@Celgard separator and EDS mapping images of Mn collected on cathode side (left) and anode side (right) of cycled zeolite@Celgard. d) Cycling and rate performance data of Li–S battery. Reproduced with permission.^[^ ^142^ ^]^ Copyright 2023, Wiley‐VCH.

Similar to other minerals with Lewis active sites, zeolite has also been tested as a coating to prevent polysulfide shuttling in Li–S batteries. For example, Li et al. fabricated zeolite@Celgard separators by coating a thin zeolite layer (5 µm) on a Celgard separator and applying an external pressure of 8 MPa (**Figure** **40**a,b).^[^ ^142^ ^]^ The external pressure reduced the interparticle gaps between the zeolite particles, whereas the thin coating bestowed flexibility and grid‐scale fabrication potential to the separator. The main objective was to suppress the shuttling of soluble species (Mn^2+^ (5.6 Å), polysulfides (5.7 Å), and Tetrathiafulvalene (TTF^+^, 7.9 Å)) through the ordered pore‐window (4 Å) of the zeolite molecular sieve by leveraging the hypothesized pore‐size effect of zeolites. The shuttling of soluble species induces side reactions such as anode corrosion, recharge with low coulombic efficiency, and active material outflow with capacity/energy fading. The zeolite@Celgard separator resolved the shuttling/crossover effect by suppressing soluble species, such as H_2_O/HF scavengers. A battery was assembled with the zeolite@Celgard side facing the Li metal and the Celgard side facing the cathode. EDX analysis was performed to confirm the restraining effect on soluble Mn^2+^ species. The amount of accumulated Mn in the cycled zeolite@Celgard was relatively higher on the cathode side (0.91 N µm^−2^) than on the anode side (0.36 N µm^−2^), indicating that zeolite@Celgard could effectively block soluble Mn species (Figure 40c). Furthermore, Li–S batteries assembled with Celgard or zeolite@Celgard were cycled at 0.5C; after 100 cycles, the capacity retention was 77.3% and 89.5%, respectively, indicating an improvement in cycling performance (Figure 40d). The battery assembled with zeolite@Celgard exhibited superior rate capability, with specific capacities of 1106.4, 953.6, 879.5, 849.1, 817.2, 742.9, and 659.0 mAh g^−1^ observed at charge rates of 0.2C, 0.5C, 1.0C, 1.5C, 2.0C, 2.5C, and 3.0C, respectively, which were higher than those of the battery assembled with the Celgard separator. Additionally, in Li/LiMn_2_O_4_ and Li/O_2_ battery systems, the zeolite‐based separator exhibited better electrochemical cyclability, reversibility, cycle, and rate performance than those of the conventionally used Celgard/GF separators. Moreover, the authors highlighted the potential of using the zeolite‐based separator in other rechargeable battery systems such as alkali (Li, Na, K)–halogen/sulfur batteries and flowing batteries.

#### 2.3.4 3D Mineral Summary

**Figure 41:**
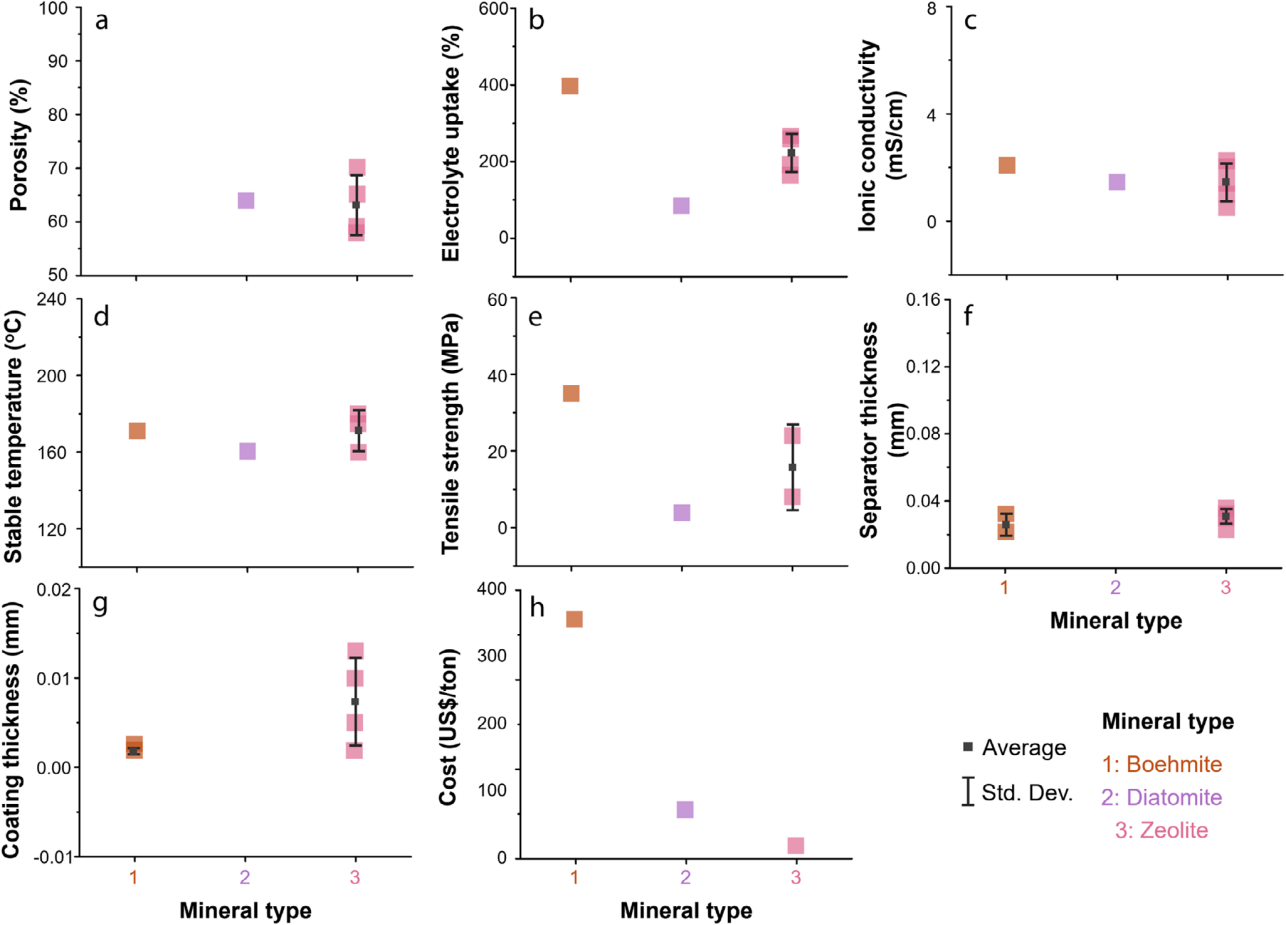
Dependence of separator parameters—a) porosity, b) electrolyte uptake, c) ionic conductivity, d) stable operating temperature, e) tensile strength, f) separator thickness, g) coating thickness, and h) cost—on the type of 3D mineral; Average and standard deviation of the data are indicated as black squares and vertical lines, respectively.

**Figure 42:**
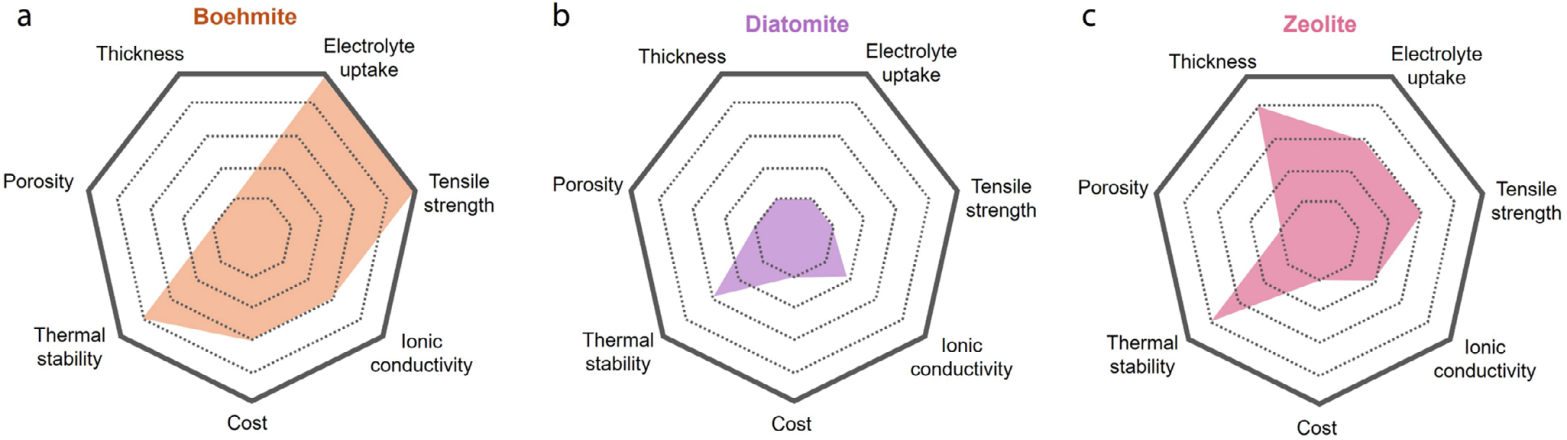
Radar plots for parameters of separators fabricated using different types of 3D minerals.

To evaluate the relative performance of 3D minerals, we compared the reported performance indicators of 3D minerals (**Figures** **41** and **42**). 3D minerals commonly exhibit superior thermal stability owing to their strong Si–O and Al–O bonds, which resist deformation even at elevated temperatures, which is particularly true for boehmite and zeolite with excellent tensile strength. These two minerals also exhibit high electrolyte uptake and reasonable ionic conductivity, attributed to their abundant surface hydroxyl groups. In contrast, DT‐based separators, as reported to date, generally show lower mechanical strength and reduced electrolyte uptake. Since the physical properties of DT vary depending on the microorganism or aquatic plant from which it is synthesized, these findings suggest the need for further research on DT sources with superior mechanical properties. From a cost perspective, boehmite is the most expensive among the three, due to the need for high‐purity synthesis and additional purification steps. In summary, boehmite is advantageous when high performance is prioritized regardless of cost, while zeolite offers a cost‐effective alternative with moderate performance, making it more suitable for budget‐conscious applications.

## 3 Conclusion

### 3.1 Performance summary

In summary, mineral coating is an effective method for improving the performance of LIBs because it enhances multiple separator properties such as mechanical strength, thermal stability, and Li‐ion diffusion kinetics. Various parameters have been measured to quantitatively estimate the characteristics of separators improved by mineral coating (**Table** **4**); these include porosity, electrolyte uptake, ionic conductivity, stable operating temperature, tensile strength, and thickness. These parameters are closely related to the performance and durability of mineral‐coated separators, as described below.

**Table 4:** Comparison of separator parameters that influence the electrochemical performance of Li‐ion batteries.

Type	Mineral	Host separator	Thickness [mm]	Porosity [%]	Electrolyte uptake [%]	Tensile strength [MPa]	Stable temperature [°C]	Ionic conductivity [mS cm^−1^]	Reference
1D	Attapulgite	Celgard2400	0.03	45.8	168	92	170	0.78	[45]
		SA	0.02	68.0	420	6	250	1.15	[46]
	Chrysotile	N/A	0.07	83.0	282	N/A	120	3.93	[47]
	Sepiolite	PVA‐PIB	0.02	36.9	213	N/A	200	1.38	[48]
		PU	0.08	64.0	268	2.70	150	1.37	[49]
		PP	0.03	N/A	625	N/A	140	0.62	[50]
	Halloysite	PP	0.03	71.0	225	43	180	0.66	[51]
		A4 paper	0.02	76.8	295	20.1	180	0.42	[52]
		BC	0.03	83.0	369	84.4	180	5.13	[53]
		PVA	0.20	78.0	N/A	12.5	180	1.14	[54]
		PE	0.20	N/A	208	N/A	150	0.42	[55]
		PVA	0.10	90.0	700	18	180	4.92	[56]
		Celgard3501	0.05	N/A	N/A	N/A	N/A	N/A	[57]
		SPEEK	0.08	N/A	48.4	26.2	300	N/A	[43]
2D	Albite	N/A	0.30	57.0	151	19	N/A	0.72	[80]
	Dickite	ES	0.31	61.6	86.1	9	160	3.16	[81]
	Hectorite	PBI	0.03	79.0	413	26.4	200	2.39	[82]
	Illite	Celgard2400	0.04	N/A	N/A	N/A	N/A	0.83	[83]
	Kaolinite	GF	N/A	N/A	N/A	N/A	N/A	13.7	[84]
	Laponite	CB‐Celgard	0.03	N/A	N/A	N/A	160	0.59	[85]
		CNF	0.03	68.0	260	143.3	200	0.98	[86]
		PVDF	0.05	74.0	420	N/A	150	0.72	[87]
	Montmorillonite	PVDF	0.06	84.1	333	2.39	161	4.20	[88]
		PE	0.04	N/A	349	N/A	160	3.63	[89]
		PES	0.05	N/A	85.0	36.0	N/A	11.0	[90]
		Celgard2325	0.03	N/A	247	N/A	300	4.20	[90]
		PI	0.02	87.3	110	26.23	180	2.39	[92]
		PE	0.03	N/A	349	3.39	80	4.31	[93]
		PP	0.04	N/A	N/A	N/A	350	0.64	[94]
		PVDF	0.15	87.0	802	N/A	165	5.61	[95]
	Vermiculite	Celgard2500	0.03	N/A	157	N/A	168	N/A	[96]
		Celgard3401	0.05	N/A	N/A	N/A	N/A	N/A	[97]
		N/A	0.09	84.9	645	7.80	200	1.02	[98]
		PVDF	0.06	81.7	447	14.9	165	1.68	[99]
3D	Boehmite	PI	0.03	80.0	392	35	250	2.04	[134]
		PE	0.02	N/A	N/A	105	170	6.56	[135]
	Diatomite	PET	0.12	63.8	86.6	4	160	1.43	[136]
	Zeolite	PI	0.03	59.0	260	24	180	1.04	[137]
		Celgard2320	0.03	N/A	N/A	N/A	N/A	N/A	[44]
		Celgard2500	0.02	N/A	168	N/A	N/A	0.52	[138]
		PET	0.03	65.0	194	N/A	N/A	2.10	[139]
		Celgard	0.03	70.0	522	N/A	175	1.45	[140]
		Celgard2400	0.04	58.0	270	N.A	160	2.25	[141]
		Celgard	0.03	N/A	N/A	8	N/A	N/A	[142]

Porosity is a measure of the empty space in a separator and indicates its ability to store the electrolyte. Separators with high porosity can maintain an abundant amount of electrolyte and enable fast ionic transport.^[^ ^164^ ^]^ The amount of electrolyte stored in the separator is often referred to as the electrolyte uptake, which can be calculated as $\;\frac{W_{wet}-W_{dry}}{W_{dry}}\times 100\;\left(\%\right)$, where *W*_dry_ and *W*_wet_ are the weights of the dry and electrolyte‐soaked separator, respectively. However, high porosity is not always beneficial, as it can decrease the mechanical strength of the separator and lower the durability of the battery. The mechanical strength of a separator is commonly expressed using the (ultimate) tensile strength and can be measured using tensile tests. A high tensile strength is desirable for separators because it indicates the maximum stress that a separator can withstand under external forces. Another parameter that is closely linked to the tensile strength is the thickness, with a thicker separator tending to boost the mechanical properties and durability of batteries.^[^ ^21^ ^]^ However, because thick separators have a high ionic resistance, which decreases the energy and power density of batteries,^[^ ^165^ ^]^ the optimal thickness that satisfies both the durability and power density requirements must be determined. Considering the durability of the separators, the stable operating temperature is another important parameter. This aspect represents the maximum temperature at which a separator can be used without thermal shrinkage, and is directly related to battery safety. Finally, to quantify the degree of Li‐ion diffusion in the separator, the ionic conductivity is determined for separators using the equation $\sigma =\frac{l}{R_{b}A}$, where *l*, *A*, and *R_b_* are the separator thickness, separator area, and bulk resistance, respectively.^[^ ^166^ ^]^

### 3.2 Future Perspectives of Mineral‐Modified Separators

**Figure 43:**
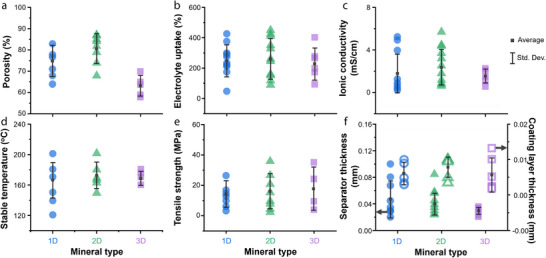
Dependence of separator parameters—a) porosity, b) electrolyte uptake, c) ionic conductivity, d) stable operating temperature, e) tensile strength, and f) separator thickness—on the type of mineral; Data from 1D‐, 2D‐, and 3D‐mineral‐based separators are denoted using blue circles, green triangles, and purple squares, respectively. Average and standard deviation of the data are indicated as black squares and vertical lines, respectively.

To understand the manner in which the type of mineral affects the performance of the separator, we correlated the aforementioned separator parameters with the type of mineral (**Figure** **43**). The porosity of the 1D and 2D mineral‐coated separators (Figure 43a) are higher than separators coated with 3D minerals. This is because 3D minerals tend to have fewer empty spaces between the mineral microparticles when coated on the separator. Among the various separator parameters, electrolyte uptake is closely related to porosity. Because minerals with larger pores can store more electrolyte, the electrolyte uptake of a separator is generally proportional to its porosity. However, close examination reveals that, although separators coated with 3D minerals have relatively low porosity, their electrolyte uptake does not decrease proportionally (Figure 43b), suggesting that other factors beyond porosity may influence electrolyte absorption. One of the key factors is the pore structure within separator. This is because even separators with the same porosity can exhibit different surface areas depending on the size and distribution of the pores, resulting in differing electrolyte uptake. In a relative study, fibrous separators, made from the same PVDF resin, are reported to exhibit superior electrolyte uptake when being fabricated via shear spinning compared to the other techniques of meltblown or electrospinning, due to their more uniform and widely distributed pores.^[^ ^167^ ^]^ Likewise, in a previous study that compared three types of separators—foam, non‐woven fabric, and uniaxially stretched—the foam‐type separator with the most uniform pore structure demonstrated the highest electrolyte uptake.^[^ ^168^ ^]^ This study attributed the enhanced electrolyte uptake to the uniform distribution of electrolyte throughout the separator, which minimizes the Li⁺ concentration gradient and thereby improves absorption of liquid electrolyte.

Another important factor that determines the electrolyte uptake is the (hydrated) cations within minerals. When comparing the electrolyte uptake of separators (Figure 43b), separators with 2D minerals retain a higher amount of electrolyte than separators with 1D minerals. This phenomenon presumably emanates from the (hydrated) cations located between the 2:1 layered structures of the 2D minerals, the ions of which can be easily replaced with the surrounding solvent/solute molecules to store extra electrolyte. The (hydrated) cations in the 2D minerals not only improve electrolyte uptake but also enhance ionic conductivity. These improvements stem from the rapid ionic exchange between cations and Li ions in the electrolyte, which accelerates the Li‐ion diffusion rate. The enhanced Li diffusion kinetics allows 2D‐mineral‐coated separators to exhibit higher ionic conductivity than that of 1D‐ or 3D‐mineral‐based separators (Figure 43c). Between 1D and 3D minerals, 1D minerals are generally more effective in improving ionic conductivity because 1D kaolin minerals with tubular particles (such as halloysite and chrysotile) can facilitate Li‐ion transport via oppositely charged surfaces.^[^ ^59^ ^]^

Unlike the separator properties discussed above, the stable operating temperature is more or less similar regardless of the type of mineral, with the average temperature for 1D‐, 2D‐, and 3D‐mineral‐coated separators being 166.4, 172.42, and 169.0 °C, respectively (Figure 43d). This is attributed to the similar phase‐transition temperatures of the clay minerals, which are mostly thermally stable below ≈300 °C, as indicated by differential thermogravimetry.^[^ ^43^, ^90^, ^91^ ^]^ The majority of mineral‐coated separators are reported to be stable over 150 °C, which is sufficiently high for shrinking conventional Celgard separators, indicating that mineral coating effectively improves the thermal stability of batteries. Mineral coatings, particularly those of 3D minerals, are also beneficial for enhancing the tensile strength of separators (Figure 43e). Among the various 3D minerals, boehmite appears to be the optimal coating material for increasing the tensile strength. A close examination of high‐tensile‐strength separators (such as halloysite‐ and laponite‐based separators) reveals that they also exhibit low ionic conductivity (<1.0 mS cm^−1^) and poor electrolyte uptake (<300%).^[^ ^45^, ^86^ ^]^ This phenomenon can occur when clay minerals are densely coated, with good adhesion between mineral particles improving the mechanical strength, whereas the lack of empty space to store the electrolyte results in poor ionic conductivity. Considering the average thickness of the mineral‐coated separators, those with 3D minerals exhibit the lowest value (0.029 mm), whereas those with 1D and 2D minerals exhibit similar values (0.046 and 0.040 mm, respectively). This trend changes slightly for the thickness of the coating layer, with 3D minerals forming the thinnest layer (0.0057 mm) and the 1D and 2D minerals creating relatively thicker layers (0.061 and 0.079 mm, respectively). Notably, despite having the thinnest coating layer, 3D minerals are the most effective minerals for enhancing the tensile strength (Figure 43e,f). This suggests that even at small magnitudes of coating, 3D minerals can significantly improve the mechanical strength of separators compared to 1D or 2D minerals.

**Figure 44:**
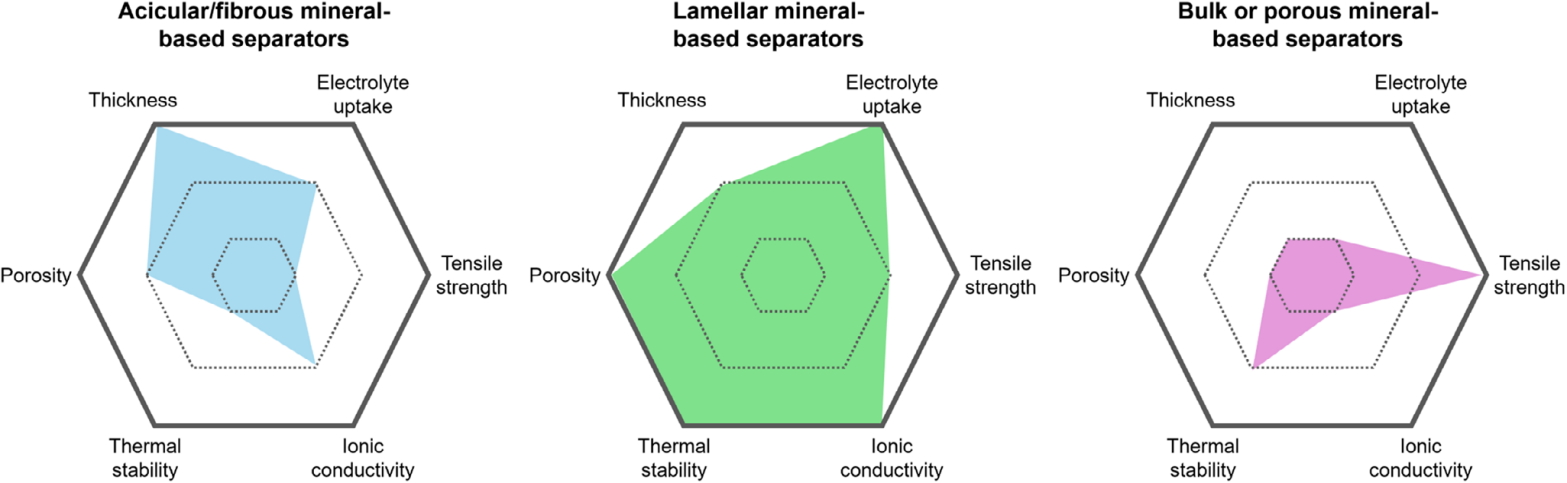
Radar plots for parameters of separators fabricated using different types of minerals.

The dependence of the separator parameters on the type of mineral coating was visualized using radar charts (**Figure** **44**). Overall, separators with 1D mineral coatings are characterized by a high thickness and moderate electrolyte uptake, porosity, and ionic conductivity. Despite their high thicknesses, these separators exhibit relatively poor mechanical strength and thermal stability. The 2D mineral coatings exhibit excellent values for most separator parameters, including sufficient mechanical strength. In contrast, 3D‐mineral‐based separators—which exhibit the highest mechanical strength and satisfactory thermal stability—are inferior to the other separators. Although the performance of the separator differs depending on the type of mineral, all three types of minerals are effective in enhancing battery performance, surpassing batteries fabricated using conventional Celgard, PP, or PE separators.

**Figure 45:**
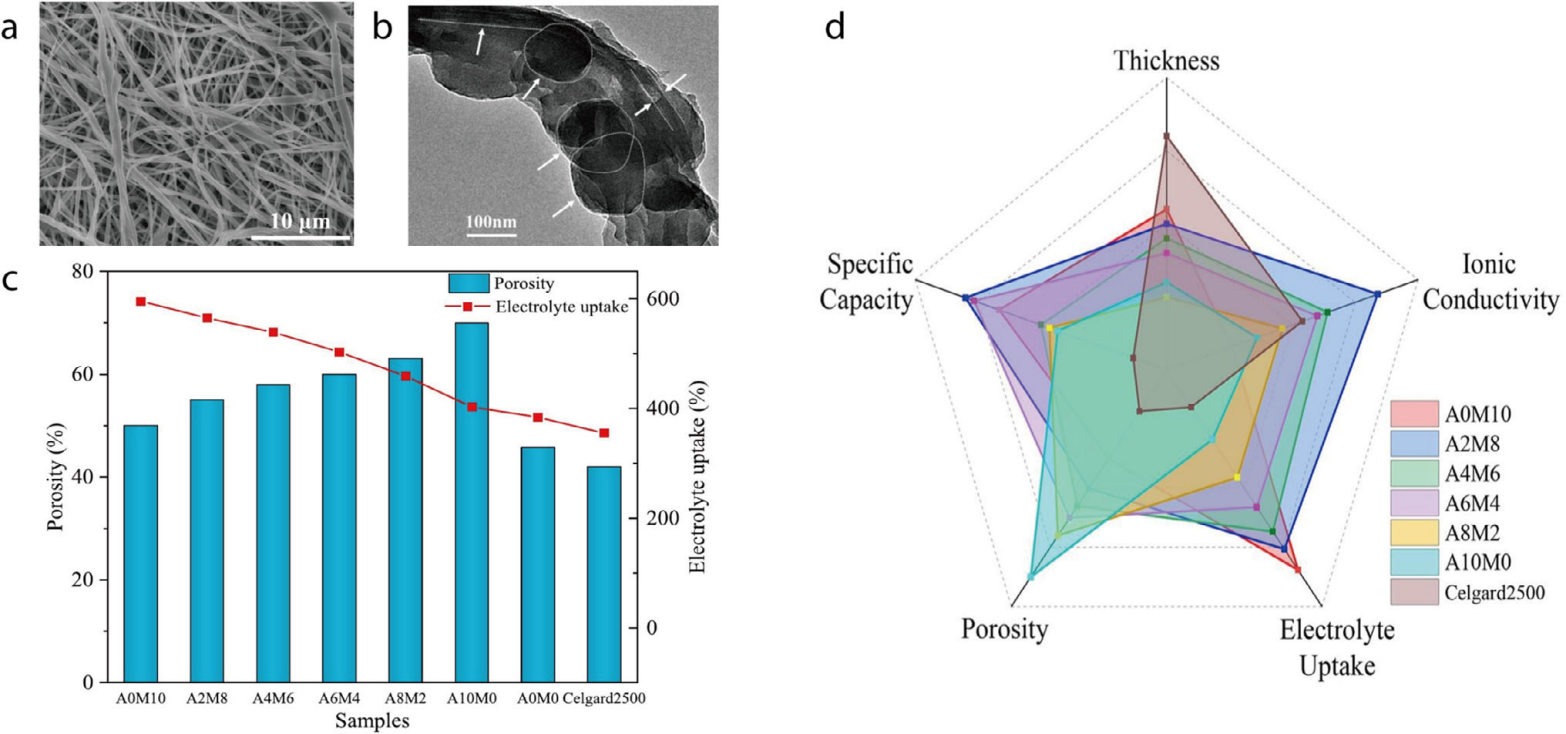
Morphology and elemental distribution of separators. a) SEM and b) TEM images of A2M8. c) Porosity and electrolyte uptake of Celgard2500 separator, A0M0 and Pal/MMT separators. d) comparison of the thickness, ionic conductivity, electrolyte uptake, porosity, and specific capacity of the Celgard 2500 and Pal/MMT separators via radar map. In these figures, A0M10, A2M8, A4M6, A6M4, A8M2, and A10M0 indicate the mineral‐modified separators with the mass ratios of Pal and MMT on separator coating were 0:10, 2:8, 4:6, 6:4, 8:2, and 10:0, respectively. Reproduced with permission.^[^ ^169^ ^]^ Copyright 2025, Elsevier.

The aforementioned results show that while mineral coatings are effective in improving the performance of separators, the enhancement is limited to certain properties depending on the type of the coated mineral. However, an ideal separator must meet all the aforementioned requirements to achieve high battery performance and durability. An effective strategy for achieving this goal is to use multiple clay minerals with complementary properties. However, current studies on such blending strategies remain limited in scale and scope, and a more systematic investigation is needed to clarify the potential and effectiveness of combining multiple clay minerals for separator design. In a related study, the composite use of minerals with differing dimensions has recently arisen as an effective method to develop high‐performance separators. One representative example is recently reported by Liu et al., where 1D palygorskite (Pal) and 2D MMT are mixed together and coated on PVDF substrate using electrostatic spinning technique.^[^ ^169^ ^]^ The resulting composite coating is composed of PVDF, MMT, and Pal, where the MMT particles are well incorporated in the fibrous Pal matrix (**Figure** **45**a,b). Note that both Pal and MMT components are located close to the surface of the separator, which allows both of them to react with the liquid electrolyte. This enables the beneficial properties of Pal (e.g., abundant hydroxyl functional groups) and MMT (e.g., lamellar structures with rapid Li transport passages) to be active in the mineral‐coated separator. Subsequent analyses on the separator performance revealed that the porosity (electrolyte uptake) linearly increases (decreases) with increasing proportions of Pal (Figure 45c). Such a linear relationship between the mass ratio and properties is also observed for the other properties, including thickness and ionic conductivity (Figure 45d). Based on this work, the authors developed the modified separator with both superior porosity and high ionic conductivity, each of which is attributed to Pal and MMT minerals, respectively. Based on these findings, one can develop a composite coating that have multiple advantages exploited from both 1D, 2D, and 3D minerals. Referring back to the radar plots in Figures 12, 32, and 42, among the low‐cost minerals reviewed in our manuscript, Dickite, MMT, and HT exhibited high ionic conductivity, whereas ATP, HT, VMT, and Zeolite showed high thermal stability, while HT, Albite, and Zeolite displayed high mechanical stability. From this perspective, a composite mineral separator synthesized from Dickite and ATP would be suitable for LiFePO_4_/graphite‐based Li‐ion batteries that require fast charge/discharge at elevated temperatures. Similarly, a separator based on MMT and HT mixture would offer both high ionic conductivity and mechanical strength, making it suitable for alloy‐type anode‐based batteries, where large volume changes must be controlled during cycling. Assuming their properties follow the rule of mixture, one can also expect composite minerals with Dickite:ATP = 2:1 or HT:MMT = 2:1 ratios could outperform conventional polyolefin‐based separators across multiple performance metrics. Likewise, identifying optimal combinations of clay minerals that can evenly enhance all separator parameters could be an important research direction in the future.

At present, governments and enterprises across worldwide countries are actively promoting the implementation of energy storage projects, where the key requirements of energy storage devices differ depending on their application scenarios. The targets of energy storage application can be roughly classified into three types: large energy storage for industrial energy supply, fast charging/discharging for electric vehicles, and high safety for household energy storage. For all cases, Li has been acting as a dominant element as carrier ions, whereas various electrode materials have been vigorously explored for desired target applications (**Table** **5**). For large energy storage application, sulfur and alloying materials in group IVA and VA elements are promising candidates for electrodes, as they offer enormous theoretical capacity (e.g., 1675^[^ ^170^ ^]^ and 4200 mAh g^−1^^[^ ^171^ ^]^ for Li–S and Li–Si batteries, respectively). However, practical use of these batteries is challenging because of their respective problems. First of all, Li–S batteries suffer from the migration of polysulfide intermediates through separators (referred as to shuttle effect), which leads to short circuit and greatly impede battery performance.^[^ ^172^ ^]^ In this case, minerals with an ample amount of functional groups can be applied to separators to effectively alleviate the shuttle effect, as they are able to capture polysulfide molecules migrating nearby. Representative mineral is MMT,^[^ ^173^ ^]^ and the other minerals, including halloysite, illite, laponite, VMT, and zeolite, can also play a similar role. For Li‐ion batteries with alloying anodes, on the other hand, huge volumetric expansions upon cycling are a key issue. For such batteries, separators are required to possess mechanical robustness as they should withstand the large volumetric changes of adjacent anodes and not be broken upon cycling. For this purpose, halloysite, albite, and HT are good choice, and several 3D minerals of boehmite and zeolite can also play an excellent role.

**Table 5:** Application scenarios of clay minerals for future batteries with three different target applications.

		Target application			
		Large energy storage		Fast charging	High safety
Battery type (electrode configuration)		Li‐ion battery (sulfur cathode, graphite anode)	Li‐ion battery (layered transition metal oxide cathodes, alloying anodes)	Li‐ion capacitor (carbonaceous electrodes), Li‐ion battery (layered transition metal oxide cathodes, graphite anode)	Li metal battery (olivine or spinel cathode, Li metal anode)
Key issues		Shuttle effect of polysulfides in sulfur cathode	Large volumetric changes of alloying anodes	Limited ionic conductivity of separators	Dendrite growth of Li metal anode, potential risk of explosion
Desired separator property		Functional groups that can capture polysulfide molecules	Mechanical strength and durability	High ionic conductivity and electrolyte uptake	Mechanical strength, flame retardancy
Recommended mineral	Cost (US$ ton^−1^) > 1,000	Laponite	Boehmite	Chrysotile, boehmite	Boehmite
	200 < Cost (US$ ton^−1^) < 1,000	Montmorillonite, halloysite, illite, vermiculite	Halloysite, hectorite	Halloysite, hectorite, montmorillonite	Halloysite, hectorite
	Cost (US$ ton^−1^) < 200	Zeolite	Albite, zeolite	Dickite	zeolite

Another target application, fast‐charging batteries, necessitates all battery components to have high ionic conductivity, which also applies to separators. In general, conventional polyolefin separators exhibit poor ionic conductivity (≈0.1 mS cm^−1^)^[^ ^174^ ^]^ compared to liquid electrolyte (1–10 mS cm^−1^)^[^ ^175^ ^]^ and can be rate limiting factor under fast charging conditions. In order for batteries to operate under high current rate, it is thus essential to develop separators with both high electrolyte uptake and ionic conductivity. Among 13 minerals surveyed in this review, six clay minerals of chrysotile, halloysite, dickite, HT, MMT, and boehmite are suitable for modified separators, as they both display superior electrolyte uptake and ionic conductivity. For the last application scenario, high safety batteries, separators should be sturdy enough to prevent short circuit while possessing flame retardancy to avoid potential explosion under extreme environment. Such requirements are particularly critical for batteries employing Li metal anodes, as they suffer from inherent Li dendrite growth and associated explosion. To prevent these issues, mineral‐based separators with mechanical durability and heat resistance are of great help, particularly those modified by halloysite, HT, boehmite, and zeolite.

To fully take advantage of these mineral‐based separators, precise pretreatment and surface modification are essential. Otherwise, the impurities within the minerals decrease the porosity and specific surface area, leading to poor battery performance in practical applications. For instance, KL or halloysite mined from kaolin deposits are often mixed with fine iron‐containing impurities such as mica or illite.^[^ ^176^ ^]^ In addition, most 2D minerals with hydrated cations tend to filled with crystalline water under high‐humidity environments.^[^ ^177^ ^]^ If such minerals are used in separators without pretreatment, the impurities can reduce porosity and hinder Li‐ion transport. For these reasons, previous studies have employed acid or heat treatments to remove impurities before applying minerals to separators. In case of acid treatment, it typically uses sulfuric acid or hydrochloric acid to dissolve impurities within clay minerals. This method has been widely applied to halloysite^[^ ^116^ ^]^ and MMT,^[^ ^178^ ^]^ and it is known to enhance the surface area and porosity of the minerals. Unlike acid treatment, which dissolves solid impurities, heat treatment is used when the natural mineral contains crystalline water. A representative example is ATP. Sun et al. demonstrated that heat treatment at 400 °C could remove crystalline water while preserving the fibrous structure of ATP, thereby increasing its active surface area that can capture polysulfides in Li–S batteries.^[^ ^35^ ^]^

In addition to the acid and heat pretreatment methods mentioned in the manuscript, previous studies also employed rather physical methods to remove impurities from clay minerals. Representative techniques include dispersing clay minerals in water and using ultrasonication or centrifugation to separate impurities. In related studies, ultrasonication has been used to remove Fe and Ti oxides embedded within kaolinite, and centrifugation has been employed to extract high‐grade MMT from bentonite.^[^ ^179^, ^180^, ^181^ ^]^ More recently, dedicated efforts have been made to apply multiple pretreatment steps in sequence to achieve higher‐purity clay minerals.^[^ ^182^, ^183^ ^]^ For example, Xu et al. implemented a three‐step purification process to obtain pure attapulgite: 1) ultrasonic hydrothermal method, 2) acid treatment, and 3) heat treatment with reducing agents.^[^ ^182^ ^]^ This process resulted in a rod‐like attapulgite mineral with a purity of 99.9%. These results indicate that combining multiple pretreatment techniques can significantly minimize impurities in clay minerals. However, such complex purification processes sacrifice the economic advantage of clay minerals. The price of purified attapulgite (≈$420 ton^−1^) is over eight times higher than that of untreated attapulgite (≈$50 ton^−1^). Therefore, there is a clear need for systematic research into simpler pretreatment methods that can still yield minerals with sufficient purity for application in battery separators.

Furthermore, naturally occurring porous structures are not limited to clay minerals. Various natural materials, such as wood, marine shells, and biopolymers, have hierarchical and porous structures, which can be analyzed and utilized to expand the scope of applications beyond separators to electrode active materials and conductive additives. In future research, we will reconstruct the internal microstructure of wood samples by 3D tomography to identify structure‐performance correlation, and expand the scope of application to not only separators but also electrode active materials and conductive additives based on the obtained bio‐based porous structure information.

## Conflict of Interest

The authors declare no conflict of interest.
